# Glucogenic and lipogenic diets affect *in vitro* ruminal microbiota and metabolites differently

**DOI:** 10.3389/fmicb.2022.1039217

**Published:** 2022-12-16

**Authors:** Dengke Hua, Wouter H. Hendriks, Yiguang Zhao, Fuguang Xue, Yue Wang, Linshu Jiang, Benhai Xiong, Wilbert F. Pellikaan

**Affiliations:** ^1^State Key Laboratory of Animal Nutrition, Institute of Animal Sciences, Chinese Academy of Agricultural Sciences, Beijing, China; ^2^Animal Nutrition Group, Department of Animal Sciences, Wageningen University and Research, Wageningen, Netherlands; ^3^Beijing Key Laboratory for Dairy Cattle Nutrition, Beijing Agricultural College, Beijing, China

**Keywords:** glucogenic/lipogenic diet, rumen fermentation, microbiota, gas production, metabolomics, PICRUSt

## Abstract

This study was conducted to evaluate the effects of two glucogenic diets (C: ground corn and corn silage; S: steam-flaked corn and corn silage) and a lipogenic diet (L: sugar beet pulp and alfalfa silage) on the ruminal bacterial and archaeal structures, the metabolomic products, and gas production after 48 h *in vitro* fermentation with rumen fluid of dairy cows. Compared to the C and S diets, the L dietary treatment leaded to a lower dry matter digestibility (DMD), lower propionate production and ammonia-nitrogen concentration. The two glucogenic diets performed worse in controlling methane and lactic acid production compared to the L diet. The S diet produced the greatest cumulative gas volume at any time points during incubation compared to the C and L diet. The metabolomics analysis revealed that the lipid digestion especially the fatty acid metabolism was improved, but the amino acid digestion was weakened in the L treatment than in other treatments. Differences in rumen fermentation characteristics were associated with (or resulting from) changes in the relative abundance of bacterial and archaeal genera. The rumen fluid fermented with L diet had a significantly higher number of cellulolytic bacteria, including the genera of *Ruminococcus*, *Butyrivibrio*, *Eubacterium*, *Lachnospira*, unclassified *Lachnospiraceae*, and unclassified *Ruminococcaceae*. The relative abundances of amylolytic bacteria genera including *Selenomonas_1*, *Ruminobacter*, and *Succinivibrionaceae_UCG-002* were higher in samples for diets C and S. The results indicated that the two glucogenic diets leaded to a higher relative abundance of bacteria which functions in succinate pathway resulting in a higher propionate production. The steam-flaked corn diet had a higher gas production and lower level of metabolites in fatty acids and amino acids. Most highly abundant bacteria were observed to be not sensitive to dietary alterations of starch and fiber, except for several amylolytic bacteria and cellulolytic bacteria. These finding offered new insights on the digesting preference of ruminal bacteria, which can assist to improve the rumen functioning.

## Introduction

Dietary carbohydrates, such as starch and fiber, provide substrates for rumen microbes. Changes in carbohydrate composition and content in ruminant rations lead to the changes in microbial community and subsequently to changes in fermentation end-products, including the volatile fatty acids (VFAs), carbon dioxide (CO_2_), methane (CH_4_), and hydrogen (H_2_; Carberry et al., 2012). The major ruminal VFAs include acetate, propionate, and butyrate. Acetate is the primary precursor of milk fatty acids and is termed a lipogenic nutrient, while propionate being the primary precursor of milk lactose is a glucogenic nutrient (van Knegsel et al., 2005). For ruminant animals, lipogenic nutrients in metabolism are supplied by acetate and butyrate from ruminal degradation of fiber or dietary fat, if not derived from the mobilization of body fat reserves (van Knegsel et al., 2005). In contrast, glucogenic nutrients in metabolism can originate from the ruminal fermentation of dietary starch to propionate, rumen bypass of dietary starch which is then digested in the small intestine and absorbed as glucose, or gluconeogenesis (Van Knegsel et al., 2007). Previous research showed that glucogenic nutrients increased plasma glucose and insulin concentrations, whereas lipogenic nutrients did not (van Knegsel et al., 2005). However, changes in microbial communities and their metabolic activities under lipogenic and glucogenic diets are also essential to investigate to unravel the production pathways of the affected metabolites and better understand how rumen functioning is regulated.

Ground corn is a major dietary energy source because of its high amount of readily fermentable starch. Steam-flaking can disintegrate the crystalline structure of cereal starch by gelatinisation (Ding et al., 2007), and subsequently, this can increase the accessibility to the starch granules of ruminal amylases and amylolytic microorganisms (Huntington, 1997). Previous studies showed that steam-flaked corn improved the ruminal degradability of starch, resulting in high production of ruminal propionate, and increased efficiency in microbial protein synthesis (Zhou et al., 2015). However, the alteration in rumen microbial communities underneath these improvements is still not clear, which is essential for manipulate the starch degradation in the rumen.

The effects of dietary treatments on the ruminal microbes and microbial metabolism when incubated *in vitro* are rarely reported although the *in vitro* gas production technique is routinely used to evaluate dry matter (DM) degradation rate, amount and proportion of VFAs production, and gas composition of various feeds and ingredients. This technique also yields valuable information on the effects of feedstuff on rumen microbial activity and predicts the kinetics of fermentation (Pellikaan et al., 2011a). With the 16S rRNA sequencing technology, a fast and cost-effective way of microbial analysis and their correlations with environmental factors coupled with liquid chromatography-mass spectrometry (LC–MS), an effective technique for metabolomics analysis, more knowledge on changes in ruminal microbiota metabolism can be generated (Zhang J. et al., 2017).

Although studies on differences in rumen fermentation between glucogenic and lipogenic diets have been conducted, the changes in rumen bacterial community and functions are not yet fully understood. We hypothesized that glucogenic and lipogenic diets when evaluated using the *in vitro* gas production technique should lead to clear differences in bacterial communities and functions which affect intermediary metabolites besides the well-known differences in fermentation end-products and CH_4_ production.

## Materials and methods

### Experimental design

Animal care followed the Chinese guidelines for animal welfare, and all protocols were approved by the Animal Care and Use Committee of the Chinese Academy of Agricultural Sciences (IAS2019-6).

Three total mixed rations (TMR) were designed: two glucogenic diets including a ground corn diet (C, which used ground corn and corn silage as the primary energy sources), a steam-flaked corn diet (S, which used steam-flaked corn and corn silage as the primary energy sources), and a lipogenic diet (L) mainly containing sugar beet pulp and alfalfa silage as the energy sources. In addition, other ingredients, including soybean meal, oat and alfalfa hay, and calcium hydrogen phosphate were used to balance the ration to meet the nutritional requirements of dairy cattle (Table 1). Diets were isocaloric and were equal in the digestible crude protein.

**Table 1:** Ingredient and nutritional composition of two glucogenic (C, S) and a lipogenic (L) diet.

Item	Experimental diet		
	C	L	S
Ingredient, % of dry matter			
Ground corn	28	0	0
Sugar beet pulp	0	28	0
Steam flaked corn	0	0	28
Soybean meal	18.5	12	18.5
Oat hay	5	19	5
Alfalfa hay	10	10	10
Corn silage	38	0	38
Alfalfa silage	0	30	0
Dicalcium phosphate	0.5	1	0.5
Composition, g/kg of dry matter			
Crude protein	174.4	174.6	172.1
Ether extract	24.3	20.4	31.7
Starch	192.9	39.7	163.8
Neutral detergent fiber	326	562.2	320.2
Acid detergent fiber	197.9	348.9	199.1
Ash	47.6	98.7	47
Calcium	9.3	12.8	11.1
Phosphorus	10.4	4.9	11.9
NE_L_, MJ/kg of dry matter	7.3	7.9	7.4

Nutrient composition of the experimental diets was calculated according to National Research Council (NRC) (2001). Diets: C, corn and corn silage diet; L, sugar beet pulp and alfalfa silage diet; and S, corn and steam-flaked corn diet. NE_L_, net energy for lactation and calculated according to National Research Council (NRC) (2001).

### *In vitro* incubation

Six lactating dairy cows (Holstein) were selected as rumen fluid donors for all three runs of this *in vitro* study, with different two cows for each run. The cows received a diet containing (% DM basis) a concentrated mixture (45%), alfalfa and oat hay (20%), corn silage (20%), and alfalfa silage (15%). The cows were fed three time daily at 7:00, 13:00, and 19:00, and they had free access to water and feed.

The fermentation substrates were the ground DM of each experimental diets. 0.5 g of substrates were firstly weighed into 150-ml serum bottles, with three replicate bottles for each dietary treatment within one fermentation run. A phosphate-bicarbonate buffer medium was anaerobically prepared as described by Menke and Steingass (1988).

Equal volumes of fresh ruminal fluid were collected through a stomach tube from two cows in each fermentation run, 2 h after the first feeding (09:00 h), then poured into a sterilized and pre-warmed thermos flask (2,000 ml) leaving no headspace in the flask. After transportation to the laboratory, the rumen fluid was strained through four layers of cheesecloth and transferred into a flask placed in a water bath of 39°C maintaining anaerobic conditions. The strained rumen fluid inoculum (25 ml) and anaerobic buffer (50 ml) were successively combined with substrates into each bottle, with the CO_2_ continuously flushing in the headspace of bottles. After sealing with butyl rubber stoppers, the serum bottles were connected to the gas inlets of an automated gas production recording system (AGRS), as reported by Zhang and Yang (2011). Each fermentation run lasted for 48 h and was repeated for three runs within 2 weeks (Supplementary Figure S1).

### Sampling and chemical analysis

Calibrated gas volumes were automatically recorded and cumulative gas production was expressed against the time of incubation. At 48 h of incubation, 20 μl of gas was collected through a 20 μl gastight syringe from each bottle to test the CH_4_ concentration using gas chromatography (GC, 7890B, Agilent Technologies, United States). The GC was equipped with a capillary column (USF727432H, 30 m × 0.25 mm × 0.25 μm, Agilent, California, United States) and a flame ionization detector (FID). Nitrogen (N_2_, 99.99%) was used as the carrier gas, with column settings as follows: the inlet pressure 18.85 psi, the total flow 30.2 ml/min, the column flow 1.7 ml/min, the linear speed 39.8 cm/s, the split ratio 15, the sweeping flow 3 ml/min, and the cycling flow 8 ml/min. The hydrogen and airflow were 40 ml/min and 400 ml/min, respectively. Temperatures were set to 100°C for the injection point, 80°C for the column oven, and 120°C for the detector.

At 48 h, all bottles were transferred into an ice-water mixture to terminate the incubation. The pH value of the fermented substrates was determined using a portable pH meter (PHB-4, INESA, Shanghai, China). Then the substrates were filtered through a nylon bag (12 cm × 8 cm i.d. and 50 μm of pore size) and the residue left in the bag was used to analyze apparent dry matter digestibility (DMD) gravimetrically. Two replicates of 3 ml fluid samples were quickly collected into two cryogenic vials and immediately frozen in liquid nitrogen until being stored at −80°C, which were used for 16S rRNA sequencing and metabolomics analysis separately. A sample of 1 ml fluid was mixed with 0.25 ml of 25% meta-phosphoric acid to evaluate the VFA contents *via* the GC (7890B, Agilent Technologies, United States; Mao et al., 2008). Also, 1 ml of fluid was collected to analyze the ammonia nitrogen (NH_3_-N) according to the indophenol way (Broderick and Kang, 1980) and another 1 ml fluid was used to measure the lactic acid concentration by a commercial kit (A019-2, Nanjing Jiancheng Bioengineering Institute, Nanjing, China; Pan et al., 2016). Two replicated 3 ml fluid samples were stored under −80°C for later microbial and metabolomics analysis, separately.

### DNA extraction and amplification

The DNA of microbes was extracted from supernatant samples using the QIAamp DNA Stool Mini Kit (M5635-02, OMEGA, United States). The concentration of DNA was evaluated with the Nano Drop spectrophotometer (Thermo Scientific, United States), and then the agarose gel (1% w/v) electrophoresis was used to check the DNA quality.

The 16S rRNA gene of bacteria and archaea were separately amplified with the general primers (Supplementary material) based on the hypervariable region (V3–V4). The PCR was performed (Supplementary material) and the products were firstly extracted from an agarose gel (2% w/v), then purified with the commercial Extraction Kit (Axygen Biosciences, United States). The DNA products were finally quantified with QuantiFluor™-ST (Promega, United States).

### Illumina miSeq sequencing and analysis

Purified amplicons were mixed in an equimolar ratio and paired-end sequenced (2 × 300 bp) through the MiSeq platform (Illumina, San Diego, United States) according to the manufacturer’s standard (Majorbio Bio-Pharm Technology Co. Ltd., Shanghai, China).

The raw fastq was quality-filtered with FLASH following the protocol previously reported by Pan et al. (2017). With a 97% sequence similarity cut-off, the operational taxonomic units (OTUs) were clustered through UPARSE. The taxonomy was calculated with the RDP Classifier against the SILVA (SSU123) 16S rRNA database with a confidence threshold of 70%. The principal coordinates analysis (PCoA) was analyzed with the method of unweighted UniFrac distance to compare the interrelationships of bacterial communities between diets using the R software (3.4.4). The community richness and diversity were analyzed by the alpha diversity indexes including the OTU, Chao 1, ACE, Shannon, and Simpson (Hua et al., 2021).

### Inferred metagenomics analysis

The metagenome functions of ruminal bacteria were predicted using the analysis of Phylogenetic Investigation of Communities by Reconstruction of Unobserved States (PICRUSt). Firstly, the closed OTU table was normalized by the 16S rRNA copy number whereafter the results were exported into the Kyoto Encyclopedia of Genes and Genomes (KEGG) pathways. The PCoA was conducted to calculate the similarities of the predicted functions among groups by the R software (3.4.4). The top 10 abundant functions were further analyzed to determine significant differences among diets using Welch’s *t*-test in R software (3.4.4).

### Metabolomics processing

The method was modified from the procedure described by Wang et al. (2021). The rumen fluid samples were firstly thawed under room temperature whereafter 200 μl supernatant of each sample was collected into a 1.5 ml centrifuge tube and mixed with 800 μl extracting solution [methanol: acetonitrile = 1:1 (v/v)]. Each sample was then vortexed for 30 s and extracted ultrasonically (40 kHz) at 5°C for 30 min before being treated under −20°C for 30 min. All samples were centrifuged (13,000 × *g*, 4°C, 15 min) and the supernatant transferred to a new tube, mixed with 100 μl acetonitrile solution (acetonitrile: water = 1:1), vortexed for 30 s, extracted ultrasonically (40 kHz) at 5°C for 5 min, and centrifuged (13,000 × *g*, 4°C, 10 min) where after 200 μl of the supernatant was carefully transferred to sample vials for LC–MS/MS analysis. At the same time, 20 μl of supernatant was collected from each sample and mixed as the quality control sample (QC) which were injected at regular intervals throughout the analytical runs in order to obtain system repeatability.

Chromatographic separation of the metabolites was performed on the ultra-performance liquid chromatography (UPLC) coupled with a triple time-of-flight (TOF) system (UPLC-Triple TOF, AB Sciex, United States). The system was equipped with the ACQUITY UPLC HSS T3 column (100 mm × 2.1 mm id, 1.8 μm; Waters, Milford, United States). Mobile phase A consisted of 5% acetonitrile water and 0.1% formic acid, the mobile phase B contained 95% acetonitrile-isopropanol (1:1, v/v) and 0.1% formic acid. The injection volume was 10 μl, the flow rate was 0.4 ml/min, and the column temperature was 40°C. The elution gradient of the mobile phases was shown in the Supplementary material. After being treated with an electrospray ionization (ESI) source, the signals of mass spectra were scanned in both positive mode and negative mode. The optimal conditions for mass spectra were shown in the Supplementary material.

### Metabolomics data analysis

After UPLC-TOF/MS analyses, the raw data were imported into Progenesis QI 2.3 (Waters Corporation, Milford, United States) for a series of pre-processing, including filtration of the baseline, identification and integration of the peak, correction of the retention time, and alignment of the peak. After the pre-processing, a data matrix was generated consisting of the retention time (RT), mass-to-charge ratio (m/z) values, and peak intensity. The MS and MS/MS information was searched in the Human metabolome database (HMDB; http://www.hmdb.ca/) and Metlin database.^1^ Results were shown in the form of a data matrix.

After being pre-processed, the data matrix was analyzed on the Majorbio Cloud Platform.^2^ Using the R package of ROPLS (version1.6.2), PCA was applied to obtain an overview of the metabolic data, general clustering, trends, or outliers among groups whereafter orthogonal partial least squares discriminate analysis (OPLS-DA) was performed to observe the global difference of the metabolites between comparable groups. The variable importance in the projection (VIP) was calculated in the OPLS-DA model, and the *p*-value was estimated with paired Student’s *t*-test. Statistically affected metabolites among groups were selected with VIP > 1 and *p* ≤ 0.05. The affected metabolites between every two groups were summarized into different metabolic groups and mapped into their biochemical pathways through the KEGG database. The metabolic pathway enrichment analysis of the metabolic groups was conducted with the Fisher’s exact test using the Python package of Scipy. stats (version1.0.0, SciPy.org).

### Correlation between bacterial community and rumen metabolites

Correlation between the affected bacterial genera with a relative abundance >0.5% and the rumen fermentation parameters, as well as the correlation between these affected bacterial genera and the differential metabolites (VIP > 1.5, fold change >2 or < 0.5, *p* ≤ 0.05), was separately assessed by Pearson’s correlation analysis in R (version 3.4.4). These correlations were visualized using the R package of Pheatmap.

### Curve fitting and calculations

Data of the cumulative gas production curve were in accordance with the monophasic model using a non-linear least squares regression procedure NLIN in SAS 9.3 (SAS Institute Inc., Cary, NC, United States; Pellikaan et al., 2011b):


$$GP=\frac{A}{1+{\left(\frac{C}{t}\right)}^{B}\;}$$

in which GP is the total gas produced (ml/g OM), A is the asymptotic gas production (ml/g OM), B equals the switching characteristic of the curve, and C is the time at which half of the asymptote has been reached and t is the time (h). The maximum rate of gas production (R_max_, ml/g OM/h) and the time when R_max_ appears (TR_max_, h) were separately calculated using the equations below:


$$R_{\text{max}}=\frac{A\times C^{B}\times B\times {{TR}_{\text{max}}}^{\left(-B-1\right)}\;}{\;{\left(1+\left(C^{B}\times {{TR}_{\text{max}}}^{\left(-B\right)}\right)\right)}^{2}}$$

$${TR}_{\text{max}}=C\times {\left(\frac{B-1}{B+1}\right)}^{\frac{1}{B}}$$

### Statistical analysis

All fermentation end-products and gas kinetics data were analyzed using PROC MIXED of SAS 9.3 (SAS Institute Inc., Cary, NC, United States). The statistical model was


$$Y_{ij}=\mu +D_{i}+B_{j}+e_{ij}$$

where *Y*_ij_ is the dependent variable, μ is the overall mean, D_i_ is the fixed effect of diet (*i* = 1–3), B_j_ is the random effect of run (*j* = 1–3), e_ij_ is the random residual error. The Student–Newman–Keuls (SNK) multiple comparison procedure in the LSMEANS statement was used to test differences among treatments. Significance was considered at *p* ≤ 0.05, and a trend was declared at 0.05 < *p* ≤ 0.10.

## Results

### Effect of treatments on gas production

The cumulative gas productions at 6, 12, 24, and 48 h of *in vitro* incubation showed the same direction of effects (Table 2), where the diet S treatment had the highest gas production compared to the other two treatments, while the diet L treatment gave the lowest gas production (*p* < 0.001). The CH_4_ production for diet S was higher than that for diet L (*p* = 0.043), but both diets did not differ from the diet C. The *in vitro* DMD for diets C and S was greater (*p <* 0.001) than that for diet L.

**Table 2:** Comparison of cumulative gas production at 6, 12, 24, and 48 h, curve fit parameters, head space methane concentration, and dry matter digestibility at 48 h among two glucogenic (C, S) and a lipogenic (L) diet under *in vitro* fermentation with rumen fluid of dairy cows.

Item	Experimental diet			SEM	*p* value
	C	L	S		
Gas production (ml/g OM)					
6 h	94.37^b^	73.06^c^	106.08^a^	3.783	<0.001
12 h	118.14^b^	100.08^c^	132.45^a^	3.671	<0.001
24 h	125.67^b^	108.68^c^	139.29^a^	3.561	<0.001
48 h	128.24^b^	110.95^c^	141.40^a^	3.464	<0.001
Curve fit parameters					
A (ml/g OM)	139.77^b^	124.60^c^	155.84^a^	3.438	<0.001
B	1.41^a^	1.18^c^	1.36^b^	0.04	0.001
C (h)	3.91^b^	4.22^a^	3.57^c^	0.129	<0.001
Rmax (ml/h/g OM)	23.48^c^	24.49^b^	27.83^a^	0.936	<0.001
TRmax (h)	1.03^a^	0.51^c^	0.88^b^	0.1	<0.001
Methane (%, 48 h)	11.64^ab^	9.23^b^	13.45^a^	0.844	0.043
DMD (%, 48 h)	87.72^a^	75.82^b^	87.64^a^	0.979	<0.001

A, asymptotic gas production; B, switching characteristic of the curve; C, time at which half of the asymptote has been reached; Rmax, maximum rate of gas production; TRmax, time at which Rmax occurs. DMD, dry matter digestibility. Diets: C, corn and corn silage diet; L, sugar beet pulp and alfalfa silage diet; and S, steam-flaked corn and corn silage diet. OM, organic matter. SEM, standard error of the mean. ^a,b,c^means within a row with different superscripts differ significantly (*p* ≤ 0.05).

The cumulative gas production curve derived from the monophasic model is shown in Supplementary Figure S2. As for the curve fit parameter estimates (Table 2), the S treatment had the highest asymptotic gas production (A) compared to the other treatments (*p* < 0.001), the switching characteristic (B) of diet L treatment was lower, while diet S treatment had lower halftime (C), compared to the other two diets (*p* < 0.001). The S treatment had the highest maximum gas production rate (*R*_max_) followed by treatment L and treatment C (*p* < 0.001).

### Effect of treatments on fermentation end-products

The values of fermentation end-products and pH at 48 h are shown in Table 3. Compared with the L treatment, both C and S treatments resulted in higher lactic acid concentration (*p* = 0.011) but lower pH value (*p* < 0.001). The fermentation end-products for diet L possessed a significantly lower NH_3_-N concentration (*p* = 0.001) and a lower lactic acid level (*p* = 0.011). Both C and S diets leaded to greater propionate (*p* = 0.004) and butyrate (*p* = 0.015) concentrations and lower acetate to propionate ratio (*p* < 0.001) in the fermentation end-products compared to the L diet.

**Table 3:** Effect of two glucogenic (C, S) and a lipogenic (L) diet on the ruminal pH and end-products after 48 h *in vitro* fermentation with rumen fluid.

Item	Experimental diet			SEM	*p*value
	C	L	S		
pH	6.61^b^	6.74^a^	6.62^b^	0.011	<0.001
NH_3_-N (mg/dl)	70.14^a^	52.98^b^	65.70^a^	1.817	0.001
Volatile fatty acids (mmol/l)					
Acetate	68.9	71.87	71.29	1.129	0.65
Propionate	27.58^a^	24.42^b^	29.34^a^	0.582	0.004
Acetate:Propionate	2.50^b^	2.94^a^	2.43^b^	0.037	<0.001
Butyrate	11.86^a^	10.42^b^	12.31^a^	0.258	0.015
Valerate	0.68	0.6	0.71	0.025	0.356
Isobutyrate	5.66	5.31	5.81	0.111	0.306
Isovalerate	7.34	6.66	7.39	0.165	0.208
Total VFA	124.5	122.2	129.3	2.1	0.492
Lactic acid (mmol/l)	0.51^a^	0.40^b^	0.50^a^	0.016	0.011

Diets: C, corn and corn silage diet; L, sugar beet pulp and alfalfa silage diet; S, steam-flaked corn and corn silage diet. SEM, standard error of the mean. Total VFA, total volatile fatty acid (acetate + propionate + butyrate + valerate + isobutyrate + isovalerate). ^a,b^ means within a row with different superscripts differ significantly (*p* ≤ 0.05).

### Effect of treatments on ruminal bacteria and archaea

The alpha diversity parameters of both the ruminal bacteria and archaea as influenced by the three dietary treatments are shown in Table 4. A total of 1,070,928 quality sequence reads across all samples were acquired with an average read length of 421 bp. The total number of reads from each sample varied from 28,949 to 70,861, with an average reads number of 38,919. The entire sequences were assigned to 2,042 OTUs using a cut-off of 97% sequence similarity. The richness and diversity estimators (Table 4) showed the total number of observed OTUs out of the rumen fluid in the L treatment was higher than in the other treatments (*p* = 0.031). No differences in other diversity estimators (Chao 1, ACE, Shannon, and Simpson indices) were observed among the three groups. The alpha diversity estimates of archaea (Table 4) showed that the total number of observed OTUs for the C diet was lower compared to the S and L diets (*p* = 0.028). Both the C and S diets leaded to a significantly lower Shannon diversity index and a higher Simpson diversity index for archaea in comparison with the L diet (*p* = 0.024).

**Table 4:** Effect of two glucogenic (C, S) and a lipogenic (L) diet on the alpha diversity indices of ruminal bacteria and archaea communities after 48 h *in vitro* fermentation with rumen fluid of dairy cows.

Item	Experimental diet			SEM	*p* value
	C	L	S		
Bacteria					
OTU	1,403^b^	1,493^a^	1,408^b^	9.377	0.031
Chao 1	1,652	1,717	1,655	6.734	0.241
ACE	1,652	1,717	1,655	6.734	0.136
Shannon	5.58	5.78	5.56	0.026	0.339
Simpson	0.021	0.012	0.025	0.001	0.295
Archaea					
OTU	152^c^	182^a^	173^b^	4.114	0.028
Chao 1	306	352	364	8.095	0.056
ACE	518	533	590	10.201	0.427
Shannon	1.10^b^	1.57^a^	1.17^b^	0.068	0.018
Simpson	0.584^a^	0.357^b^	0.539^a^	0.032	0.024

Diets: C, corn and corn silage diet; L, sugar beet pulp and alfalfa silage diet; S, steam-flaked corn and corn silage diet. ACE, abundance-based coverage estimator. OTU, operational taxonomic units. SEM, standard error of the mean. ^a,b,c^ means within a row with different superscripts differ significantly (*p* ≤ 0.05).

**Figure 1:**
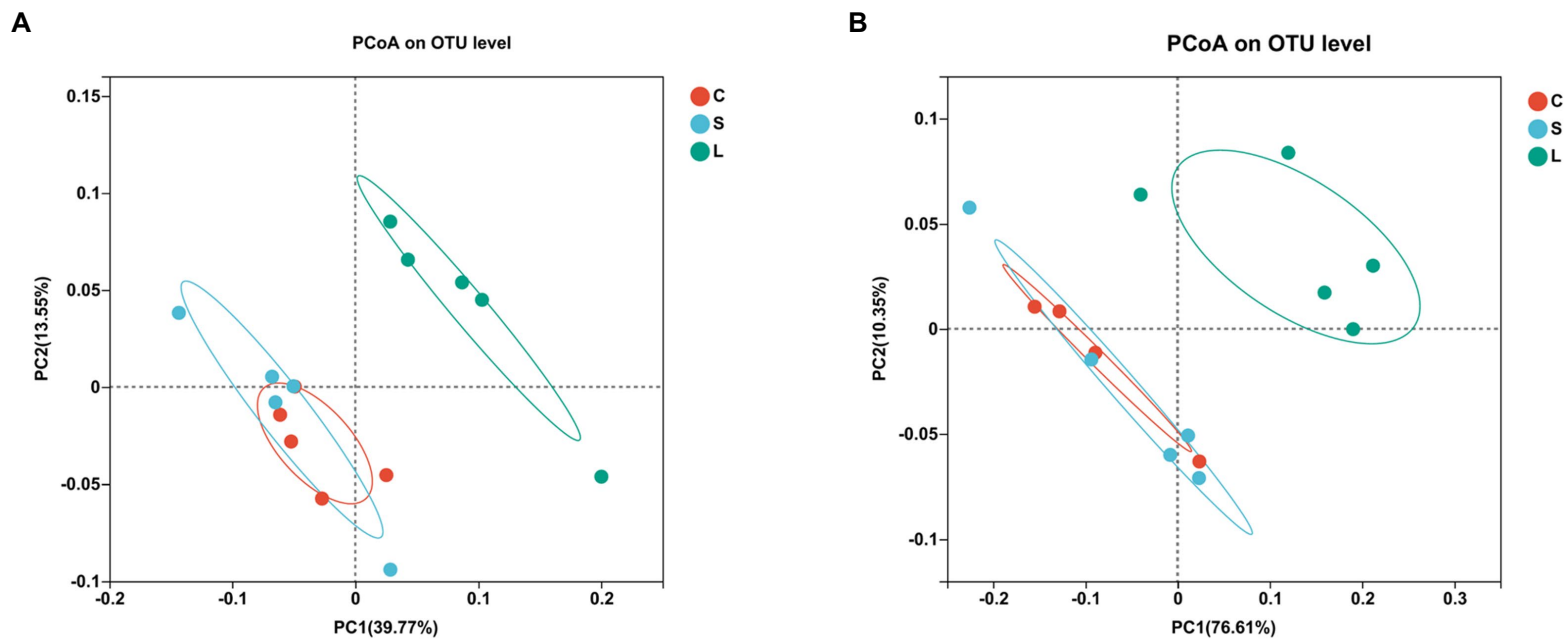
Principal coordinate analysis (PCoA) of the ruminal bacteria **(A)** and archaea **(B)** communities on OTU level among two glucogenic (C, S) and a lipogenic (L) diet after 48 h *in vitro* fermentation with rumen fluid of dairy cows. Diets: C, corn and corn silage diet; L, sugar beet pulp and alfalfa silage diet; and S, steam-flaked corn and corn silage diet.

To visualize the impact of the diets on overall rumen bacteria and archaea communities, a PCoA was performed (Figures 1A,B). The rumen bacterial community showed a clear separation between the S and L dietary treatment along PC1, explaining >39% of the total variation, and the L diet was separated from the C diet along PC2, explaining >20% of the total variation (Figure 1A). The ruminal archaea communities in the rumen fluid samples from the L diet were clearly distinguished from those in the C and S diets, with approximately 76% of the variance explained along PC1 (Figure 1B).

A total of 21 bacterial phyla were identified from all samples among dietary treatments, with Bacteroidetes, Firmicutes, and Proteobacteria being the top three predominant phyla, representing 45.0–49.4, 36.1–41.6, and 3.9–6.5% of all sequences, respectively (Supplementary Table S1). The rumen fluid samples from L treatment showed a higher relative abundance of Tenericutes than samples from the other two treatments (*p* = 0.042).

At the genus level, a total of 176 bacterial genera were identified which together accounted for 96% of all sequences. 89 of these identified genera with a relative abundance of ≥0.1% in at least one sample were further analyzed. Among all genera, 26 genera were affected by dietary treatments (Supplementary Table S2), with the top 20 of these genera listed in Table 5. 12 of these affected genera had higher relative abundances in the rumen fluid for L diet compared to the other two diets, including *SP3-e08* (*p* = 0.011), *Christensenellaceae_R-7_group* (*p* = 0.029), *Ruminococcaceae_UCG-014* (*p* = 0.026), *Family_XIII-AD3011_group* (*p* = 0.004), unclassified*_o_Clostridiales* (*p* = 0.010), *Selenomonas_1* (*p* = 0.005), *Lachnospiraceae_ND3007_group* (*p* = 0.025), *[Eubacterium]_coprostanoligenes_group* (*p* < 0.001), unclassified*_f_Lachnospiraceae* (*p* = 0.014), unclassified*_f_Ruminococcaceae* (*p* = 0.001), *Ruminococcaceae_UCG_013* (*p* = 0.006), *Ruminococcus_1* (*p* = 0.022), *Butyrivibrio_2* (*p* = 0.037), *[Eubacterium]_oxidoreducens_group* (*p* = 0.044), and *Family_XIII_UCG-002* (*p* = 0.026). However, the relative abundances of *Ruminococcus_2* (*p* = 0.018), *Ruminobacter* (*p* < 0.001), and *Succinivibrionaceae*_*UCG-002* (*p* = 0.004) were lower for the L dietary treatment. The S dietary treatment resulted in a greater relative abundance of *Selenomonas_1* (*p* = 0.005), while lower relative abundances of *Family_XIII_UCG-002* (*p* = 0.026) and *Family_XIII_ AD3011_group* (*p* = 0.004) compared to the other two diets. Compared with the S dietary treatment, the rumen fluid for the C treatment had a higher relative abundance of the *Family_XIII_AD3011_group* (*p* = 0.004), *Family_XIII_UCG-002* (*p* = 0.026), and *Succinivibrionaceae_UCG-002* (*p* = 0.004).

**Table 5:** Effect of two glucogenic (C, S) and a lipogenic (L) diet on the relative abundance (%) of the top 20 affected bacteria and the top three differential archaea at the genus level after 48 h *in vitro* fermentation with rumen fluid of dairy cows.

Domain/Phylum	Genus/others	Experimental diet			SEM	*p* value	C	L	S
Bacteria						
Bacteroidetes	*SP3-e08*	0.11^b^	0.17^a^	0.10^b^	0.009	0.011
Firmicutes	*Christensenellaceae_R-7_group*	1.28^b^	1.70^a^	1.06^b^	0.077	0.029
	*Ruminococcus_2*	0.87^a^	0.55^b^	1.0^a^	0.055	0.018
	*Ruminococcus_1*	0.18^b^	0.31^a^	0.18^b^	0.068	0.022
	*Ruminococcaceae_UCG-014*	0.74^b^	1.05^a^	0.70^b^	0.045	0.026
	*Ruminococcaceae_UCG-013*	0.18^b^	0.35^a^	0.15^b^	0.026	0.006
	*Unclassified_f_Ruminococcaceae*	0.19^b^	0.35^a^	0.20^b^	0.021	0.001
	*Butyrivibrio_2*	0.24^b^	0.62^a^	0.29^b^	0.076	0.037
	*Selenomonas_1*	0.38^b^	0.26^b^	0.88^a^	0.077	0.005
	*Family_XIII_AD3011_group*	0.72^b^	1.25^a^	0.50^c^	0.091	0.004
	*Family_XIII_UCG-002*	0.12^a^	0.13^a^	0.08^b^	0.007	0.026
	*Unclassified_o_Clostridiales*	0.70^b^	1.07^a^	0.60^b^	0.058	0.01
	*[Eubacterium]_coprostanoligenes_group*	0.63^b^	0.91^a^	0.50^b^	0.05	<0.001
	*[Eubacterium]_nodatum_group*	0.19^ab^	0.24^a^	0.14^b^	0.011	0.021
	*[Eubacterium]_oxidoreducens_group*	0.11^b^	0.19^a^	0.11^b^	0.011	0.044
	*Lachnospiraceae_ND3007_group*	0.56^b^	1.12^a^	0.68^b^	0.07	0.025
	*Unclassified_f_Lachnospiraceae*	0.36^b^	0.78^a^	0.36^b^	0.057	0.014
Proteobacteria	*Ruminobacter*	1.90^a^	0.16^b^	1.14^a^	0.206	<0.001
Saccharibacteria	*Candidatus_Saccharimonas*	0.75^b^	1.16^a^	1.01^ab^	0.048	0.037
Archaea						
Euryarchaeota	*Methanobrevibacter*	78.3^a^	57.6^b^	74.0^a^	3.382	0.014
	*Candidatus_Methanomethylophilus*	3.68^b^	11.42^a^	4.40^b^	1.067	0.001
	*Halostagnicola*	0.04	0.02	0.04	0.003	0.076

Diets: C, corn and corn silage diet; L, sugar beet pulp and alfalfa silage diet; S, steam-flaked corn and corn silage diet. SEM, standard error of the mean. SEM, standard error of the mean. ^a,b,c^ means within a row with different superscripts differ significantly (*p* ≤ 0.05).

In terms of the archaea community, the Euryarchaeota was the most predominant phyla. At the genus level, five archaeal genera were identified from all samples (Supplementary Table S3), and the affected ones with *p* ≤ 0.1 by treatments are shown in Table 5. Compared to the L diet, both C and S diets leaded to a significantly higher relative abundance of *Methanobrevibacter* (*p* = 0.014) but a lower relative abundance of *Candidatus_Methanomethylophilus* (*p* = 0.001) and tended to lead to a higher relative abundance of *Halostagnicola* (*p* = 0.076).

### Predicted metagenomic functions of the ruminal bacteria

**Figure 2:**
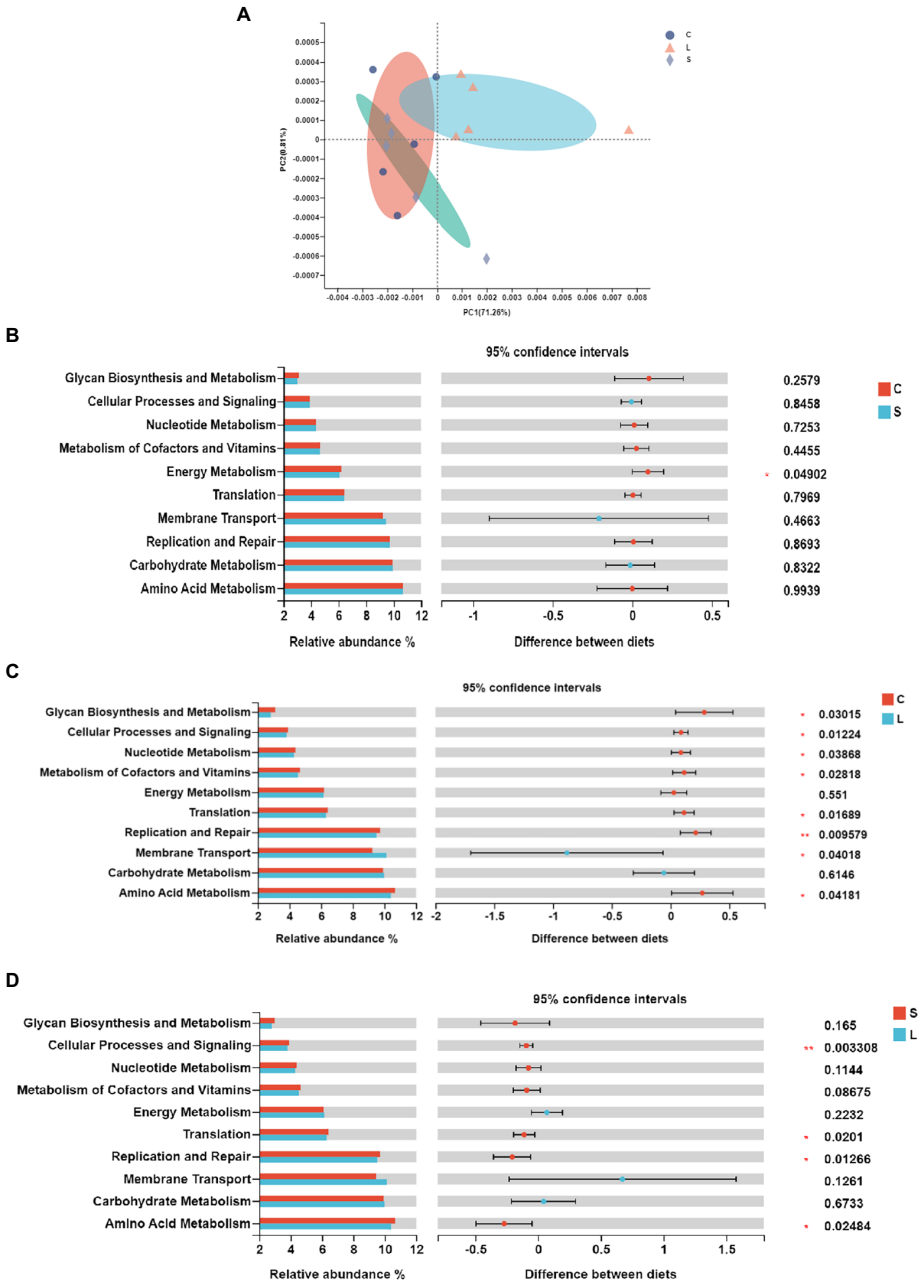
Principal coordinate analysis **(A)** and pairwise comparison **(B–D)** of the KEGG pathways of the bacteria in the rumen fluid of dairy cows after 48 h *in vitro* fermentation with two glucogenic (C, S) and a lipogenic (L) diet. **(B)** C vs. S; **(C)** C vs. L; and **(D)** S vs. L. Diets: C, corn and corn silage diet; L, sugar beet pulp and alfalfa silage diet; and S, steam-flaked corn and corn silage diet.

The functional prediction was conducted with PICRUSt in order to further understand the functioning of ruminal bacteria. Forty functional pathways (level 2) were predicted out of all samples (Supplementary Table S4), with amino acid metabolism, carbohydrate metabolism, and membrane transport being the top three functions. The PCoA analysis showed that samples from the L treatment clustered differently from those in the C and S diets (Figure 2A). The differences of the top 15 most abundant functions among treatments are presented in Figure 2. Compared to the C diet, the S dietary treatment resulted in a higher (*p* = 0.044) relative abundance in energy metabolism (Figure 2B), while the L diet leaded to a higher (*p* = 0.045) relative abundance of membrane transport functions but lower relative abundances in amino acid metabolism (*p* = 0.027), replication and repair (*p* = 0.01), translation (*p* = 0.015), metabolisms of cofactors and vitamins (*p* = 0.025), nucleotide metabolism (*p* = 0.034), and cellular processes and signaling (*p* = 0.003; Figure 2C). Compared to diet L, the S diet leaded to higher relative abundances in amino acid metabolism (*p* = 0.022), translation (*p* = 0.018), replication and repair (*p* = 0.01), and cellular processes and signaling (*p* = 0.003; Figure 2D).

### Rumen metabolomics profiling

**Figure 3:**
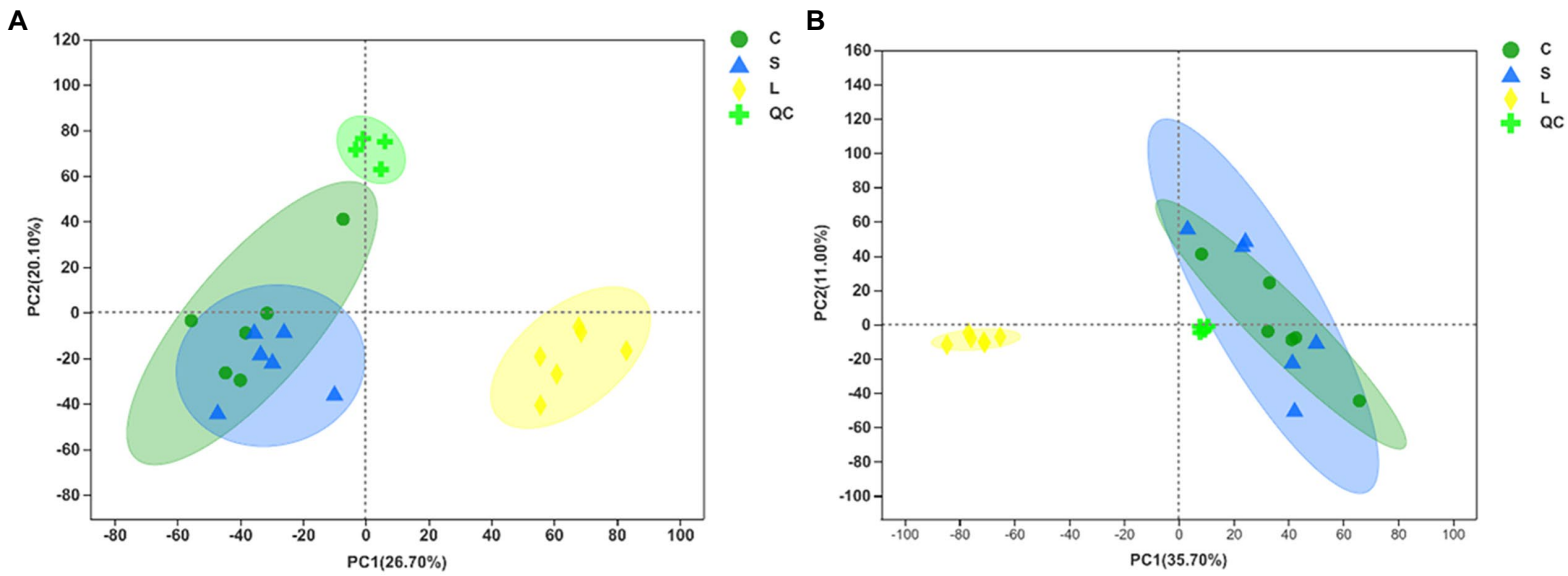
Principal component analysis (PCA) of metabolites following positive **(A)** and negative **(B)** mode ionization based on metabolomics analysis in the ruminal fluid samples of dairy cows after 48 h *in vitro* fermentation with two glucogenic and a lipogenic diet. Diets: C, corn and corn silage diet; L, sugar beet pulp and alfalfa silage diet; S, steam-flaked corn and corn silage diet. QC, quality control samples.

The total ion chromatogram of the QC samples in the positive and negative ion modes indicates the highly reliable repeatability and precision of the data obtained in this analysis (Supplementary Figure S3). Metabolomic data were first examined by PCA in both positive and negative ion mode to obtain an overview of the differences among treatments (Figures 3A,B). The results showed that the metabolites in the rumen fluid fermented with diet L could be separated from those with the other diets. The OPLS-DA score plots indicated a clear separation and discrimination of metabolites between treatments under both positive and negative ion modes (Supplementary Figures S4 A,C,E, S5A,C,E). Next, the response permutation test was to assess the OPLS-DA model in distinguishing the metabolite data between two treatments, in which the cumulative values of *R^2^Y* in the positive (0.9880, 0.8027, and 0.8598 for C vs. S, C vs. L, and L vs. S, respectively, Supplementary Figures S4 B,D,F) and negative (0.9856, 0.8697, and 0.8361 for C vs. S, C vs. L, and L vs. S, respectively, Supplementary Figures S5 B,D,F) ion models were all above 0.80 indicating the stability and reliability of the model.

A total of 801 metabolites (460 in positive ion mode and 341 in negative ion mode) were identified from all rumen fluid samples fermented with three diets, containing 50.3% of the lipids and lipid-like molecules, 13.9% of the organoheterocyclic compounds, 10.9% of the organic acids and derivatives, 9.7% of organic oxygen compounds, 6.4% of both the benzenoids and the phenylpropanoids, and polyketides in the superclass level of the HMDB classification (Supplementary Figure S6).

**Figure 4:**
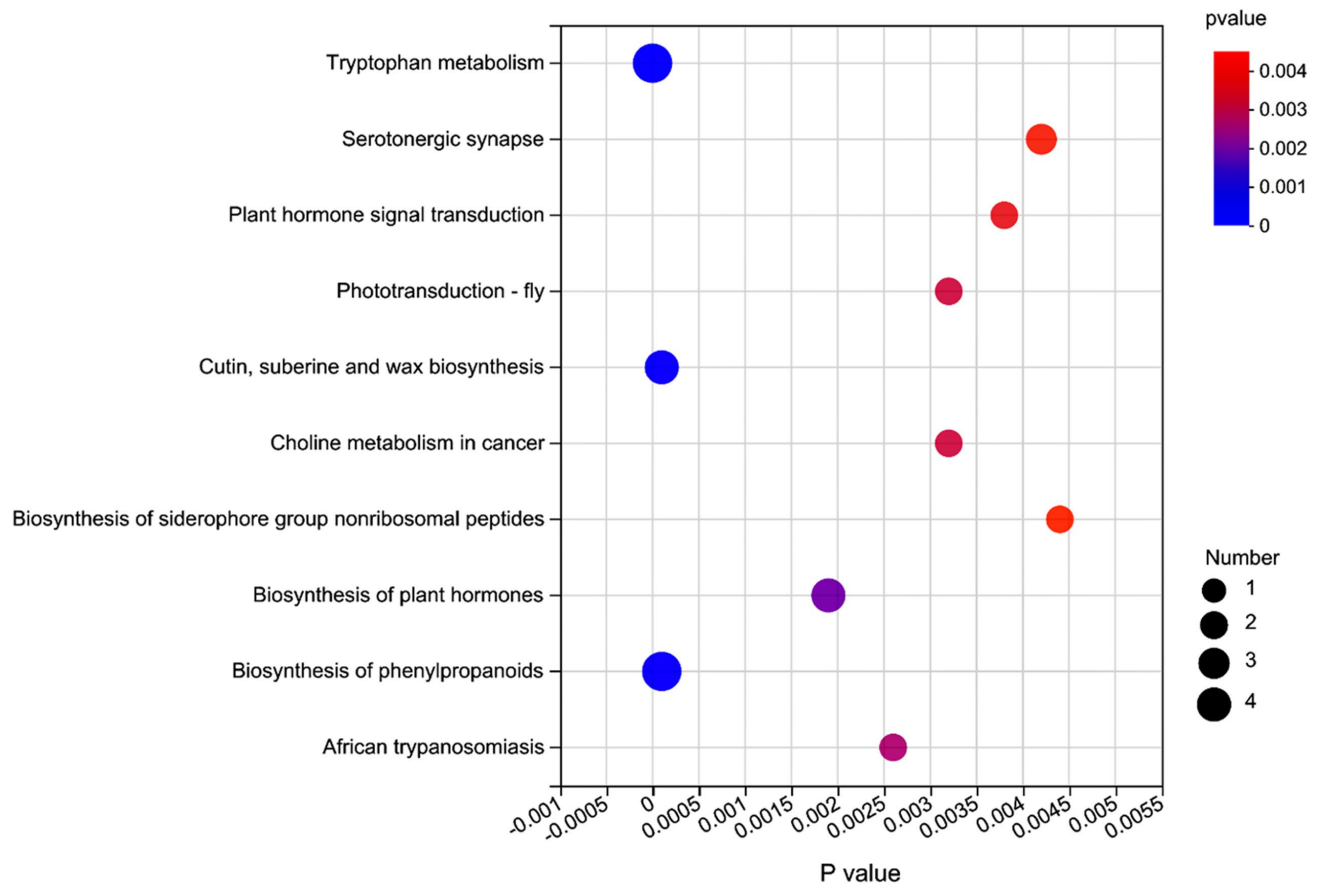
Metabolic pathway enrichment analysis of significant differential metabolites in the rumen fluid of dairy cows after 48 h *in vitro* fermentation with two glucogenic and a lipogenic diet. The color is to distinguish the enrichment significance (*p* value), the darker the color, the more significantly the metabolic pathway is enriched. The *y* axis indicates the name of the KEGG metabolic pathway (top 10). The *x* axis indicates the *p* value. A larger size dot indicates a higher pathway enrichment.

Supplementary Table S5 shows that based on VIP > 1 and *p* ≤ 0.05, a total of 272 significantly affected metabolites (168 positively and 104 negatively ionized metabolites) were obtained from the comparison of L vs. C. Among these metabolites, 20 were classified as the fatty acids and conjugates, 20 as the amino acids, peptides, and analogues, 19 as the triterpenoids, and 14 as the carbohydrates and carbohydrate conjugates. From the comparison of L vs. S, 260 significantly affected metabolites (157 positively and 103 negatively ionized metabolites) were identified (Supplementary Table S6), among which 20 metabolites were classified as the fatty acids and conjugates, 20 as the amino acids, peptides, and analogues, 18 as the triterpenoids, and 14 as the carbohydrates and carbohydrate conjugates. As for the comparison of C vs. S (Supplementary Table S7), 89 significantly affected metabolites (63 positively and 26 negatively ionized metabolites) were detected, of which 12 metabolites were classified as the amino acids, peptides, and analogues, six as the fatty acids and conjugates, and 6 as the carbohydrates and carbohydrate conjugates. The significantly enriched metabolic pathways containing these affected metabolites are shown in Figure 4.

### Correlation between bacteria and the fermentation parameters

**Figure 5:**
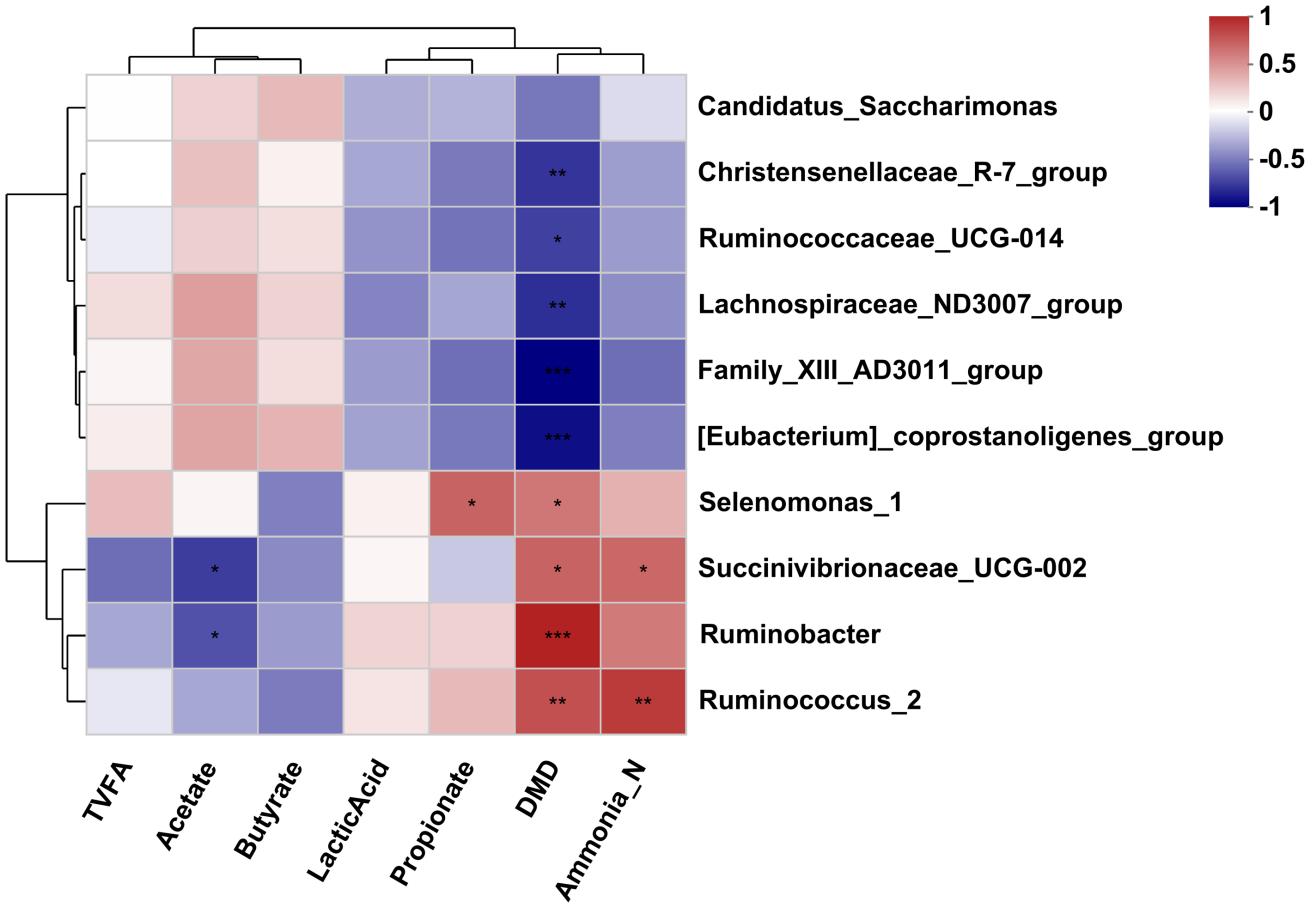
Correlation analysis between differential bacteria genus and differential fermentation parameters in the rumen fluid of dairy cows after 48 h *in vitro* fermentation with two glucogenic and a lipogenic diet. Each row represents a bacteria genus, only the genera with a relative abundance >0.5% are selected; each column represents a fermentation parameter. The color blue means negative correlation, and the color red means positive correlation. ^*^0.01 < *p* ≤ 0.05, ^**^0.001 < *p* ≤ 0.01, ^***^p ≤ 0.001.

The Pearson correlation analysis was conducted to assess the correlation between the affected bacteria and the rumen fermentation parameters. As shown in Figure 5, the DMD was negatively correlated with the *Christensenellaceae_R-7_group*, *[Eubacterium]_coprostanoligenes_group*, *Ruminococcaceae_UCG-014*, *Family_XIII-AD3011_group* and *Lachnospiraceae_ND3007_group* but positively correlated with the *Selenomonas_1*, *Ruminobacter*, *Succinivibrionaceae_UCG-002*, and *Ruminococcus_2*. The NH_3_-N concentration was positively correlated with the *Succinivibrionaceae_UCG-002* and *Ruminococcus_2*. The acetate concentration was negatively correlated with the *Succinivibrionaceae_UCG-002* and *Ruminobacter*. In addition, the propionate concentration was positively correlated with the *Selenomonas_1*.

### Correlation between affected bacteria and the affected metabolites

**Figure 6:**
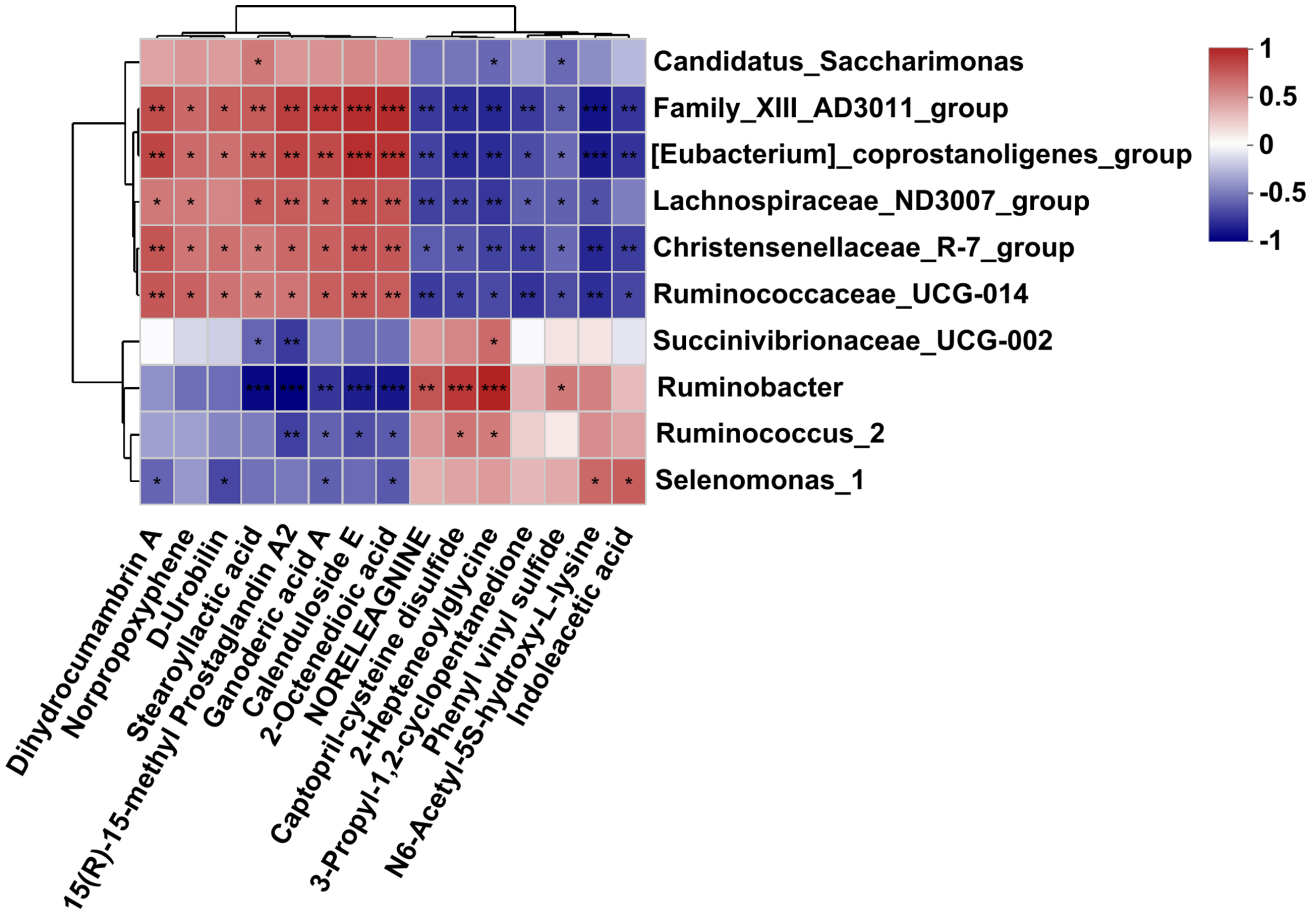
Correlation analysis of differential bacteria genus and differential metabolites in the rumen fluid of dairy cows after 48 h *in vitro* fermentation with two glucogenic and a lipogenic diet. Each row represents a bacteria genus, only the genera with relative abundance >0.5% are selected; each column represents a metabolite, only the affected metabolites with VIP > 1.5, FC < 0.5 and > 2 are considered. The color blue means negative correlation, the color red means positive correlation. ^*^0.01 < *p* ≤ 0.05, ^**^0.001 < *p* ≤ 0.01, ^***^p ≤ 0.001.

As is shown in Figure 6, differently abundant bacterial genera were closely correlated with the affected metabolites in the fermentation fluid. Specifically, the *Family_XIII-AD3011_group*, *[Eubacterium]_coprostanoligenes_group*, and *Christensenellaceae_R-7_group* and *Ruminococcaceae_UCG-010* were positively correlated to the dihydrocumambrin A, norpropoxyphene, D-rrobilin, stearoyllactic acid, 15(R)-15-methyl prostaglandin A2, ganoderic acid A, calenduloside E, and 2-octenedioic acid, but negatively correlated with the noreleagnine, captopril-cysteine disulfide, 2-hepteneoylglycine, 3-propyl-1,2-cyclopentanedione, phenyl vinyl sulfide, N6-acetyl-5S-hydroxy-L-lysine, and indoleacetic acid. Similarly, the *Lachnospiraceae_ND3007_group* was positively correlated to the dihydrocumambrin A, norpropoxyphene, stearoyllactic acid, 15(R)-15-methyl prostaglandin A2, ganoderic acid A, calenduloside E, and 2-octenedioic acid, but negatively correlated with the noreleagnine, captopril-cysteine disulfide, 2-hepteneoylglycine, 3-propyl-1,2-cyclopentanedione, phenyl vinyl sulfide, and N6-acetyl-5S-hydroxy-L-lysine. In addition, *Ruminobacter* was negatively correlated with stearoyllactic acid, 15(R)-15-methyl prostaglandin A2, ganoderic acid A, calenduloside E, and 2-octenedioic acid, but positively correlated with noreleagnine, Captopril-cysteine disulfide, 2-hepteneoylglycine, and phenyl vinyl sulfide. The *Succinivibrionaceae_UCG-002* was negatively correlated with the stearoyllactic acid and 15(R)-15-methyl prostaglandin A2, but positively correlated with 2-hepteneoylglycine. *Ruminococcus_2* was negatively correlated with the 15(R)-15-methyl prostaglandin A2, ganoderic acid A, calenduloside E, and 2-octenedioic acid, but positively correlated with the captopril-cysteine disulfide and 2-hepteneoylglycine. The *Selenomonas_1* was negatively correlated with the dihydrocumambrin A, D-urobilin, ganoderic acid A, and 2-octenedioic acid, but positively correlated with the N6-acetyl-5S-hydroxy-L-lysine and indoleacetic acid. Moreover, *Candidatus_saccharimonas* was positively correlated with the stearoyllactic acid but negatively correlated with the 2-hepteneoylglycine and phenyl vinyl sulfide.

## Discussion

The present research reports the influence of two glucogenic diets and a lipogenic diet on ruminal fermentation end-products using an *in vitro* incubation system and provides the unknown information on metabolites formed and the bacterial communities in response to the glucogenic and lipogenic diets.

### Influence on feed digestion and gas production

The L diet had a lower DMD than the other two diets, which is consistent with previous studies (Ruppert et al., 2003). The ruminal bacteria can be assigned to different functional groups, such as cellulolytic, amylolytic, and proteolytic, based on their preferential use of energy. Starch digestion in the rumen is affected by dietary starch source, grain processing, and adhering capacity of ruminal microbiota to the diet particles (Huntington, 1997). The main amylolytic bacteria included *Streptococcus bovis*, *Bacteroides amylophilus*, *Prevotella* spp., *Succinimonas amylolytica*, *Selenomonas ruminantium*, and *Butyrivibrio* spp. (Xia et al., 2015). For the present study, the relative abundance of amylolytic bacteria genera, including *Selenomonas_1*, *Ruminobacter*, and *Succinivibrionaceae_UCG-002*, were higher in diet C and S compared to diet L and also were significantly positively correlated with DMD. These increased genera may likely have contributed to the higher DMD in diets C and S.

The fiber degradation in the rumen is mainly attributed to the ruminal cellulolytic bacteria (Jeyanathan et al., 2014). *Fibrobacter succinogenes* (belong to the genus *Fibrobacter*), *Ruminococcus flavefaciens*, and *Ruminococcus albus* (belong to the genus *Ruminococcus*) were considered the dominant cellulolytic bacterial species due to their high capacity for cellulose digesting (Krause et al., 2003). In addition, the genera of *Butyrivibrio* and *Eubacterium* were also reported to be cellulolytic (Thoetkiattikul et al., 2013). Moreover, some unclassified taxa, including those assigned to *Ruminococcaceae*, *Lachnospiraceae*, *Christensenellaceae*, *Rikenellaceae*, *Prevotellaceae*, *Clostridium*, and *Bacteroidales* were proven to have the capacity of adhering tightly to forages in the rumen, which indicates that these new taxa might play a significant role in forage digestion (Liu et al., 2016). In this study, there were significantly higher relative abundances of the cellulolytic bacterial genera in the rumen fluid fermented with diet L, including *Ruminococcus*, *Eubacterium*, *Butyrivibrio_2*, and *Lachnospira*, and the potential cellulolytic taxa, including unclassified*_Lachnospiraceae*, unclassified*_Ruminococcaceae*, and unclassified*_o_Clostridiales*. A previous study indicated that the digestibility of cellulose was lower when high corn silage was fed to dairy cows compared to high alfalfa silage (Ruppert et al., 2003). This could explain the higher cellulolytic bacteria in diet L. These bacteria were proved to be more sensitive to lipogenic nutrients in diet.

In the present study, the gas production of the ruminant feeds was highly correlated with their digestibility and available energetic contents, the higher DMD of two glucogenic diets was the reason for their higher gas production than diet L, which agree with the early work of Menke and Steingass (1988). The steam-flaked corn, compared to ground corn, increased the gas production of the total mixed rations (TMR) incubated with buffered rumen liquor *in vitro* and increased the gas production rate, which agrees with the data of Qiao et al. (2015). The processing conditions (increased moisture content, pressure, and temperature) involved in producing steam-flaking corn have been shown to improve the enzymatic hydrolysis of starch *in vitro*, thereby improving the digestibility of corn (de Peters et al., 2003). Starch digestibility was shown to be positively related to the percentage of starch that was gelatinized *in vitro* (Huntington, 1997). The gelatinization of the starch in the steam-flaking corn was highly likely the reason for their higher gas production.

Methanogenesis is an ancient metabolism of the methanogens belonging to the phylum *Euryarcheota*, domain archaea (Hook et al., 2010). All methanogens belong to seven euryarchaeal orders, including *Methanococcales*, *Methanopyrales*, *Methanobacteriales*, *Methanosarcinales*, *Methanomicrobiales*, *Methanocellales*, and *Thermoplasmatales* (Hook et al., 2010). Three classical CH_4_-producing pathways were reported previously, including the hydrogenotrophic methanogenesis mainly using CO_2_/H_2_ or formate as substrate, acetoclastic methanogenesis with acetate as substrate and methylotrophic methanogenesis with methylated C1 compounds as substrate (Hedderich and Whitman, 2006). Methanogens are known to grow better syntrophically *in vitro* (Sakai et al., 2009). For ruminants, *Methanobrevibacter* was recognized as the dominant genus producing CH_4_ (Leahy et al., 2013), mainly through the CO_2_/H_2_ pathway using CO_2_ or formate as the elector acceptor and H_2_ as the electron donor (Liu and Whitman, 2008). Our results are in line with the aforementioned observations and illustrate that the relative abundance of the dominant genus *Methanobrevibacter* followed the same trend as gas production and CH_4_ proportion. The higher gas production of the S relative to the L diet might supply more substrates (CO_2_/H_2_) for the methanogenesis of *Methanobrevibacter*, which may lead to higher CH_4_ production. The genus of *Candidatus_Methanomethylophilus* is also known as a CH_4_-producing methanogen, which mainly depends on methanol as substrate *via* the methylotrophic methanogenesis pathway (Lino et al., 2013). In the present study, the L diet increased the genus of *Candidatus_Methanomethylophilus* significantly. Since this genus was newly defined, its methanogenesis pathway and its relationship with dietary ingredients deserve further research.

### Influence on lactic acid and NH_3_-N

High starch concentration would decrease rumen pH (Nocek et al., 2002). The present study found that lactic acid concentration was negatively related to the pH value. The low pH value of the C and S diet is likely mainly attributed to their increase in lactic acid production. In addition, no difference between the S and C diets existed in the pH value, which is in line with the previous study (Cooper et al., 2002).

The NH_3_-N concentration in the *in vitro* rumen fluid cultures is determined by the balance of the formation and utilization rate of NH_3_-N by microorganisms (Shen et al., 2017). Due to the same level of crude protein among all treatments, the supplemented amount of nitrogen available for the microbiota can be considered equal. The efficiency of ruminal NH_3_-N utilization is determined by the capacities of microbes to metabolize NH_3_-N. Cellulolytic bacteria which degrade structural carbohydrates (e.g., NDF) grow slowly and mainly use NH_3_-N as an N source, whereas amylolytic bacteria which degrade non-structural carbohydrates (e.g., starch) grow rapidly with higher requirements and use ammonia, peptides, and amino acid as N sources (Fox et al., 1992). In our study, the NH_3_-N concentration in diet L was significantly lower than in the other two diets. The L diet showed higher relative abundances of cellulolytic bacteria, among which the genera *Succinivibrionaceae_UCG-002* and *Ruminococcus_2* was proved to positively correlate with the NH_3_-N concentration. These bacteria might use NH_3_-N as the main N source leading to a lower NH_3_-N concentration.

### Influence on VFA

Ruminal fermentation of carbohydrates leads to the formation of VFA. The primary ruminal VFAs are acetate, propionate, and butyrate, the molar proportions of which are mainly determined by the diet. Propionate and acetate are the main precursors of milk glucose and fatty acids, respectively. Cellulose ferments to acetate to a greater extent than propionate, whereas readily degradable starch is fermented less to acetate and more to propionate. Consistent with this, our data showed that both the C and S diets had a higher concentration of propionate and a lower acetate to propionate ratio compared to the L diet. The ruminal propionate is formed *via* two main pathways, the succinate pathway and the acrylate pathway (Jeyanathan et al., 2014). The former is known as the major pathway for propionate-production in the rumen and involves a large number of bacterial species, such as bacteria related to succinate production and utilization (*Fibrobacter succinogenes* and *Selenomonas ruminantium*), and lactate production and utilization (e.g., *Streptococcus Bovis* and *Selenomonas ruminantium*; Jeyanathan et al., 2014). The genus *Selenomonas_1* had a positive correlation with the propionate concentration, and the relative abundance of *Selenomonas_1* and *Succinivibrionaceae_UCG-002* in the L diet was lower than that in the other diets. The *Selenomonas_1* and *Succinivibrionaceae_UCG-002* may contribute to the higher propionate production in diets C and S by enhancing the succinate pathway according to current knowledge. These roles need to be confirmed by further research.

### Influence on bacterial function

To further study the differences in the functional roles of rumen bacteria among dietary treatments, PICRUSt was used to estimate the potential functions of the bacteria. Compared with the C and S diets, the predicted pathway of amino acid metabolism had a lower level in the L dietary treatment (Figures 2C,D). The increased amino acid metabolism in diets S and C may lead to higher amino acid production with excessive amounts of amino acids contributing to the higher NH_3_-N concentration *via* deamination (Petri et al., 2019). Also, the *Ruminobacter amylophilus* is known for its proteolytic activity (Amlan and Ye, 2014), which could explain that the diets C and S with a higher number of the genus *Ruminobacter* had enhanced function of amino acids metabolism. Moreover, the L diet reduced the relative abundance of the translation, replication and repair, as well as cellular processes and signaling, which is probably attributed to the rapid turnover rate of bacteria (Zhang et al., 2017a). As predicted by PICRUSt, the bacteria in the C diet had an enriched function for energy metabolism compared to the S diet, suggesting that the bacterial capacity of energy intake may be improved by the ground corn compared to the steam-flaked corn.

### Influence on rumen metabolites

The metabolomics analysis provides direct evidence for changes in microbial activities among diets. The metabolomics data showed that the dietary treatments altered most metabolites related to lipid and protein digestion. The enriched metabolic pathways that were predicted by PICRUSt, such as amino acid metabolism and cellular processes and signaling, were similar to the enriched metabolic pathways through the metabolome functions analysis, such as the tryptophan metabolism and sphingolipid signaling pathways.

Most affected metabolites in the lipids and lipid-like molecules were higher in the diet L compared to the other two diets, indicating that the L diet could promote lipid utilization to some degree. Most metabolites belonging to fatty acids and conjugates were also higher in the diet L. Previous *in vitro* bacterial culturing experiments have shown that fatty acids had a negative effect on bacterial growth (Henderson, 1973). The bacterial communities were modified differently by the fatty acid supplements, where cellulolytic strains of bacteria showed to be more sensitive to fatty acids than the amylolytic ones (Doreau and Ferlay, 1995). The present contribution also observed a strong correlation between the cellulolytic bacteria and metabolites associated with fatty acid. The initial step of lipid metabolism in the rumen is the hydrolysis of the ester linkages, which is predominantly controlled by rumen bacteria (Bauman et al., 2003). The strains of *Butyrivibrio fibrisolvens* have been reported to play an important role in the degradation of polyunsaturated fatty acids in the rumen (Latham et al., 1972), including hydrolysing phospholipids and glycolipids (Harfoot and Hazlewood, 1997). Besides, some strains of *Borrelia* (Yokoyama and Davis, 1971), a strain in each of *Ruminocccus* and *Eubacterium* (Kemp et al., 1975) and two strains of cellulolytic *Clostridium* spp. (Viviani et al., 1968) have also been reported to participate in biohydrogenation. The higher abundance of genera *Butyrivibrio_2*, *Ruminococcus_1*, *Ruminococcaceae_UCG-013*, *Ruminococcaceae_UCG-014*, and *Unclassified_o_Clostridiales* in diet L is in line with the higher level of the metabolites related to fatty acids and conjugates. Correlation analysis proved that the different fatty acid metabolites had significant relations with several cellulolytic bacteria, including the *Ruminococcaceae_UCG-014* and *[Eubacterium]_coprostanoligenes_group*. These cellulolytic bacteria might contribute to the higher fatty acid production in the L diet.

Most metabolites associated with the amino acids, peptides, and analogues were decreased in the L compared to the C and S diets, which was also in line with the PICRUSt result. The ruminal amino acids mainly arise from the dietary protein degradation and protein produced by microbiota. A large number of microbial species contribute to the ruminal proteolysis, with starch-degrading bacteria contributing more to the protein degradation in the rumen than the cellulolytic bacteria (Zhang et al., 2017a). This could explain the high level of amino acids, peptides, and analogues in the C and S diets. Besides, in the *de novo* synthesis of ruminal amino acids, acetate and propionate can be used as carbon sources and the compounds like ammonia as the nitrogen sources by the microbes (Zhang et al., 2017b). The high concentrations of propionate, butyrate and NH_3_-N in diet C and S also agrees with their higher levels of amino acids.

In conclusion, the glucogenic diet had greater effects than the lipogenic diet in terms of improving the dry matter digestibility, increasing propionate concentration and promoting amino acid metabolism. The improvement in propionate production may be attributed to the increased number of bacterial spp. functioning in the succinate pathway. Compared to ground corn, steam-flaked corn did not show more differences in fermentation end-products except for increasing gas production and lower production of some fatty acids and amino acids. Several amylolytic and cellulolytic bacteria were observed to be sensitive to the dietary changes, while most highly abundant bacteria were stable or minorly affected. The affected bacteria showed to have high associations with certain metabolites. This study has offered a deeper understanding of ruminal microbial functions which could assist the improvement of rumen functions and thereby in the ruminant production. Moreover, these findings provide essential references for future *in vivo* studies.

## Data availability statement

The data presented in the study are deposited in the NCBI repository, accession number PRJNA661353.

## Ethics statement

The animal study was reviewed and approved by the Animal Care and Use Committee of the Chinese Academy of Agricultural Sciences.

## Author contributions

DH, WH, WP, and BX designed the research. DH, YZ, FX, and YW performed the research. WH, WP, and BX supervised the research. LJ supported the equipment for all the testing. DH analyzed the data and wrote the manuscript. WH and WP supervised the manuscript writing. All authors contributed to the article and approved the submitted version.

## Funding

The study was financially supported by the National Key R&D Program of China (2021YFE2000800 and 2019YFE0125600) and the Science and Technology Innovation Project of the Institute of Animal Sciences (cxgc-ias-09).

## Conflict of interest

The authors declare that the research was conducted in the absence of any commercial or financial relationships that could be construed as a potential interest conflict.

## Publisher’s note

All claims expressed in this article are solely those of the authors and do not necessarily represent those of their affiliated organizations, or those of the publisher, the editors and the reviewers. Any product that may be evaluated in this article, or claim that may be made by its manufacturer, is not guaranteed or endorsed by the publisher.

## Footnotes

^1^ https://metlin.scripps.edu/

^2^ https://cloud.majorbio.com
