# Supplementary material for: Glucogenic and lipogenic diets affect in vitro ruminal microbiota and metabolites differently
Source: Front Microbiol. 2022 Dec 16;13:1039217. doi: 10.3389/fmicb.2022.1039217 (PMC9800790; doi:10.3389/fmicb.2022.1039217)
Supplement: Supplementary file 1 [file Data_Sheet_1.zip › Table 1 - 2022-11-25T101340.700.DOCX]

**Supplementary material Figure S1-S5**

| 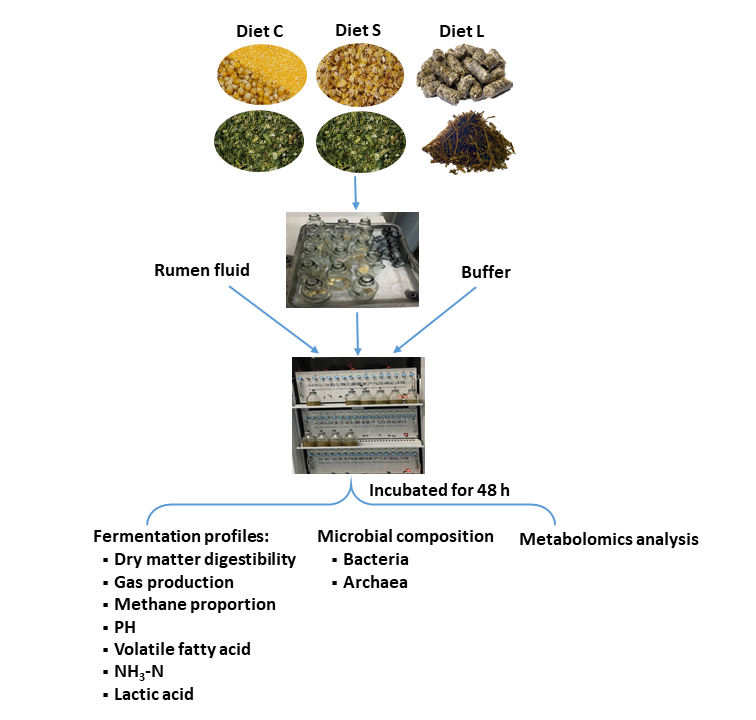 |
| --- |
| **Figure S1. Technical route of the present trial**. Diets: C, corn and corn silage diet; L, sugar beet pulp and alfalfa silage diet; S, steam-flaked corn and corn silage diet. |

| 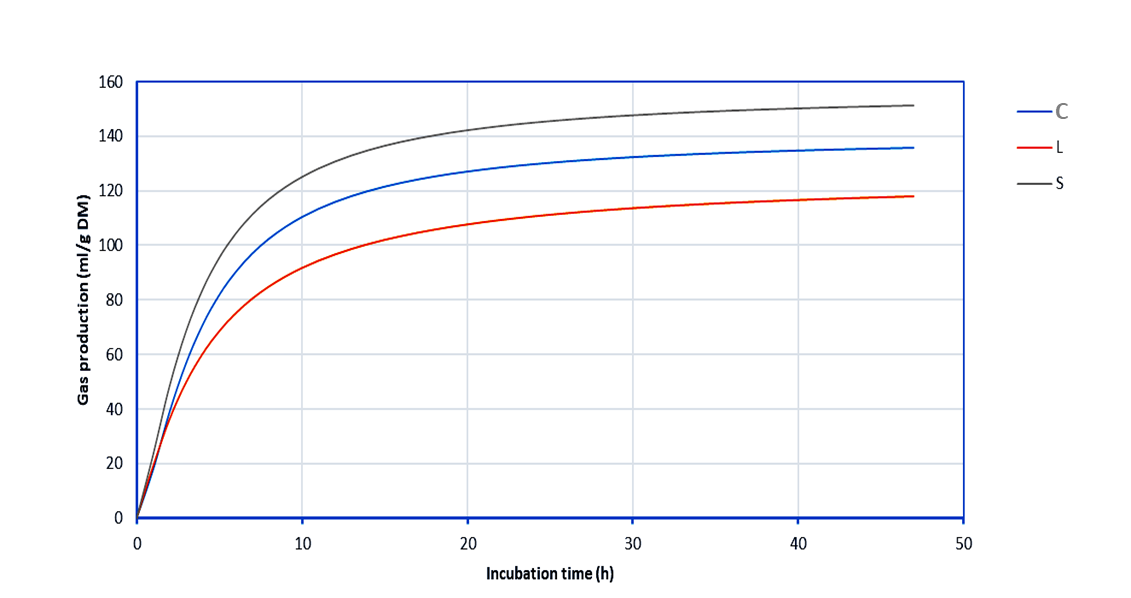 |
| --- |
| **Figure S2. Effects of two glucogenic (C, S) and a lipogenic (L) diet on the gas production curve after 48 h *in vitro* fermentation with rumen fluid of dairy cows**. The cumulative gas production curve was fitted to the monophasic model: GP = A / (1 + (C / t) ^B^), where GP = total gas produced (ml/g DM); A = asymptotic gas production (ml/g DM); B = switching characteristic of the curve; C = time at which half of the asymptote has been reached; t = time (h). Diets: C, corn and corn silage diet; L, sugar beet pulp and alfalfa silage diet; S, steam-flaked corn and corn silage diet. |

| 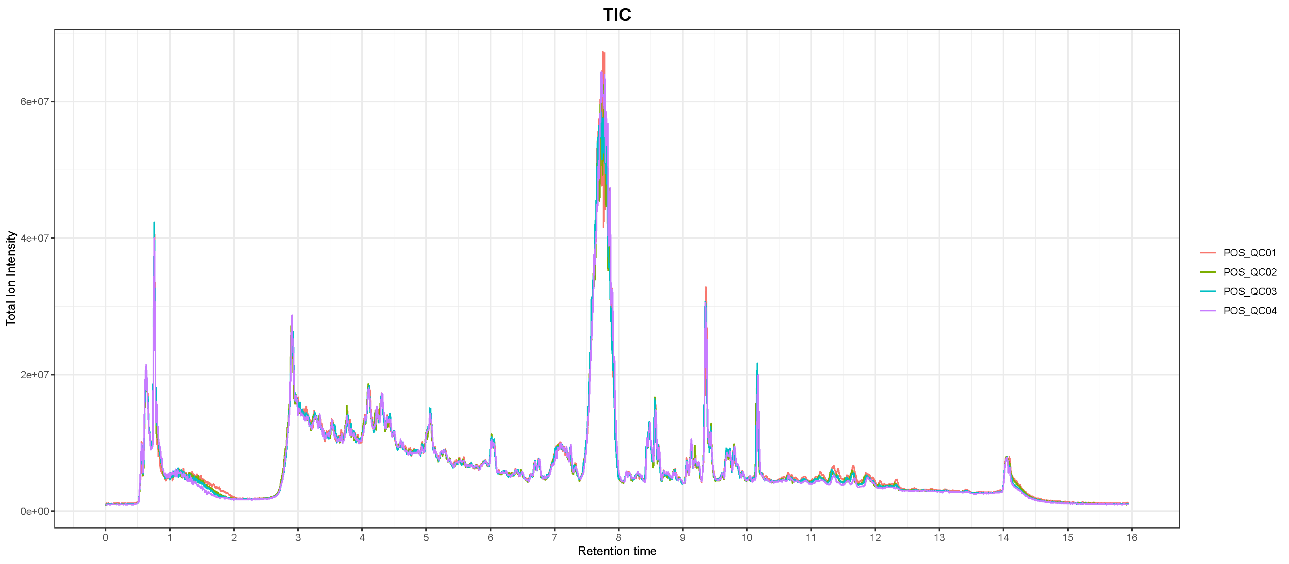a |
| --- |
| 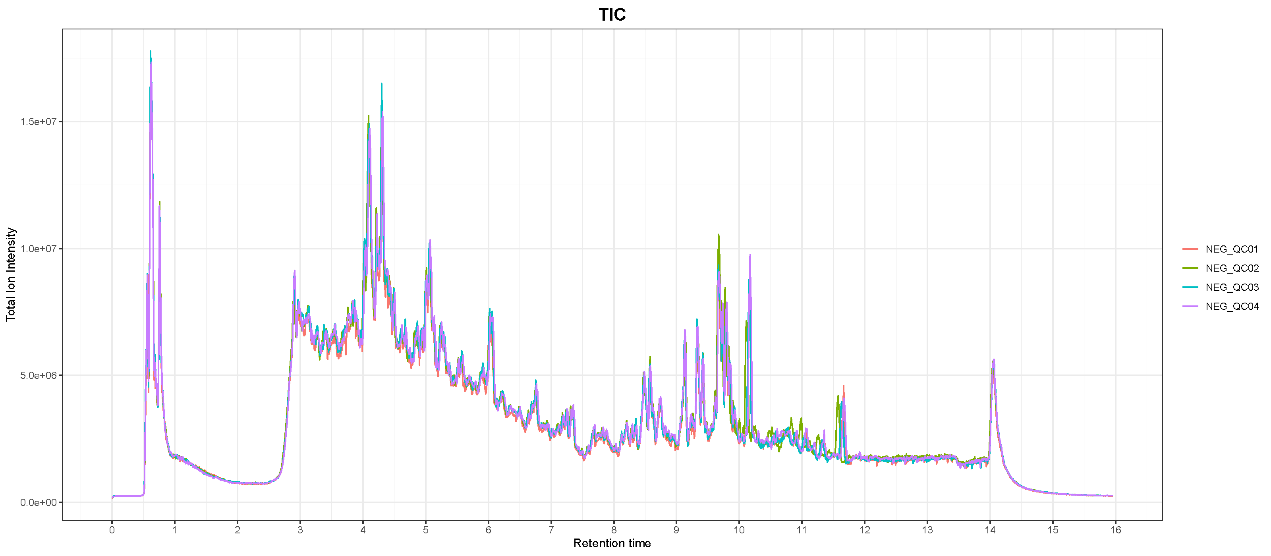b |
| **Figure S3. LC-MS/MS total ion chromatogram (TIC) of the QC samples in (a) the positive ion mode and (b) the negative ion mode. QC, quality control samples** |

| 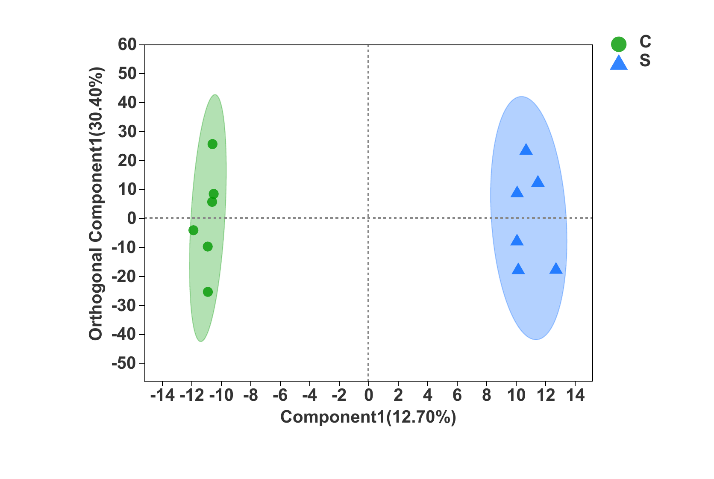**a** | 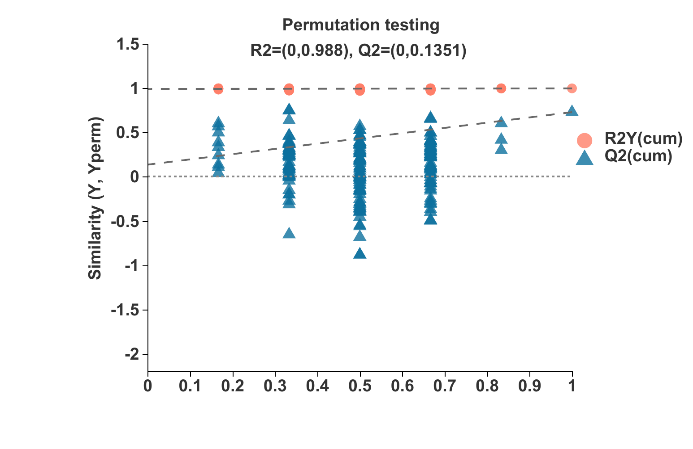**b** |
| --- | --- |
| 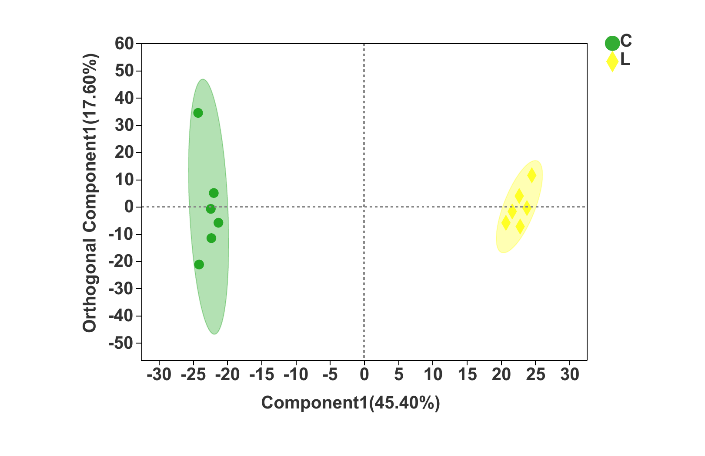**c** | 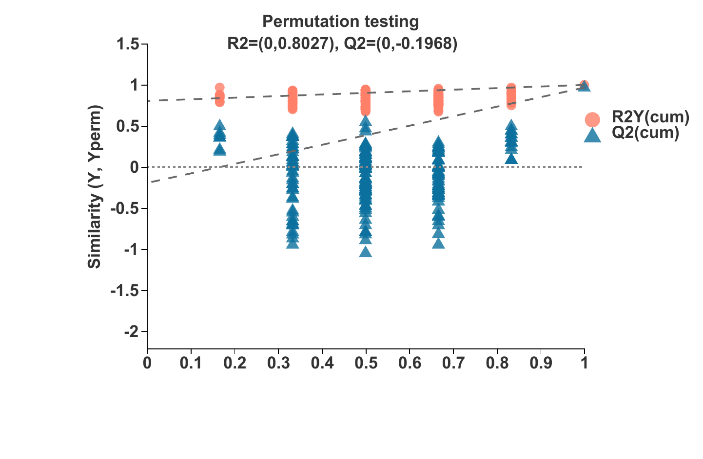**d** |
| 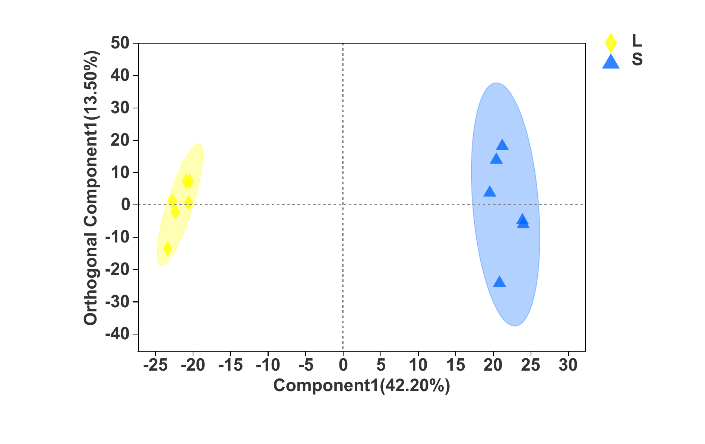**e** | 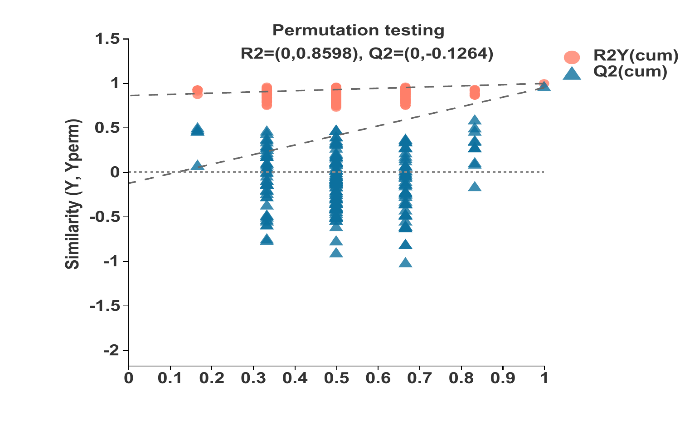**f** |
| **Figure S4. Orthogonal partial least squares discriminant analysis (OPLS-DA) (a, c, e) and corresponding permutation test following positive mode ionization (b, d, f) derived from the metabolomics analysis in the rumen fluid of dairy cows after 48 h *in vitro* fermentation with two glucogenic (C, S) and a lipogenic (L) diet**. Diets: C, corn and corn silage diet; L, sugar beet pulp and alfalfa silage diet; S, steam-flaked corn and corn silage diet. | |

| 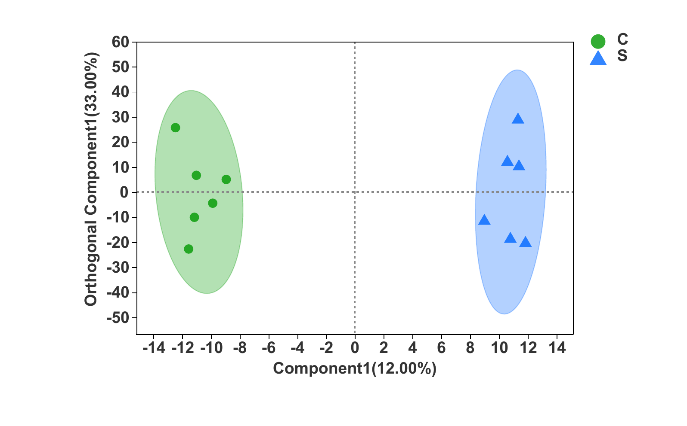**a** | 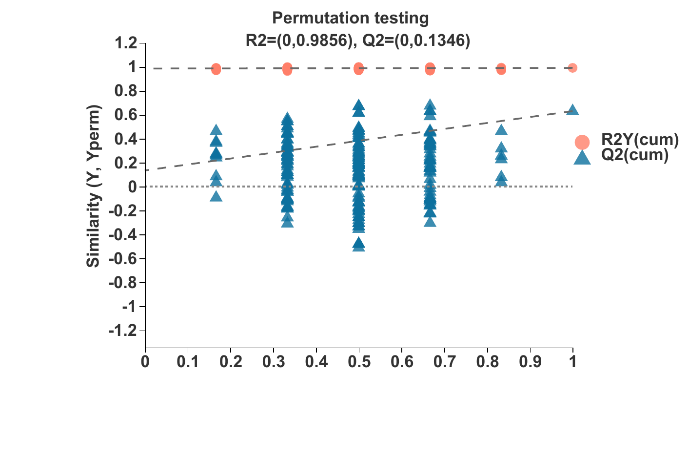**b** |
| --- | --- |
| 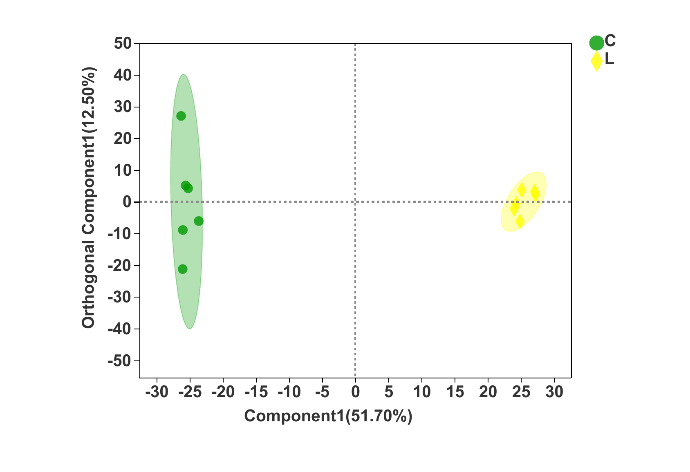**c** | 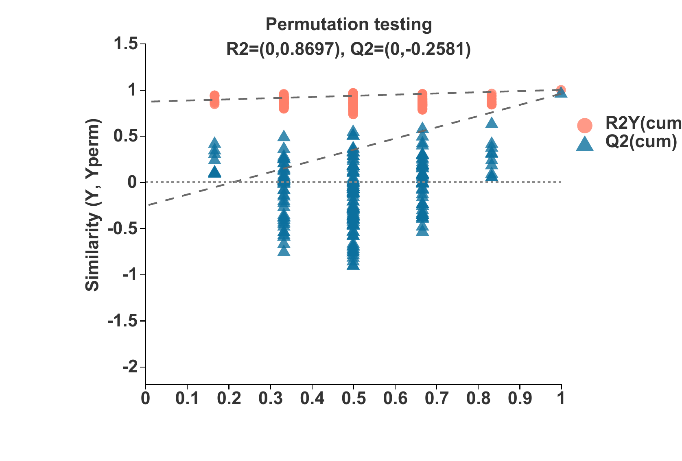**d** |
| 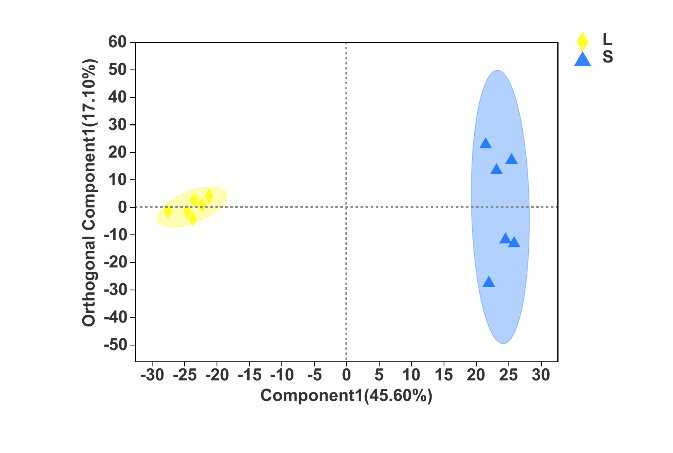**e** | 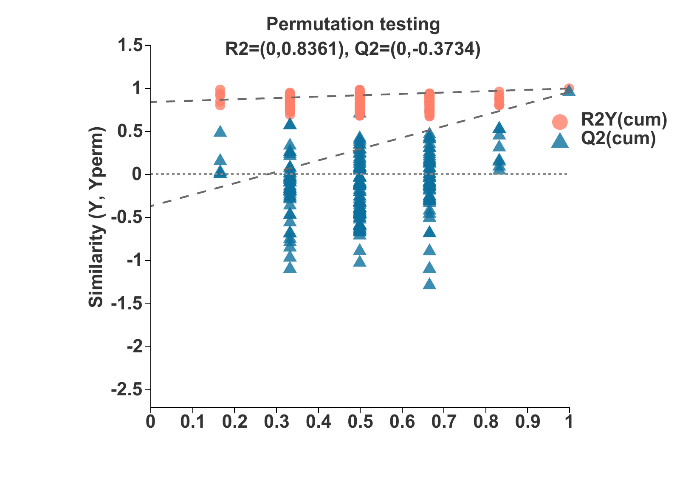**f** |
| **Figure S5. Orthogonal partial least squares discriminant analysis (OPLS-DA) (a, c, e) and corresponding permutation test following negative mode ionization (b, d, f) derived from the metabolomics analysis in the rumen fluid of dairy cows after 48 h *in vitro* fermentation with two glucogenic (C, S) and a lipogenic (L) diet**. Diets: C, corn and corn silage diet; L, sugar beet pulp and alfalfa silage diet; S, steam-flaked corn and corn silage diet. | |

| 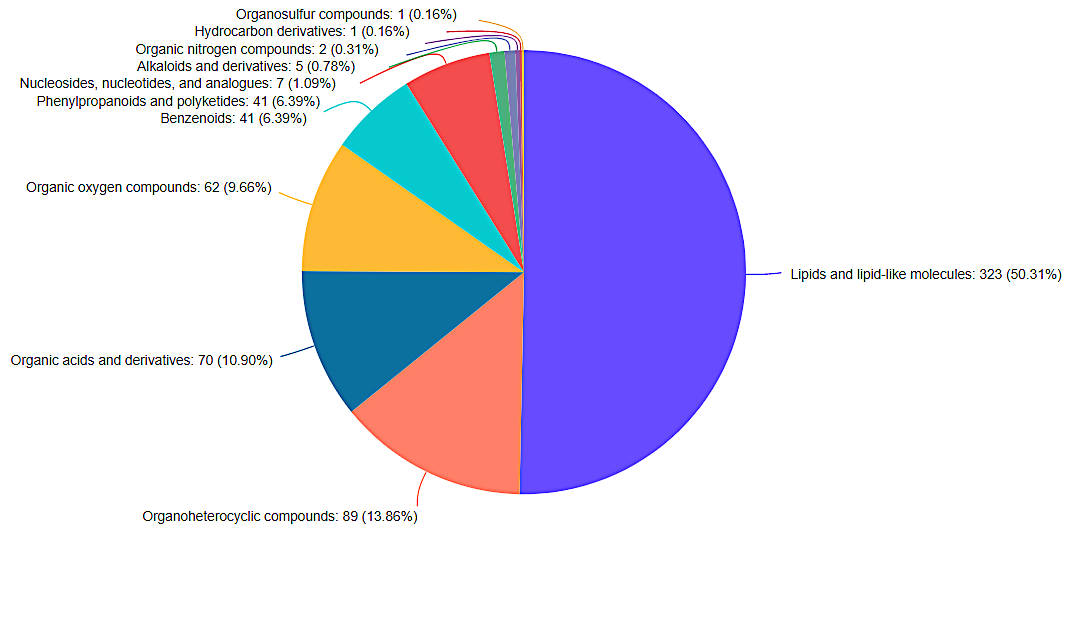 |
| --- |
| **Figure S6. The HMDB compound classification of 801 identified metabolites (in the superclass level) detected by metabolomics in the rumen fluid of dairy cows after 48 h *in vitro* fermentation with two glucogenic and a lipogenic diet**. HMDB, the human metabolome database. |
