# Supplementary material for: Glucogenic and lipogenic diets affect in vitro ruminal microbiota and metabolites differently
Source: Front Microbiol. 2022 Dec 16;13:1039217. doi: 10.3389/fmicb.2022.1039217 (PMC9800790; doi:10.3389/fmicb.2022.1039217)
Supplement: Supplementary file 1 [file Data_Sheet_1.zip › Table 2 - 2022-11-25T100914.668.DOCX]

**Supplementary material and TableS1-S7**

**DNA extraction and amplification**

**For the bacteria,** the primers amplifying the V3-V4 hypervariable regions of the bacterial 16S rRNA gene: 338F (5’-ACTCCTACGGGAGGCAGCAG-3’) and 806R (5'-GGACTACHVGGGTWTCTAAT-3'). PCR reactions were performed in a triplicate 20 μl mixture containing 4 μl of 5 × FastPfu Buffer, 2 μl of 2.5 mM dNTPs, 0.8 μl of each primer (5 μM), 0.4 μl of FastPfu Polymerase, 0.2 μl of BSA, and 10 ng of template DNA. The PCR program contains 3 min of denaturation at 95 °C; 27 cycles of 30 s at 95 °C, 30 s for annealing at 55 °C, 45 s for elongation at 72 °C; a final extension at 72 °C for 10 min.

**For the archaea,** in the first PCR circle, the primers amplifying the V3-V4 hypervariable regions of the archaeal 16S rRNA gene were 340F (CCCTAYGGGGYGCASCAG) and 1000R (GGCCATGCACYWCYTCTC). PCR reactions were performed in a triplicate 30 μl mixture containing 15 μl of 2 × Taq master Mix (P111-03, Vazyme), 1 μl of bar-PCR primer F (10 μM), 1 μl of primer R (10 μM), 10-20 ng of template DNA. The PCR program contains 3 min of denaturation at 94 °C; 5 cycles of 30 s at 94 °C, 20 s for annealing at 45 °C, 30 s for elongation at 65 °C; 20 cycles of 20 s at 94 °C, 20 s for annealing at 55 °C, 30 s for elongation at 72 °C; a final extension at 72 °C for 5 min.

In the second PCR circle, the primers were 349F (5’- CCCTACACGACGCTCTTCCGATCTN (barcode) GYGCASCAGKCGMGAAW -3’) and 806R (5’- GACTGGAGTTCCTTGGCACCCGAGAATTCCAGGACTACVSGGGTATCTAAT-3’).

PCR reactions were performed in a triplicate 30 μl mixture containing 15 μl of 2 × Taq master Mix (P111-03, Vazyme), 1 μl of bar-PCR primer F (10 μM), 1 μl of primer R (10 μM), 10-20 ng of PCR products from the first circle. The PCR program contains 3 min of denaturation at 94 °C; 5 cycles of 30 s at 94 °C, 20 s for annealing at 45 °C, 30 s for elongation at 65 °C; 20 cycles of 20 s at 94 °C, 20 s for annealing at 55 °C, 30 s for elongation at 72 °C; a final extension at 72 °C for 5 min.

| **Table S1. Effect of two glucogenic (C, S) and a lipogenic (L) diet on the relative abundance (%) of ruminal bacterial phyla after 48 h *in vitro* fermentation** | | | | | |
| --- | --- | --- | --- | --- | --- |
| Phylum | Experimental diet | | | SEM | *P*-value |
|  | C | L | S |  |  |
| Bacteroidetes | 49.4 | 45.0 | 48.1 | 1.704 | 0.424 |
| Firmicutes | 36.1 | 41.6 | 39.0 | 2.065 | 0.411 |
| Proteobacteria | 6.54 | 5.16 | 3.87 | 0.777 | 0.181 |
| Verrucomicrobia | 4.08 | 3.25 | 4.36 | 0.444 | 0.638 |
| Spirochaetae | 1.50 | 1.64 | 2.09 | 0.281 | 0.762 |
| Saccharibacteria | 0.81 | 1.16 | 1.01 | 0.085 | 0.092 |
| Synergistetes | 0.41 | 0.54 | 0.33 | 0.050 | 0.312 |
| Lentisphaerae | 0.40 | 0.47 | 0.36 | 0.042 | 0.688 |
| Tenericutes | 0.24^b^ | 0.49^a^ | 0.25^b^ | 0.039 | 0.042 |
| SR1__Absconditabacteria | 0.14 | 0.28 | 0.22 | 0.027 | 0.084 |
| Actinobacteria | 0.14 | 0.14 | 0.14 | 0.014 | 0.996 |
| Only the bacterial phyla that account for ≥ 0.1% in at least one of the samples are listed; Diets: C, corn and corn silage diet; L, sugar beet pulp and alfalfa silage diet; S, steam-flaked corn and corn silage diet. SEM, standard error of the mean.  ^a, b^ means within a row with different superscripts differ significantly (*P* ≤ 0.05). | | | | | |

| **Table S2. Effect of two glucogenic (C, S) and a lipogenic (L) diet on the relative abundance (%) of ruminal bacterial genera after 48 h *in vitro* fermentation with rumen fluid of dairy cows** | | | | | | |
| --- | --- | --- | --- | --- | --- | --- |
| Phylum | Genus/others | Experimental diet | | | SEM | *P*-value |
|  |  | C | L | S |  |  |
| Bacteroidetes | *SP3-e08* | 0.11^b^ | 0.17^a^ | 0.10^b^ | 0.009 | 0.011 |
| Firmicutes | *Christensenellaceae_R-7_group* | 1.28^b^ | 1.70^a^ | 1.06^b^ | 0.077 | 0.029 |
|  | *Ruminococcaceae_UCG-014* | 0.74^b^ | 1.05^a^ | 0.70^b^ | 0.014 | 0.026 |
|  | *Family_XIII_AD3011_group* | 0.72^b^ | 1.25^a^ | 0.50^b^ | 0.091 | 0.004 |
|  | *Ruminococcus_2* | 0.87^a^ | 0.55^b^ | 1.00^a^ | 0.070 | 0.018 |
|  | *Unclassified_o_Clostridiales* | 0.70^b^ | 1.07^a^ | 0.60^b^ | 0.058 | 0.010 |
|  | *Lachnospiraceae_ND3007_group* | 0.56^b^ | 1.12^a^ | 0.68^b^ | 0.070 | 0.025 |
|  | *[Eubacterium]_coprostanoligenes_group* | 0.63^b^ | 0.91^a^ | 0.50^b^ | 0.051 | <0.001 |
|  | *Selenomonas_1* | 0.38^b^ | 0.26^b^ | 0.88^a^ | 0.077 | 0.005 |
|  | unclassified*_f_Lachnospiraceae* | 0.36^b^ | 0.78^a^ | 0.36^b^ | 0.057 | 0.014 |
|  | *Butyrivibrio_2* | 0.24^b^ | 0.62^a^ | 0.29^b^ | 0.076 | 0.037 |
|  | unclassified*_f_Ruminococcaceae* | 0.19^b^ | 0.35^a^ | 0.20^b^ | 0.021 | 0.001 |
|  | *Ruminococcaceae_UCG-013* | 0.17^b^ | 0.35^a^ | 0.15^b^ | 0.026 | 0.006 |
|  | *[Eubacterium]_nodatum_group* | 0.19^ab^ | 0.24^a^ | 0.14^b^ | 0.011 | 0.021 |
|  | *[Eubacterium]_hallii_group* | 0.14^ab^ | 0.18^a^ | 0.11^b^ | 0.011 | 0.031 |
|  | *[Eubacterium]_oxidoreducens_group* | 0.11^b^ | 0.19^a^ | 0.11^b^ | 0.011 | 0.044 |
|  | *Family_XIII_UCG-002* | 0.12^ab^ | 0.13^a^ | 0.08^b^ | 0.007 | 0.004 |
|  | *Lachnospiraceae_UCG-006* | 0.08^b^ | 0.15^a^ | 0.08^b^ | 0.011 | 0.015 |
|  | *Tyzzerella_3* | 0.04^b^ | 0.19^a^ | 0.04^b^ | 0.022 | 0.006 |
|  | *Anaerotruncus* | 0.06^b^ | 0.13^a^ | 0.06^b^ | 0.011 | 0.006 |
|  | *[Eubacterium]_ventriosum_group* | 0.06^b^ | 0.13^a^ | 0.05^b^ | 0.013 | 0.043 |
|  | *Lachnospira* | 0.01^b^ | 0.17^a^ | 0.01^b^ | 0.023 | 0.022 |
| Proteobacteria | *Ruminobacter* | 1.90^a^ | 0.16^c^ | 1.14^b^ | 0.206 | <0.001 |
|  | *Succinivibrionaceae_UCG-002* | 1.34^a^ | 0.08^c^ | 0.54^b^ | 0.151 | 0.004 |
| Saccharibacteria | *Candidatus_Saccharimonas* | 0.75^b^ | 1.16^a^ | 1.01^b^ | 0.048 | 0.037 |
| SR1_Absconditabacteria | *norank_p_SR1_Absconditabacteria* | 0.13^b^ | 0.28^a^ | 0.22^a^ | 0.027 | 0.006 |
| Only bacterial genera (accounting for ≥ 0.1% in at least one of the samples) affected by treatments are listed. Diets: C, corn and corn silage diet; L, sugar beet pulp and alfalfa silage diet; S, steam-flaked corn and corn silage diet. SEM, standard error of the mean. ^a, b, c^ means within a row with different superscripts differ significantly (*P* ≤ 0.05). | | | | | | |

| **Table S3. Effect of two glucogenic (C, S) and a lipogenic (L) diet on the relative abundance (%) of ruminal archaea after 48 h *in vitro* fermentation with rumen fluid of dairy cows** | | | | | | |
| --- | --- | --- | --- | --- | --- | --- |
| Phylum | Genus/others | Experimental diet | | | SEM | *P*-value |
|  |  | C | L | S |  |  |
| Euryarchaeota | *Methanobrevibacter* | 78.3^a^ | 57.6^b^ | 74.0^a^ | 3.382 | 0.014 |
|  | *norank_f__Thermoplasmatales_Incertae_Sedis* | 17.7 | 30.8 | 21.4 | 2.348 | 0.125 |
|  | *Candidatus_Methanomethylophilus* | 3.68^b^ | 11.4^a^ | 4.40^b^ | 1.067 | 0.001 |
|  | *Methanosphaera* | 0.45 | 0.39 | 0.38 | 0.032 | 0.798 |
|  | *Halostagnicola* | 0.04 | 0.02 | 0.04 | 0.003 | 0.076 |
| Diets: C, corn and corn silage diet; L, sugar beet pulp and alfalfa silage diet; S, steam-flaked corn and corn silage diet. SEM, standard error of the mean.  ^a, b^ means within a row with different superscripts differ significantly (*P* ≤ 0.05). | | | | | | |

| **Table S4. Effects of two glucogenic (C, S) and a lipogenic (L) diet on the relative abundance (%) of the KEGG pathways of ruminal bacteria predicted by PICRUSt after 48 h *in vitro* fermentation with rumen fluid of dairy cows** | | | | | |
| --- | --- | --- | --- | --- | --- |
| Category/pathway | Experimental diet | | | SEM | *P*-value |
|  | C | L | S |  |  |
| Metabolism |  |  |  |  |  |
| Amino Acid Metabolism | 10.67 | 10.39 | 10.67 | 0.053 | 0.053 |
| Carbohydrate Metabolism | 9.90 | 9.97 | 9.93 | 0.766 | 0.766 |
| Energy Metabolism | 6.18 | 6.15 | 6.08 | 0.104 | 0.104 |
| Metabolism of Cofactors and Vitamins | 4.66 | 4.54 | 4.63 | 0.055 | 0.055 |
| Nucleotide Metabolism | 4.38 | 4.29 | 4.37 | 0.080 | 0.080 |
| Glycan Biosynthesis and Metabolism | 3.10 | 2.82 | 3 | 0.054 | 0.054 |
| Lipid Metabolism | 2.79 | 2.80 | 2.77 | 0.750 | 0.750 |
| Enzyme Families | 2.25 | 2.21 | 2.25 | 0.165 | 0.165 |
| Metabolism of Terpenoids and Polyketides | 1.80 | 1.76 | 1.79 | 0.250 | 0.250 |
| Metabolism of Other Amino Acids | 1.63 | 1.57 | 1.63 | 0.063 | 0.063 |
| Xenobiotics Biodegradation and Metabolism | 1.49 | 1.49 | 1.51 | 0.222 | 0.222 |
| Biosynthesis of Other Secondary Metabolites | 1.07 | 1.03 | 1.07 | 0.170 | 0.170 |
| Environmental Information Processing |  |  |  |  |  |
| Membrane Transport | 9.26 | 10.12 | 9.45 | 0.092 | 0.092 |
| Signal Transduction | 1.42^b^ | 1.50^a^ | 1.42^b^ | 0.027 | 0.027 |
| Signalling Molecules and Interaction | 0.16 | 0.16 | 0.16 | 0.168 | 0.168 |
| Genetic Information Processing |  |  |  |  |  |
| Replication and Repair | 9.72^a^ | 9.51^b^ | 9.71^a^ | 0.020 | 0.020 |
| Translation | 6.42^a^ | 6.30^b^ | 6.41^a^ | 0.031 | 0.031 |
| Folding, Sorting and Degradation | 2.68 | 2.63 | 2.63 | 0.329 | 0.329 |
| Transcription | 2.28 | 2.40 | 2.32 | 0.075 | 0.075 |
| Unclassified |  |  |  |  |  |
| Cellular Processes and Signaling | 3.90^a^ | 3.81^b^ | 3.91^a^ | 0.004 | 0.004 |
| Genetic Information Processing | 2.68 | 2.68 | 2.69 | 0.843 | 0.843 |
| Metabolism | 2.44 | 2.41 | 2.45 | 0.107 | 0.107 |
| Cellular Processes |  |  |  |  |  |
| Cell Motility | 1.71^b^ | 2.21^a^ | 1.74^b^ | 0.030 | 0.030 |
| Cell Growth and Death | 0.59 | 0.58 | 0.59 | 0.628 | 0.628 |
| Transport and Catabolism | 0.41 | 0.38 | 0.38 | 0.195 | 0.195 |
| Organismal Systems |  |  |  |  |  |
| Endocrine System | 0.31 | 0.30 | 0.31 | 0.739 | 0.739 |
|  |  |  |  |  |  |
| **Table S4 (continued).** | | | | | |
| Category/pathway | Experimental diet | | | SEM | *P*-value |
|  | C | L | S |  |  |
| Environmental Adaptation | 0.14^b^ | 0.15^a^ | 0.14^b^ | 0.026 | 0.026 |
| Nervous System | 0.10 | 0.10 | 0.1 | 0.587 | 0.587 |
| Immune System | 0.089 | 0.092 | 0.087 | 0.399 | 0.399 |
| Digestive System | 0.051^a^ | 0.044^b^ | 0.055^a^ | 0.045 | 0.045 |
| Excretory System | 0.039 | 0.033 | 0.037 | 0.094 | 0.094 |
| Circulatory System | 0.004 | 0.007 | 0.001 | 0.154 | 0.154 |
| Diets: C, corn and corn silage diet; L, sugar beet pulp and alfalfa silage diet; S, steam-flaked corn and corn silage diet. KEGG = Kyoto Encyclopedia of Genes and Genomes. PICRUSt, Phylogenetic Investigation of Communities by Reconstruction of Unobserved States. SEM, standard error of the mean.  ^a, b^ means within a row with different superscripts differ significantly (*P* ≤ 0.05). | | | | | |

| **Table S5 Significantly differential metabolites between the lipogenic diet L and glucogenic diet C after 48 h *in vitro* fermentation with rumen fluid of dairy cows** | | | | | | | | |
| --- | --- | --- | --- | --- | --- | --- | --- | --- |
| **HMDB Superclass** | **HMDB Subclass** | **Metabolite** | **M/Z** | **Retention time** | **VIP** | **FC(L/C)** | ***P*-value** | **Mode** |
| Lipids and lipid-like molecules | Fatty acids and conjugates | Myristoleic acid | 191.18 | 9.14 | 2.77 | 1.65 | < 0.001 | pos |
|  |  | Goshuyic acid | 247.17 | 9.13 | 2.16 | 1.57 | < 0.001 | pos |
|  |  | 2-Octenoic acid | 302.23 | 6.36 | 1.71 | 1.33 | < 0.001 | pos |
|  |  | Stearic acid | 307.26 | 7.14 | 1.46 | 1.14 | < 0.001 | pos |
|  |  | 5-Hexyl-2-furanhexanoic acid | 289.18 | 5.85 | 1.19 | 1.09 | < 0.001 | pos |
|  |  | 13-hydroxyoctadecanoic acid | 283.26 | 9.43 | 1.63 | 0.69 | 0.005 | pos |
|  |  | 10-Hydroxy-2,8-decadiene-4,6-diynoic acid | 194.08 | 3.22 | 1.31 | 0.85 | < 0.001 | pos |
|  |  | Pentadecanoic acid | 275.26 | 8.76 | 1.18 | 0.89 | < 0.001 | pos |
|  |  | Petroselinic acid | 283.26 | 10.95 | 1.18 | 0.93 | < 0.001 | pos |
|  |  | Dioscoretine | 242.17 | 4.93 | 1.17 | 1.12 | 0.003 | pos |
|  |  | Floionolic acid | 315.25 | 9.73 | 1.10 | 0.94 | < 0.001 | pos |
|  |  | 2-Octenedioic acid | 217.07 | 2.24 | 2.12 | 2.78 | < 0.001 | neg |
|  |  | 2-hydroxyhexadecanoic acid | 271.23 | 9.10 | 1.34 | 1.10 | < 0.001 | neg |
|  |  | 5-Tetradecenoic acid | 271.19 | 7.16 | 1.27 | 1.33 | < 0.001 | neg |
|  |  | 2-Hydroxymyristic Acid | 243.20 | 8.45 | 1.14 | 1.10 | < 0.001 | neg |
|  |  | (S)-10,16-Dihydroxyhexadecanoic acid | 287.22 | 7.97 | 1.07 | 1.13 | 0.001 | neg |
|  |  | 3,3-Dimethylglutaric acid | 159.07 | 3.81 | 1.38 | 0.81 | < 0.001 | neg |
|  |  | (9S,10S)-9,10-dihydroxyoctadecanoate | 315.25 | 8.59 | 1.24 | 1.27 | 0.019 | neg |
|  |  | 3b,15b,17a-Trihydroxy-pregnenone | 329.21 | 8.09 | 1.23 | 0.54 | 0.039 | neg |
|  |  | (R)-3-Hydroxy-Octadecanoic acid | 345.26 | 9.04 | 1.09 | 0.61 | 0.034 | neg |
|  | Fatty acid esters | Butyl 2-decenoate | 191.18 | 9.22 | 2.85 | 1.85 | < 0.001 | pos |
|  |  | 4,8 dimethylnonanoyl carnitine | 330.26 | 8.46 | 1.05 | 0.92 | < 0.001 | pos |
|  |  | Dipropyl hexanedioate | 213.15 | 4.52 | 1.03 | 0.95 | < 0.001 | pos |
|  |  | Stearoyllactic acid | 355.28 | 9.88 | 2.31 | 2.57 | < 0.001 | neg |
|  |  | Ethyl (Z, Z)-5,8-tetradecadienoate | 297.21 | 6.53 | 1.22 | 1.17 | < 0.001 | neg |
|  |  | Oleoylcarnitine | 462.30 | 8.73 | 1.06 | 0.86 | < 0.001 | neg |
|  | Fatty acyl glycosides | 1-Octen-3-yl primeveroside | 445.20 | 7.93 | 2.23 | 0.71 | < 0.001 | pos |
|  |  | 4-Hydroxyproline galactoside | 258.10 | 3.23 | 1.86 | 0.79 | < 0.001 | pos |
|  |  | 6S,9R-Dihydroxy-4,7E-megastigmadien-3-one 9-[apiosyl-(1->6)-glucoside] | 563.23 | 8.41 | 1.26 | 1.14 | < 0.001 | neg |
|  | Fatty alcohols | 1-Undecanol | 367.36 | 10.50 | 1.64 | 1.90 | < 0.001 | pos |
|  |  | Avocadene | 304.29 | 6.62 | 1.42 | 1.12 | < 0.001 | pos |
|  |  | Avocadyne | 329.23 | 8.91 | 1.24 | 1.32 | < 0.001 | neg |
|  | Triterpenoids | Tomentosolic acid | 437.34 | 9.48 | 3.02 | 1.55 | < 0.001 | pos |
|  |  |  |  |  |  |  |  |  |
| **Table S5** **(continued)** | | | | | | | | |
| **HMDB Superclass** | **HMDB Subclass** | **Metabolite** | **M/Z** | **Retention time** | **VIP** | **FC(L/C)** | ***P*-value** | **Mode** |
|  |  | Ursolic acid | 457.37 | 8.90 | 3.00 | 1.56 | < 0.001 | pos |
|  |  | Camelledionol | 423.33 | 8.71 | 2.84 | 1.52 | < 0.001 | pos |
|  |  | Oleanolic acid | 457.37 | 9.05 | 2.59 | 1.61 | < 0.001 | pos |
|  |  | Sandosapogenol | 439.36 | 8.90 | 2.55 | 1.31 | < 0.001 | pos |
|  |  | (3beta,17alpha,23S,24S)-17,23-Epoxy-3,24,29-trihydroxy-27-norlanost-8-en-15-one | 457.33 | 7.07 | 2.37 | 1.48 | < 0.001 | pos |
|  |  | Asperagenin | 449.33 | 7.19 | 1.79 | 1.18 | < 0.001 | pos |
|  |  | Gamma-Taraxastane-3,20-diol | 427.39 | 10.37 | 1.34 | 1.10 | 0.001 | pos |
|  |  | Lucidenic acid E2 | 539.26 | 9.24 | 1.33 | 1.07 | < 0.001 | pos |
|  |  | Ganoderenic acid E | 551.27 | 8.85 | 1.01 | 1.04 | < 0.001 | pos |
|  |  | 3beta-Acetoxy-19alpha-hydroxy-12-ursene | 502.42 | 10.48 | 1.71 | 0.57 | 0.019 | pos |
|  |  | Glyuranolide | 530.34 | 10.24 | 1.29 | 1.12 | 0.012 | pos |
|  |  | Melilotoside D | 527.29 | 7.52 | 1.21 | 0.91 | < 0.001 | pos |
|  |  | Medicoside C | 461.25 | 7.59 | 1.04 | 0.94 | < 0.001 | pos |
|  |  | Glabric acid | 531.33 | 6.84 | 2.31 | 1.65 | < 0.001 | neg |
|  |  | Ganoderic acid A | 497.29 | 8.39 | 2.27 | 1.98 | < 0.001 | neg |
|  |  | Ganoderiol I | 501.36 | 9.14 | 2.22 | 1.34 | < 0.001 | neg |
|  |  | Medicagenic acid | 547.33 | 7.39 | 1.59 | 1.35 | < 0.001 | neg |
|  |  | (3beta,19alpha)-3,19,23,24-Tetrahydroxy-12-oleanen-28-oic acid | 539.33 | 6.96 | 1.54 | 1.19 | < 0.001 | neg |
|  | Sesquiterpenoids | Ipomeatetrahydrofuran | 239.20 | 7.87 | 2.27 | 1.42 | < 0.001 | pos |
|  |  | Auberganol | 241.22 | 8.03 | 1.73 | 1.28 | < 0.001 | pos |
|  |  | 4,7-Megastigmadien-9-ol | 411.33 | 8.63 | 1.22 | 1.08 | < 0.001 | pos |
|  |  | Sterebin A | 343.25 | 5.87 | 1.06 | 1.05 | < 0.001 | pos |
|  |  | 7(14)-Bisabolene-2,3,10,11-tetrol | 255.19 | 6.43 | 2.09 | 0.64 | < 0.001 | pos |
|  |  | Curcumol | 269.21 | 7.04 | 1.26 | 0.85 | < 0.001 | pos |
|  |  | 7-Hydroxytrichodermol | 267.16 | 4.49 | 1.03 | 0.94 | < 0.001 | pos |
|  |  | Deoxynivalenol 3-glucoside | 491.21 | 9.33 | 1.02 | 0.90 | < 0.001 | pos |
|  |  | 7(14)-Farnesene-9,12-diol | 287.22 | 7.80 | 1.26 | 1.35 | < 0.001 | neg |
|  | Terpene lactones | 8-Deoxy-11,13-dihydroxygrosheimin | 298.18 | 4.86 | 1.76 | 0.76 | < 0.001 | pos |
|  |  | Isoalantolactone | 233.15 | 6.28 | 1.43 | 0.90 | < 0.001 | pos |
|  |  | Tatridin B | 229.12 | 6.15 | 1.30 | 0.90 | < 0.001 | pos |
|  |  | Dihydrocumambrin A | 307.16 | 7.83 | 2.29 | 3.19 | < 0.001 | neg |
|  |  | 2alpha-Hydroxyalantolactone | 229.12 | 7.92 | 1.73 | 0.67 | < 0.001 | neg |
|  |  | 4,11,13,15-Tetrahydroridentin B | 249.15 | 7.03 | 1.39 | 0.76 | < 0.001 | neg |
|  |  |  |  |  |  |  |  |  |
| **Table S5** **(continued)** | | | | | | | | |
| **HMDB Superclass** | **HMDB Subclass** | **Metabolite** | **M/Z** | **Retention time** | **VIP** | **FC(L/C)** | ***P*-value** | **Mode** |
|  |  | Tavulin | 245.12 | 6.17 | 1.28 | 0.85 | < 0.001 | neg |
|  |  | Crispolide | 261.11 | 4.48 | 1.18 | 0.83 | < 0.001 | neg |
|  | Monoterpenoids | Withangulatin A | 549.25 | 8.69 | 2.48 | 1.32 | < 0.001 | pos |
|  |  | Linalyl propionate | 243.20 | 5.46 | 1.13 | 1.17 | < 0.001 | pos |
|  |  | Piperitone | 170.15 | 5.71 | 1.44 | 1.34 | < 0.001 | pos |
|  |  | Alpha-Terpineol propanoate | 211.17 | 7.24 | 1.01 | 0.86 | < 0.001 | pos |
|  |  | 3-(5,6,6-Trimethylbicyclo [2.2.1] hept-1-yl) cyclohexanol | 281.21 | 7.58 | 1.64 | 1.46 | < 0.001 | neg |
|  |  | Soyasapogenol B 3-O-b-D-glucuronide | 671.35 | 7.95 | 1.28 | 1.24 | < 0.001 | neg |
|  |  | Valechlorin | 457.17 | 8.02 | 1.38 | 1.13 | < 0.001 | neg |
|  | Steroid lactones | Withaperuvin H | 611.29 | 8.17 | 2.73 | 1.55 | < 0.001 | pos |
|  |  | Physagulin F | 567.26 | 8.21 | 1.80 | 1.19 | < 0.001 | pos |
|  |  | Physagulin C | 565.24 | 8.41 | 1.25 | 1.07 | < 0.001 | pos |
|  |  | Physapubenolide | 565.25 | 8.23 | 1.43 | 1.42 | < 0.001 | neg |
|  |  | (5alpha,6beta,14alpha,20R,22R)-5,6,14,20,27-Pentahydroxy-1-oxowith-24-enolide | 505.28 | 5.66 | 1.35 | 1.20 | < 0.001 | neg |
|  | Diterpenoids | Phytocassane B | 317.21 | 6.59 | 1.29 | 1.14 | < 0.001 | pos |
|  |  | Austroinulin | 355.28 | 6.68 | 1.13 | 1.06 | < 0.001 | pos |
|  |  | Beta-Tocopheryl quinone | 433.37 | 10.23 | 1.96 | 1.90 | < 0.001 | pos |
|  |  | (13R,14R)-7-Labdene-13,14,15-triol | 357.30 | 8.36 | 1.01 | 0.95 | < 0.001 | pos |
|  | Lineolic acids and derivatives | Punicic acid | 243.21 | 7.35 | 1.51 | 0.85 | < 0.001 | pos |
|  |  | 13S-hydroxyoctadecadienoic acid | 329.27 | 9.87 | 1.31 | 0.87 | < 0.001 | pos |
|  |  | 2-Hydroxylinolenic acid | 317.21 | 7.35 | 1.22 | 0.88 | < 0.001 | pos |
|  |  | Cibaric acid | 369.19 | 6.18 | 1.27 | 1.22 | < 0.001 | neg |
|  | Diradylglycerols | DG(16:0/16:0/0:0) | 591.50 | 10.92 | 1.34 | 1.23 | < 0.001 | pos |
|  |  | DG(15:0/18:0/0:0) | 627.52 | 11.53 | 1.31 | 0.77 | < 0.001 | neg |
|  |  | DG(20:1(11Z)/22:5(4Z,7Z,10Z,13Z,16Z)/0:0) | 677.55 | 9.98 | 1.17 | 0.82 | < 0.001 | neg |
|  | Terpene glycosides | Melilotin | 549.30 | 7.50 | 1.33 | 0.87 | < 0.001 | pos |
|  |  | Calenduloside E | 677.39 | 7.77 | 2.64 | 2.72 | < 0.001 | neg |
|  |  | L-Citronellol glucoside | 317.20 | 5.63 | 1.00 | 1.15 | < 0.001 | neg |
|  | Glycerophosphocholines | PC(16:0/18:2(9Z,12Z)) | 802.56 | 11.16 | 1.24 | 0.84 | < 0.001 | neg |
|  |  | LysoPC(16:0) | 540.33 | 8.36 | 1.16 | 0.86 | < 0.001 | neg |
|  | Glycerophosphoethanolamines | PE(14:1(9Z)/16:1(9Z)) | 660.46 | 10.33 | 2.29 | 0.64 | < 0.001 | pos |
|  |  | LysoPE(0:0/16:0) | 436.28 | 8.40 | 1.54 | 0.80 | < 0.001 | pos |
|  | Hydroxysteroids | Tetrahydrodeoxycorticosterone | 379.22 | 8.98 | 1.58 | 1.19 | < 0.001 | pos |
|  |  |  |  |  |  |  |  |  |
| **Table S5** **(continued)** | | | | | | | | |
| **HMDB Superclass** | **HMDB Subclass** | **Metabolite** | **M/Z** | **Retention time** | **VIP** | **FC(L/C)** | ***P*-value** | **Mode** |
|  |  | Prednisone | 379.15 | 8.92 | 1.13 | 0.85 | < 0.001 | neg |
|  | Pregnane steroids | 17a-Hydroxypregnenolone | 333.24 | 5.75 | 2.83 | 1.77 | < 0.001 | pos |
|  |  | Halobetasol Propionate | 521.13 | 7.58 | 1.97 | 1.86 | < 0.001 | neg |
|  | Steroidal glycosides | Corchoroside B | 541.28 | 9.01 | 1.48 | 1.12 | < 0.001 | pos |
|  |  | Torvoside D | 747.39 | 7.73 | 2.25 | 1.86 | < 0.001 | neg |
|  | Bile acids, alcohols and derivatives | 5beta-Cholestane-3alpha,7alpha,24,26-tetrol | 401.34 | 7.36 | 1.16 | 0.92 | < 0.001 | pos |
|  | Glycerophosphates | 1-(9Z-tetradecenoyl)-glycero-3-phosphate | 379.19 | 8.11 | 1.31 | 0.66 | < 0.001 | neg |
|  | Cholestane steroids | 5a-Cholest-8-en-3b-ol | 409.35 | 10.61 | 1.01 | 0.89 | < 0.001 | pos |
|  | Fatty amides | Palmitoyl Serinol | 330.30 | 8.94 | 1.89 | 1.36 | < 0.001 | pos |
|  | Monoradylglycerols | MG(i-12:0/0:0/0:0) | 255.20 | 8.07 | 1.40 | 1.18 | < 0.001 | neg |
|  | Quinone and hydroquinone lipids | 13'-Carboxy-alpha-tocopherol | 493.39 | 11.52 | 1.74 | 0.69 | < 0.001 | pos |
|  | Steroid esters | Physapubescin | 553.28 | 7.95 | 1.76 | 1.17 | < 0.001 | pos |
|  | Stigmastanes and derivatives | 6-Deoxohomodolichosterone | 463.38 | 10.59 | 1.11 | 1.12 | < 0.001 | pos |
|  | Eicosanoids | Carboprost Tromethamine | 367.25 | 7.62 | 1.27 | 0.81 | < 0.001 | neg |
|  | Ergostane steroids | Delta 8,14 -Sterol | 393.35 | 12.76 | 1.08 | 0.91 | < 0.001 | pos |
| Organic acids and derivatives | Amino acids, peptides, and analogues | N6-Acetyl-5S-hydroxy-L-lysine | 243.08 | 4.68 | 2.31 | 0.42 | < 0.001 | pos |
|  |  | N-[[3-Hydroxy-2-(2-pentenyl) cyclopentyl]acetyl]isoleucine | 308.22 | 7.67 | 1.93 | 1.27 | < 0.001 | pos |
|  |  | Prolyl-Tyrosine | 323.10 | 5.66 | 1.48 | 1.27 | 0.007 | pos |
|  |  | 5-Hydroxyindoleacetylglycine | 213.07 | 3.25 | 1.04 | 1.13 | 0.004 | pos |
|  |  | Cytidine 2',3'-cyclic phosphate | 350.01 | 5.50 | 2.13 | 0.68 | 0.001 | pos |
|  |  | Valyl-Glutamate | 229.12 | 1.00 | 1.75 | 0.83 | < 0.001 | pos |
|  |  | Isoleucyl-Glutamate | 243.13 | 1.41 | 1.72 | 0.85 | < 0.001 | pos |
|  |  | Tetrahydrodipicolinate | 204.09 | 1.57 | 1.66 | 0.80 | < 0.001 | pos |
|  |  | Phenylalanylproline | 245.13 | 3.54 | 1.29 | 0.91 | < 0.001 | pos |
|  |  | Glutamylleucine | 243.13 | 0.76 | 1.02 | 0.95 | < 0.001 | pos |
|  |  | 4-Hydroxystachydrine | 158.08 | 3.22 | 1.60 | 1.61 | 0.001 | neg |
|  |  | N-Acetylproline | 138.06 | 2.85 | 1.34 | 1.46 | 0.001 | neg |
|  |  | N-Arachidonoyl glycine | 406.26 | 8.05 | 1.24 | 0.71 | 0.002 | neg |
|  |  | 2-Hepteneoylglycine | 230.10 | 3.07 | 2.25 | 0.35 | < 0.001 | neg |
|  |  | Captopril-cysteine disulfide | 371.05 | 8.00 | 1.96 | 0.37 | < 0.001 | neg |
|  |  |  |  |  |  |  |  |  |
|  |  |  |  |  |  |  |  |  |
| **Table S5** **(continued)** | | | | | | | | |
| **HMDB Superclass** | **HMDB Subclass** | **Metabolite** | **M/Z** | **Retention time** | **VIP** | **FC(L/C)** | ***P*-value** | **Mode** |
|  |  | (1R)-Glutathionyl-(2R)-hydroxy-1,2-dihydronaphthalene | 472.12 | 5.47 | 1.78 | 0.52 | < 0.001 | neg |
|  |  | N-(4-Hydroxycinnamoyl) tyrosine | 362.08 | 5.41 | 1.54 | 0.75 | < 0.001 | neg |
|  |  | Indolylacryloylglycine | 225.07 | 3.35 | 1.41 | 0.89 | < 0.001 | neg |
|  |  | Gamma-Glutamyl-S-methylcysteine sulfoxide | 279.07 | 4.76 | 1.20 | 0.82 | 0.043 | neg |
|  |  | 3-Hydroxymugineic acid | 357.09 | 5.55 | 1.16 | 0.81 | 0.027 | neg |
|  | Hybrid peptides | Beta-Alanyl-L-lysine | 476.32 | 8.31 | 1.77 | 0.80 | < 0.001 | pos |
|  | Carboxylic acid derivatives | Dihydroceramide | 374.29 | 8.95 | 1.53 | 1.40 | < 0.001 | neg |
|  | Medium-chain hydroxy acids and derivatives | 12-Hydroxydodecanoic acid | 258.21 | 5.72 | 1.47 | 0.85 | < 0.001 | pos |
|  | Organic carbonic acids | Moracin L | 342.13 | 5.06 | 1.39 | 0.78 | < 0.001 | pos |
| Organic oxygen compounds | Carbohydrates and carbohydrate conjugates | 3,4,5-trihydroxy-6-[(4-methoxy-1-benzofuran-6-yl) oxy]oxane-2-carboxylic acid | 363.07 | 6.81 | 1.89 | 1.35 | < 0.001 | pos |
|  |  | 4-Methoxybenzyl O-(2-sulfoglucoside) | 345.06 | 6.38 | 1.87 | 1.71 | 0.002 | pos |
|  |  | Arbutin | 317.06 | 9.96 | 1.48 | 1.19 | 0.001 | pos |
|  |  | Indican | 296.11 | 2.97 | 2.84 | 0.66 | < 0.001 | pos |
|  |  | N-Acetylneuraminic acid | 274.09 | 2.80 | 2.10 | 0.63 | < 0.001 | pos |
|  |  | 6-(5-ethyl-2,3-dihydroxyphenoxy)-3,4,5-trihydroxyoxane-2-carboxylic acid | 295.08 | 3.42 | 1.85 | 1.47 | 0.001 | pos |
|  |  | (2S)-2-Butanol O-[b-D-Apiofuranosyl-(1->6)-b-D-glucopyranoside] | 410.20 | 8.36 | 1.22 | 0.90 | < 0.001 | pos |
|  |  | Lotaustralin | 226.11 | 2.79 | 1.17 | 0.94 | < 0.001 | pos |
|  |  | Dihydromaleimide beta-D-glucoside | 260.08 | 1.40 | 2.79 | 0.24 | < 0.001 | neg |
|  |  | Tyramine glucuronide | 294.10 | 2.97 | 2.35 | 0.68 | < 0.001 | neg |
|  |  | Pisatoside | 260.08 | 1.63 | 2.26 | 0.57 | < 0.001 | neg |
|  |  | 3-oxo-3-[(3,4,5,6-tetrahydroxyoxan-2-yl) methoxy] propanoic acid | 247.05 | 2.90 | 1.57 | 0.74 | < 0.001 | neg |
|  |  | Fluoxetine glucuronide | 520.14 | 7.36 | 1.55 | 0.52 | 0.003 | neg |
|  |  | 6-Sinapoylglucoraphenin | 640.08 | 0.83 | 1.08 | 0.91 | < 0.001 | neg |
|  | Carbonyl compounds | 3-Propyl-1,2-cyclopentanedione | 182.12 | 4.51 | 1.99 | 0.48 | 0.006 | pos |
|  |  | 4-Heptanone | 115.11 | 3.45 | 1.01 | 1.11 | 0.003 | pos |
|  |  | 3-Methylcyclopentadecanone | 283.23 | 8.91 | 1.14 | 1.20 | 0.001 | neg |
|  |  | 2-Nonanone | 187.13 | 4.81 | 1.07 | 1.17 | < 0.001 | neg |
|  |  | 1-Hydroxyepiacorone | 289.12 | 3.60 | 1.33 | 0.77 | < 0.001 | neg |
|  |  |  |  |  |  |  |  |  |
|  |  |  |  |  |  |  |  |  |
| **Table S5** **(continued)** | | | | | | | | |
| **HMDB Superclass** | **HMDB Subclass** | **Metabolite** | **M/Z** | **Retention time** | **VIP** | **FC(L/C)** | ***P*-value** | **Mode** |
| Organoheterocyclic compounds | 1-benzopyrans | Theaflagallin | 365.06 | 7.11 | 2.15 | 1.45 | 0.010 | pos |
|  |  |  |  |  |  |  |  |  |
|  |  | 9'-Carboxy-gamma-chromanol | 421.26 | 7.53 | 1.19 | 1.20 | < 0.001 | neg |
|  |  | Gamma-CEHC | 263.13 | 5.26 | 1.30 | 0.75 | 0.001 | neg |
|  | Hydropyridines | (E)-5-(3,4,5,6-Tetrahydro-3-pyridylidenemethyl)-2-furanmethanol | 209.13 | 2.85 | 1.09 | 0.88 | 0.015 | pos |
|  |  | 2-Hydroxypyridine | 140.04 | 2.51 | 1.46 | 1.58 | < 0.001 | neg |
|  |  | 5-(2-Furanyl)-1,2,3,4,5,6-hexahydro-7H-cyclopenta[b]pyridin-7-one | 248.09 | 3.96 | 1.24 | 0.72 | 0.001 | neg |
|  | Gamma butyrolactones | 5-Nonyltetrahydro-2-oxo-3-furancarboxylic acid | 237.15 | 6.26 | 1.07 | 1.16 | 0.002 | neg |
|  |  | Artabsinolide A | 261.11 | 6.13 | 1.16 | 0.73 | 0.012 | neg |
|  |  | 3-Hydroxyadipic acid 3,6-lactone | 143.04 | 1.62 | 1.15 | 0.68 | 0.009 | neg |
|  | Indolyl carboxylic acids and derivatives | L-Tryptophan | 426.22 | 2.88 | 1.43 | 1.19 | 0.017 | pos |
|  |  | Indoleacetic acid | 220.06 | 3.70 | 1.16 | 0.56 | 0.029 | neg |
|  |  | 5-Hydroxyindoleacetic acid | 190.05 | 3.53 | 1.39 | 0.70 | 0.005 | neg |
|  | Dibenzoxepines | Doxepin N-oxide glucuronide | 507.17 | 7.28 | 2.11 | 2.19 | 0.011 | neg |
|  | Benzoxazinones | (R)-2,7-Dihydroxy-2H-1,4-benzoxazin-3(4H)-one | 380.11 | 5.58 | 1.62 | 0.81 | < 0.001 | pos |
|  | Benzylisoquinolines | 8-Propanoylneosolaniol | 473.16 | 7.24 | 1.92 | 0.64 | 0.001 | neg |
|  | Bilirubins | D-Urobilin | 609.27 | 8.19 | 2.16 | 1.79 | < 0.001 | neg |
|  | Hydroxyindoles | 11-beta-Hydroxyandrosterone-3-glucuronide | 463.24 | 8.09 | 1.57 | 2.22 | 0.041 | neg |
|  | Indoles | Indoleacrylic acid | 188.07 | 3.41 | 1.62 | 0.81 | < 0.001 | pos |
|  | Methylpyridines | 2-Ethyl-5-methylpyridine | 139.12 | 1.87 | 1.51 | 1.41 | 0.020 | pos |
|  | Pyranones and derivatives | Erinapyrone A | 187.06 | 2.69 | 1.17 | 1.21 | < 0.001 | neg |
|  | Quinolones and derivatives | Quinoline-4,8-diol | 206.05 | 2.91 | 1.15 | 0.85 | 0.007 | neg |
|  | Unclassified | Canescein | 611.25 | 8.45 | 2.77 | 1.84 | < 0.001 | pos |
|  | Unclassified | (+)-2,3-Dihydro-3-methyl-1H-pyrrole | 125.11 | 1.14 | 1.43 | 1.16 | 0.018 | pos |
|  | Unclassified | (S)-N-Methylsalsolinol | 158.10 | 3.24 | 1.42 | 0.80 | 0.001 | pos |
|  | Unclassified | Isoquinoline | 130.06 | 4.87 | 1.21 | 0.90 | < 0.001 | pos |
|  | Unclassified | 3-Pyridinebutanoic acid | 210.08 | 4.75 | 1.17 | 1.26 | < 0.001 | neg |
|  | Unclassified | N-Carbamoyl glucuronide lorcaserin | 454.17 | 8.36 | 1.42 | 0.74 | < 0.001 | neg |
|  |  |  |  |  |  |  |  |  |
|  |  |  |  |  |  |  |  |  |
| **Table S5** **(continued)** | | | | | | | | |
| **HMDB Superclass** | **HMDB Subclass** | **Metabolite** | **M/Z** | **Retention time** | **VIP** | **FC(L/C)** | ***P*-value** | **Mode** |
| Phenylpropanoids and polyketides | Furanocoumarins | Edulisin I | 525.15 | 8.03 | 1.27 | 1.21 | 0.006 | neg |
|  |  | (R)-Heraclenol 2'-(3-methyl-2-butenoate) | 385.13 | 7.70 | 1.11 | 0.75 | 0.008 | neg |
|  | Hydroxycinnamic acids and derivatives | Sinapinic acid-O-glucuronide isomer | 365.09 | 4.79 | 1.23 | 1.22 | 0.034 | pos |
|  |  | N-(p-Hydroxyphenyl) ethyl p-hydroxycinnamide | 318.09 | 4.63 | 1.45 | 1.91 | 0.025 | neg |
|  | Anthocyanidins | 2-{2-[(6-carboxy-3,4,5-trihydroxyoxan-2-yl) oxy] phenyl}-1lambda⁴-chromen-1-ylium | 434.08 | 5.84 | 1.90 | 1.79 | 0.025 | neg |
|  | Coumarin glycosides | Aesculin | 363.07 | 7.11 | 1.94 | 1.32 | 0.012 | pos |
|  | Cyclic diarylheptanoids | Myricatomentoside II | 571.16 | 8.02 | 1.37 | 1.31 | 0.010 | neg |
|  | Flavans | 2-phenyl-3,4-dihydro-2H-1-benzopyran-3,5,7-triol | 259.10 | 3.88 | 1.03 | 0.83 | 0.044 | pos |
|  | Flavonoid glycosides | Delphinidin 3-rutinoside | 594.16 | 7.01 | 1.06 | 0.85 | 0.008 | pos |
|  | Furanoisoflavonoids | Kanzonol F | 443.18 | 7.09 | 1.52 | 1.43 | 0.040 | pos |
|  | Pyranocoumarins | Trans-O-Methylgrandmarin | 327.09 | 3.20 | 1.06 | 0.91 | 0.001 | neg |
|  | Pyranoflavonoids | Cycloartocarpin | 479.17 | 7.66 | 1.56 | 1.41 | 0.002 | neg |
|  | Stilbene glycosides | 3,4,5-trihydroxy-6-(2-hydroxy-1,2-diphenylethoxy) oxane-2-carboxylic acid | 408.17 | 4.48 | 1.29 | 0.89 | < 0.001 | pos |
|  | Unclassified | 3-(1,2-dihydroxybutyl)-7-hydroxy-1H-isochromen-1-one | 268.12 | 4.98 | 1.84 | 0.58 | 0.001 | pos |
|  |  | Neosaxitoxin | 280.12 | 4.14 | 1.60 | 0.79 | < 0.001 | pos |
|  |  | Yuzu lactone | 197.15 | 6.77 | 1.29 | 0.88 | < 0.001 | pos |
|  |  | Alpha-methylphenylalanine | 180.10 | 3.65 | 1.23 | 0.91 | < 0.001 | pos |
|  |  | Coriandrone C | 264.09 | 3.02 | 1.19 | 0.90 | < 0.001 | pos |
|  |  | Norpropoxyphene | 370.20 | 7.44 | 2.07 | 2.55 | < 0.001 | neg |
|  |  | 6-[(1Z)-2-hydroxy-3-oxobut-1-en-1-yl]-7-methoxy-2H-chromen-2-one | 305.07 | 4.32 | 1.24 | 1.22 | 0.011 | neg |
| Benzenoids | Benzoic acids and derivatives | Antibiotic SB 202742 | 393.24 | 9.47 | 1.97 | 1.62 | < 0.001 | pos |
|  |  | 3,5-Bis(1,1-dimethylethyl)-4-hydroxy-benzoic acid ethyl ester | 573.34 | 8.00 | 1.59 | 1.66 | 0.001 | neg |
|  |  | Salicylic acid | 137.02 | 4.65 | 1.04 | 1.07 | < 0.001 | neg |
|  | Aniline and substituted anilines | OR-1855 | 204.11 | 2.94 | 1.51 | 0.64 | 0.013 | pos |
|  | Anisoles | 1-(4-methoxyphenyl)-4-methylpentan-3-ol | 461.29 | 8.43 | 1.12 | 0.76 | 0.004 | neg |
|  | Benzenediols | (Z, Z)-2-Methyl-5-(8,11,14-pentadecatrienyl)-1,3-benzenediol | 392.25 | 7.10 | 1.09 | 1.14 | 0.023 | pos |
|  |  |  |  |  |  |  |  |  |
| **Table S5** **(continued)** | | | | | | | | |
| **HMDB Superclass** | **HMDB Subclass** | **Metabolite** | **M/Z** | **Retention time** | **VIP** | **FC(L/C)** | ***P*-value** | **Mode** |
|  |  | Pyrocatechol | 109.03 | 3.14 | 2.08 | 0.54 | 0.004 | neg |
|  | Indanones | Pterosin N | 279.12 | 3.41 | 1.09 | 0.81 | 0.004 | neg |
|  | Methoxybenzenes | Homoveratric acid | 195.07 | 3.72 | 1.06 | 1.26 | 0.007 | neg |
|  | Unclassified | 2-Phenoxyethanol | 180.10 | 3.16 | 1.19 | 0.93 | < 0.001 | pos |
|  |  | 1-Phenylethanol | 121.07 | 4.20 | 1.74 | 1.43 | < 0.001 | neg |
|  |  | 5-Phenyl-1,3-oxazinane-2,4-dione | 226.03 | 4.40 | 1.30 | 1.11 | 0.003 | neg |
| Alkaloids and derivatives | Unclassified | Harmalol | 181.08 | 3.35 | 1.24 | 0.89 | < 0.001 | neg |
| Organosulfur compounds | Aryl thioethers | Phenyl vinyl sulfide | 181.03 | 6.43 | 1.91 | 0.46 | 0.001 | neg |
| Hydrocarbon derivatives | Tropolones | Beta-Thujaplicin | 209.08 | 3.69 | 1.04 | 0.77 | 0.043 | neg |
| Others |  | 15(R)-15-methyl Prostaglandin A2 | 349.24 | 4.87 | 3.16 | 2.17 | < 0.001 | pos |
|  |  | (3beta,5xi,9xi,18xi) -Olean-12-en-28-oic acid,3-hydroxy- | 439.36 | 9.22 | 2.90 | 1.43 | < 0.001 | pos |
|  |  | 2-aminohexadecanoic acid | 272.26 | 7.49 | 1.92 | 1.42 | < 0.001 | pos |
|  |  | 2-oxo-pentadecanoic acid | 239.20 | 8.05 | 1.78 | 1.21 | < 0.001 | pos |
|  |  | Oleoyl dopamine | 418.33 | 8.58 | 1.71 | 1.19 | < 0.001 | pos |
|  |  | 4-formyl Indole | 146.06 | 2.98 | 1.68 | 1.32 | 0.001 | pos |
|  |  | 7alpha,12alpha,24-trihydroxycholest-4-en-3-one | 433.33 | 7.78 | 1.60 | 1.21 | < 0.001 | pos |
|  |  | 16,16-dimethyl-PGE1 | 383.28 | 7.32 | 1.59 | 1.19 | < 0.001 | pos |
|  |  | 3,5,7-Trimethyl-2E,4E,6E,8E-decatetraene | 177.16 | 8.85 | 1.58 | 1.17 | < 0.001 | pos |
|  |  | Hydroxy-gamma-sanshool | 290.21 | 4.42 | 1.55 | 0.78 | 0.034 | pos |
|  |  | 1alpha,25-dihydroxy-26,27-ethanovitamin D3 | 443.35 | 8.68 | 1.49 | 1.11 | < 0.001 | pos |
|  |  | PG (16:0/0:0) [U] | 467.28 | 10.18 | 1.46 | 1.09 | 0.001 | pos |
|  |  | 1,8-Diazacyclotetradecane-2,9-dione | 227.17 | 2.88 | 1.43 | 1.18 | 0.022 | pos |
|  |  | Nuatigenin | 431.32 | 8.03 | 1.35 | 1.12 | < 0.001 | pos |
|  |  | Pfaffic acid | 441.34 | 9.13 | 1.33 | 1.08 | 0.001 | pos |
|  |  | Vitamin D2 (Ergocalciferol) | 397.35 | 9.13 | 1.32 | 1.10 | 0.001 | pos |
|  |  | 9-hydroxy-10-oxo-12(Z)-octadecenoic acid | 335.22 | 6.68 | 1.26 | 1.05 | < 0.001 | pos |
|  |  | Coumestrol | 269.04 | 5.34 | 1.25 | 1.18 | 0.002 | pos |
|  |  | Orcinol | 125.06 | 2.67 | 1.25 | 1.16 | 0.005 | pos |
|  |  | Alpha-9(10)-EpODE | 295.23 | 6.49 | 1.22 | 1.06 | < 0.001 | pos |
|  |  | (±)12,13-DiHOME | 337.23 | 6.76 | 1.20 | 1.05 | < 0.001 | pos |
|  |  |  |  |  |  |  |  |  |
| **Table S5** **(continued)** | | | | | | | | |
| **HMDB Superclass** | **HMDB Subclass** | **Metabolite** | **M/Z** | **Retention time** | **VIP** | **FC(L/C)** | ***P*-value** | **Mode** |
|  |  | Amprotropine | 308.22 | 4.74 | 1.20 | 1.08 | 0.001 | pos |
|  |  | Tetranor-12(R)-HETE | 267.19 | 5.89 | 1.11 | 1.07 | < 0.001 | pos |
|  |  | 9,10-epoxy-13-hydroxy-11-octadecenoic acid | 313.24 | 6.49 | 1.04 | 1.04 | < 0.001 | pos |
|  |  | TG (12:0/13:0/20:5(5Z,8Z,11Z,14Z,17Z))[iso6] | 755.61 | 11.02 | 1.03 | 1.15 | 0.009 | pos |
|  |  | 13Z-Docosenamide | 338.34 | 10.15 | 1.00 | 1.04 | 0.035 | pos |
|  |  | 6-hydroxysphingosine | 316.28 | 5.81 | 2.52 | 0.68 | < 0.001 | pos |
|  |  | NORELEAGNINE | 173.11 | 3.22 | 2.50 | 0.50 | < 0.001 | pos |
|  |  | 8-Isoquinoline methanamine (hydrochloride) | 158.10 | 3.33 | 2.42 | 0.71 | < 0.001 | pos |
|  |  | Docosahexaenoyl Glycine | 386.27 | 9.00 | 2.06 | 0.53 | < 0.001 | pos |
|  |  | 3-Methyloxyindole | 148.08 | 4.43 | 1.62 | 0.85 | 0.009 | pos |
|  |  | 1-ACETYLPIPERIDINE | 128.11 | 3.56 | 1.60 | 1.28 | 0.027 | pos |
|  |  | Quadrone | 249.15 | 6.22 | 1.58 | 0.81 | < 0.001 | pos |
|  |  | Cis-5-dodecenoic acid | 199.17 | 6.74 | 1.48 | 0.84 | < 0.001 | pos |
|  |  | (±)-Equol | 243.10 | 5.11 | 1.44 | 0.88 | < 0.001 | pos |
|  |  | Malvidin | 331.08 | 5.08 | 1.42 | 1.18 | 0.008 | pos |
|  |  | 2,3,5-Trimethacarb | 194.12 | 3.51 | 1.41 | 0.89 | < 0.001 | pos |
|  |  | R-Palmitoyl-(2-methyl) Ethanolamide | 314.30 | 7.48 | 1.37 | 1.13 | 0.001 | pos |
|  |  | Lumichrome | 526.18 | 4.17 | 1.34 | 1.15 | 0.001 | pos |
|  |  | PE (16:1(5Z)/16:1(5Z)) | 688.49 | 10.81 | 1.27 | 0.91 | 0.001 | pos |
|  |  | Confertifoline | 235.17 | 6.64 | 1.23 | 0.91 | < 0.001 | pos |
|  |  | Farnesyl acetone | 263.24 | 11.53 | 1.16 | 0.91 | 0.002 | pos |
|  |  | 9S,10R-Epoxy-6Z-octadecene | 570.54 | 12.18 | 1.14 | 0.92 | 0.001 | pos |
|  |  | PG (17:1(9Z)/0:0) | 497.29 | 10.39 | 1.10 | 1.10 | 0.018 | pos |
|  |  | 9,10-Epoxy-18-hydroxystearate | 315.25 | 8.36 | 1.10 | 0.93 | < 0.001 | pos |
|  |  | PE (16:0/0:0) | 454.29 | 8.59 | 1.07 | 1.06 | 0.007 | pos |
|  |  | (+)-Prosopinine | 314.27 | 4.51 | 1.06 | 0.93 | < 0.001 | pos |
|  |  | C-2 Ceramide | 342.30 | 9.05 | 1.03 | 0.93 | < 0.001 | pos |
|  |  | Bifemelane (M4) | 269.12 | 5.13 | 1.00 | 0.96 | < 0.001 | pos |
|  |  | P-Salicylic acid | 137.02 | 4.20 | 1.14 | 1.09 | < 0.001 | neg |
|  |  | MEDICA 16 | 341.27 | 8.94 | 1.02 | 1.08 | < 0.001 | neg |
|  |  | Leu-Trp-OH | 424.15 | 6.84 | 1.43 | 1.82 | 0.010 | neg |
|  |  | SIMMONDSIN-2'-FERULATE | 550.19 | 8.11 | 1.18 | 0.86 | < 0.001 | neg |
| Diets: C, corn and corn silage diet; L, sugar beet pulp and alfalfa silage diet; HMDB, human metabolome database; M/Z, mass-to-charge ratio; FC, fold change; VIP, variable importance in the projection; pos, in positive ion mode; neg, in negative ion mode. | | | | | | | | |

| **Table S6 Significantly differential metabolites between the lipogenic diet L and glucogenic diet S after 48 h *in vitro* fermentation with rumen fluid of dairy cows** | | | | | | | | | | | | | | | | |
| --- | --- | --- | --- | --- | --- | --- | --- | --- | --- | --- | --- | --- | --- | --- | --- | --- |
| **HMDB Superclass** | **HMDB Subclass** | | **Metabolite** | | **M/Z** | | **Retention time** | | **VIP** | | **FC(L/S)** | | ***P*-value** | | **Mode** | |
| Lipids and lipid-like molecules | | Fatty acids and conjugates | | 13-hydroxyoctadecanoic acid | | 283.26 | | 9.43 | | 1.62 | | 0.71 | | 0.005 | | pos |
|  |  |  | | Petroselinic acid | | 283.26 | | 10.95 | | 1.13 | | 0.94 | | < 0.001 | | pos |
|  |  |  | | Myristoleic acid | | 191.18 | | 9.14 | | 2.89 | | 1.69 | | < 0.001 | | pos |
|  | |  | | Goshuyic acid | | 247.17 | | 9.13 | | 2.36 | | 1.68 | | < 0.001 | | pos |
|  | |  | | 12,15-Epoxy-13,14-dimethyleicosa-12,14,16-trienoic acid | | 349.27 | | 8.08 | | 1.79 | | 1.58 | | 0.003 | | pos |
|  | |  | | Stearic acid | | 307.26 | | 7.14 | | 1.50 | | 1.13 | | < 0.001 | | pos |
|  | |  | | 2-Octenoic acid | | 302.23 | | 6.36 | | 1.47 | | 1.26 | | 0.002 | | pos |
|  | |  | | 5-Hexyl-2-furanhexanoic acid | | 289.18 | | 5.85 | | 1.41 | | 1.12 | | 0.000 | | pos |
|  | |  | | Dioscoretine | | 242.17 | | 4.93 | | 1.35 | | 1.14 | | 0.002 | | pos |
|  | |  | | Docosatrienoic acid | | 357.28 | | 8.56 | | 1.24 | | 1.26 | | 0.014 | | pos |
|  | |  | | 3,3-Dimethylglutaric acid | | 159.07 | | 3.81 | | 1.39 | | 0.84 | | < 0.001 | | neg |
|  | |  | | (R)-3-Hydroxy-Octadecanoic acid | | 345.26 | | 9.04 | | 1.20 | | 0.65 | | 0.003 | | neg |
|  | |  | | 6-Ketomyristic acid | | 241.18 | | 7.68 | | 1.09 | | 0.90 | | < 0.001 | | neg |
|  | |  | | 2-Octenedioic acid | | 217.07 | | 2.24 | | 2.29 | | 3.12 | | < 0.001 | | neg |
|  | |  | | 2-hydroxyhexadecanoic acid | | 271.23 | | 9.10 | | 1.52 | | 1.11 | | < 0.001 | | neg |
|  | |  | | Nonate | | 233.10 | | 2.88 | | 1.23 | | 1.19 | | < 0.001 | | neg |
|  | |  | | 5-Tetradecenoic acid | | 271.19 | | 7.16 | | 1.18 | | 1.24 | | < 0.001 | | neg |
|  | |  | | 2-Hydroxymyristic Acid | | 243.20 | | 8.45 | | 1.11 | | 1.09 | | 0.001 | | neg |
|  | |  | | 7Z,10Z-Hexadecadienoic acid | | 297.21 | | 6.94 | | 1.08 | | 1.15 | | < 0.001 | | neg |
|  | |  | | (S)-10,16-Dihydroxyhexadecanoic acid | | 287.22 | | 7.97 | | 1.07 | | 1.11 | | 0.001 | | neg |
|  | | Fatty acid esters | | 4,8 dimethylnonanoyl carnitine | | 330.26 | | 8.46 | | 1.10 | | 0.92 | | < 0.001 | | pos |
|  | |  | | Butyl 2-decenoate | | 191.18 | | 9.22 | | 3.03 | | 1.94 | | < 0.001 | | pos |
|  | |  | | Propyl 2,4-decadienoate | | 211.17 | | 3.86 | | 1.01 | | 1.05 | | < 0.001 | | pos |
|  | |  | | Oleoylcarnitine | | 462.30 | | 8.73 | | 1.02 | | 0.89 | | 0.019 | | neg |
|  | |  | | Stearoyllactic acid | | 355.28 | | 9.88 | | 2.27 | | 2.39 | | < 0.001 | | neg |
|  | |  | | Ethyl (Z, Z)-5,8-tetradecadienoate | | 297.21 | | 6.53 | | 1.19 | | 1.14 | | < 0.001 | | neg |
|  | | Triterpenoids | | Tomentosolic acid | | 437.34 | | 9.48 | | 3.22 | | 1.60 | | < 0.001 | | pos |
|  | |  | | Ursolic acid | | 457.37 | | 8.90 | | 3.10 | | 1.54 | | < 0.001 | | pos |
|  | |  | | Camelledionol | | 423.33 | | 8.71 | | 2.98 | | 1.52 | | < 0.001 | | pos |
|  | |  | | Oleanolic acid | | 457.37 | | 9.05 | | 2.86 | | 1.66 | | < 0.001 | | pos |
|  | |  | | Sandosapogenol | | 439.36 | | 8.90 | | 2.72 | | 1.33 | | < 0.001 | | pos |
|  | |  | | (3beta,17alpha,23S,24S)-17,23-Epoxy-3,24,29-trihydroxy-27-norlanost-8-en-15-one | | 457.33 | | 7.07 | | 2.51 | | 1.50 | | < 0.001 | | pos |
|  | |  | |  | |  | |  | |  | |  | |  | |  |
| **Table S6 (continued)** | | | | | | | | | | | | | | | | |
| **HMDB Superclass** | **HMDB Subclass** | | **Metabolite** | | **M/Z** | | **Retention time** | | **VIP** | | **FC(L/S)** | | ***P*-value** | | **Mode** | |
|  | |  | | Asperagenin | | 449.33 | | 7.19 | | 1.83 | | 1.17 | | < 0.001 | | pos |
|  | |  | | Lucidenic acid E2 | | 539.26 | | 9.24 | | 1.57 | | 1.10 | | < 0.001 | | pos |
|  | |  | | Gamma-Taraxastane-3,20-diol | | 427.39 | | 10.37 | | 1.43 | | 1.11 | | 0.008 | | pos |
|  | |  | | Glyuranolide | | 530.34 | | 10.24 | | 1.32 | | 1.11 | | 0.005 | | pos |
|  | |  | | Ganoderic acid F | | 593.28 | | 9.06 | | 1.23 | | 1.06 | | 0.002 | | pos |
|  | |  | | Ganoderenic acid E | | 551.27 | | 8.85 | | 1.20 | | 1.05 | | < 0.001 | | pos |
|  | |  | | Glabric acid | | 531.33 | | 6.84 | | 2.61 | | 1.77 | | < 0.001 | | neg |
|  | |  | | Ganoderic acid A | | 497.29 | | 8.39 | | 2.58 | | 2.06 | | < 0.001 | | neg |
|  | |  | | Ganoderiol I | | 501.36 | | 9.14 | | 2.43 | | 1.37 | | < 0.001 | | neg |
|  | |  | | (3beta,19alpha)-3,19,23,24-Tetrahydroxy-12-oleanen-28-oic acid | | 539.33 | | 6.96 | | 1.69 | | 1.21 | | < 0.001 | | neg |
|  | |  | | Medicagenic acid | | 547.33 | | 7.39 | | 1.48 | | 1.32 | | 0.004 | | neg |
|  | |  | | Camellenodiol | | 463.34 | | 9.97 | | 1.16 | | 1.20 | | 0.001 | | neg |
|  | | Sesquiterpenoids | | 7(14)-Bisabolene-2,3,10,11-tetrol | | 255.19 | | 6.43 | | 1.99 | | 0.68 | | < 0.001 | | pos |
|  | |  | | Deoxynivalenol 3-glucoside | | 491.21 | | 9.33 | | 1.09 | | 0.90 | | 0.018 | | pos |
|  | |  | | S-Japonin | | 378.21 | | 9.79 | | 1.01 | | 0.91 | | 0.006 | | pos |
|  | |  | | Ipomeatetrahydrofuran | | 239.20 | | 7.87 | | 2.30 | | 1.42 | | < 0.001 | | pos |
|  | |  | | Auberganol | | 241.22 | | 8.03 | | 1.79 | | 1.26 | | < 0.001 | | pos |
|  | |  | | 4,7-Megastigmadien-9-ol | | 411.33 | | 8.63 | | 1.38 | | 1.08 | | < 0.001 | | pos |
|  | |  | | Sterebin A | | 343.25 | | 5.87 | | 1.20 | | 1.06 | | < 0.001 | | pos |
|  | |  | | (1(10)E,4a,5E)-1(10),5-Germacradiene-12-acetoxy-4,11-diol | | 295.19 | | 7.05 | | 1.77 | | 1.80 | | 0.001 | | neg |
|  | |  | | 7(14)-Farnesene-9,12-diol | | 287.22 | | 7.80 | | 1.32 | | 1.32 | | < 0.001 | | neg |
|  | |  | | Guaidiol | | 283.19 | | 6.38 | | 1.03 | | 1.13 | | 0.001 | | neg |
|  | | Terpene lactones | | Isoalantolactone | | 233.15 | | 6.28 | | 1.35 | | 0.92 | | 0.000 | | pos |
|  | |  | | 8-Deoxy-11,13-dihydroxygrosheimin | | 298.18 | | 4.86 | | 1.35 | | 0.84 | | 0.010 | | pos |
|  | |  | | Tatridin B | | 229.12 | | 6.15 | | 1.32 | | 0.90 | | < 0.001 | | pos |
|  | |  | | 2alpha-Hydroxyalantolactone | | 229.12 | | 7.92 | | 1.81 | | 0.68 | | < 0.001 | | neg |
|  | |  | | 4,11,13,15-Tetrahydroridentin B | | 249.15 | | 7.03 | | 1.52 | | 0.76 | | < 0.001 | | neg |
|  | |  | | Tavulin | | 245.12 | | 6.17 | | 1.33 | | 0.86 | | < 0.001 | | neg |
|  | |  | | Crispolide | | 261.11 | | 4.48 | | 1.24 | | 0.84 | | < 0.001 | | neg |
|  | |  | | Dihydrocumambrin A | | 307.16 | | 7.83 | | 2.94 | | 5.71 | | 0.001 | | neg |
|  | | Monoterpenoids | | Withangulatin A | | 549.25 | | 8.69 | | 2.64 | | 1.33 | | < 0.001 | | pos |
|  | |  | | Piperitone | | 170.15 | | 5.71 | | 1.70 | | 1.34 | | < 0.001 | | pos |
|  | |  | | Linalyl propionate | | 243.20 | | 5.46 | | 1.28 | | 1.25 | | 0.012 | | pos |
|  | |  | |  | |  | |  | |  | |  | |  | |  |
| **Table S6 (continued)** | | | | | | | | | | | | | | | | |
| **HMDB Superclass** | **HMDB Subclass** | | **Metabolite** | | **M/Z** | | **Retention time** | | **VIP** | | **FC(L/S)** | | ***P*-value** | | **Mode** | |
|  | |  | | 3-(5,6,6-Trimethylbicyclo [2.2.1] hept-1-yl)cyclohexanol | | 281.21 | | 7.58 | | 1.71 | | 1.43 | | < 0.001 | | neg |
|  | |  | | Valechlorin | | 457.17 | | 8.02 | | 1.63 | | 1.15 | | 0.002 | | neg |
|  | |  | | Soyasapogenol B 3-O-b-D-glucuronide | | 671.35 | | 7.95 | | 1.11 | | 1.14 | | 0.001 | | neg |
|  | | Steroid lactones | | Withaperuvin H | | 611.29 | | 8.17 | | 2.64 | | 1.45 | | 0.001 | | pos |
|  | |  | | Physagulin F | | 567.26 | | 8.21 | | 1.92 | | 1.19 | | < 0.001 | | pos |
|  | |  | | Physagulin C | | 565.24 | | 8.41 | | 1.54 | | 1.11 | | < 0.001 | | pos |
|  | |  | | Physapubenolide | | 565.25 | | 8.23 | | 1.53 | | 1.44 | | 0.001 | | neg |
|  | | Steroid lactones | | (5alpha,6beta,14alpha,20R,22R)-5,6,14,20,27-Pentahydroxy-1-oxowith-24-enolide | | 505.28 | | 5.66 | | 1.43 | | 1.20 | | < 0.001 | | neg |
|  | | Fatty alcohols | | 1-Undecanol | | 367.36 | | 10.50 | | 1.23 | | 1.47 | | 0.036 | | pos |
|  | |  | | Avocadene | | 304.29 | | 6.62 | | 1.02 | | 1.06 | | 0.012 | | pos |
|  | |  | | Avocadyne | | 329.23 | | 8.91 | | 1.14 | | 1.22 | | 0.002 | | neg |
|  | |  | | Oleyl alcohol | | 313.27 | | 9.57 | | 1.13 | | 1.10 | | < 0.001 | | neg |
|  | | Glycerophosphoethanolamines | | PE (14:1(9Z)/16:1(9Z)) | | 660.46 | | 10.33 | | 1.62 | | 0.75 | | 0.008 | | pos |
|  | |  | | LysoPE (0:0/16:0) | | 436.28 | | 8.40 | | 1.12 | | 0.87 | | 0.026 | | pos |
|  | |  | | PE (15:0/22:1(13Z)) | | 804.58 | | 11.72 | | 1.28 | | 0.81 | | 0.016 | | neg |
|  | |  | | 1-Heptadecanoylglycerophosphoethanolamine | | 512.30 | | 7.63 | | 1.09 | | 1.15 | | 0.007 | | neg |
|  | | Lineolic acids and derivatives | | Punicic acid | | 243.21 | | 7.35 | | 1.51 | | 0.85 | | < 0.001 | | pos |
|  | |  | | 13S-hydroxyoctadecadienoic acid | | 329.27 | | 9.87 | | 1.24 | | 0.89 | | < 0.001 | | pos |
|  | |  | | 2-Hydroxylinolenic acid | | 317.21 | | 7.35 | | 1.15 | | 0.90 | | < 0.001 | | pos |
|  | |  | | Cibaric acid | | 369.19 | | 6.18 | | 1.29 | | 1.20 | | < 0.001 | | neg |
|  | | Diradylglycerols | | DG (16:0/16:0/0:0) | | 591.50 | | 10.92 | | 1.20 | | 1.15 | | 0.048 | | pos |
|  | |  | | DG (20:1(11Z)/22:5(4Z,7Z,10Z,13Z,16Z)/0:0) | | 677.55 | | 9.98 | | 1.31 | | 0.78 | | 0.040 | | neg |
|  | |  | | DG (15:0/18:0/0:0) | | 627.52 | | 11.53 | | 1.21 | | 0.81 | | 0.006 | | neg |
|  | | Diterpenoids | | Beta-Tocopheryl quinone | | 433.37 | | 10.23 | | 1.60 | | 1.47 | | 0.043 | | pos |
|  | |  | | Austroinulin | | 355.28 | | 6.68 | | 1.33 | | 1.07 | | < 0.001 | | pos |
|  | |  | | Phytocassane B | | 317.21 | | 6.59 | | 1.31 | | 1.14 | | < 0.001 | | pos |
|  | | Fatty acyl glycosides | | 1-Octen-3-yl primeveroside | | 445.20 | | 7.93 | | 1.75 | | 0.80 | | 0.015 | | pos |
|  | |  | | 4-Hydroxyproline galactoside | | 258.10 | | 3.23 | | 1.51 | | 0.86 | | < 0.001 | | pos |
|  | |  | | 6S,9R-Dihydroxy-4,7E-megastigmadien-3-one 9-[apiosyl-(1->6)-glucoside] | | 563.23 | | 8.41 | | 1.40 | | 1.16 | | < 0.001 | | neg |
|  | | Bile acids, alcohols and derivatives | | 5beta-Cholestane-3alpha,7alpha,24,26-tetrol | | 401.34 | | 7.36 | | 1.14 | | 0.92 | | 0.008 | | pos |
|  | |  | | 27-Nor-5b-cholestane-3a,7a,12a,24,25-pentol | | 480.37 | | 9.34 | | 1.14 | | 0.90 | | 0.012 | | pos |
|  | |  | |  | |  | |  | |  | |  | |  | |  |
| **Table S6 (continued)** | | | | | | | | | | | | | | | | |
| **HMDB Superclass** | **HMDB Subclass** | | **Metabolite** | | **M/Z** | | **Retention time** | | **VIP** | | **FC(L/S)** | | ***P*-value** | | **Mode** | |
|  | | Cholestane steroids | | 5a-Cholest-8-en-3b-ol | | 409.35 | | 10.61 | | 1.27 | | 0.86 | | 0.003 | | pos |
|  | |  | | 5alpha-Cholestanone | | 811.68 | | 11.16 | | 1.22 | | 1.20 | | 0.014 | | pos |
|  | | Glycerophosphates | | LysoPA(P-16:0e/0:0) | | 417.24 | | 9.12 | | 1.55 | | 1.25 | | 0.001 | | pos |
|  | |  | | 1-(9Z-tetradecenoyl)-glycero-3-phosphate | | 379.19 | | 8.11 | | 1.80 | | 0.56 | | < 0.001 | | neg |
|  | | Glycerophosphocholines | | PC (16:0/18:2(9Z,12Z)) | | 802.56 | | 11.16 | | 1.25 | | 0.86 | | 0.008 | | neg |
|  | |  | | LysoPC (16:0) | | 540.33 | | 8.36 | | 1.06 | | 0.88 | | 0.011 | | neg |
|  | | Hydroxysteroids | | Tetrahydrodeoxycorticosterone | | 379.22 | | 8.98 | | 1.47 | | 1.15 | | < 0.001 | | pos |
|  | |  | | Prednisone | | 379.15 | | 8.92 | | 1.36 | | 0.83 | | 0.001 | | neg |
|  | | Pregnane steroids | | 17a-Hydroxypregnenolone | | 333.24 | | 5.75 | | 3.02 | | 1.80 | | < 0.001 | | pos |
|  | |  | | Halobetasol Propionate | | 521.13 | | 7.58 | | 2.13 | | 1.72 | | 0.001 | | neg |
|  | | Steroidal glycosides | | Corchoroside B | | 541.28 | | 9.01 | | 1.64 | | 1.14 | | < 0.001 | | pos |
|  | |  | | Torvoside D | | 747.39 | | 7.73 | | 2.24 | | 1.67 | | < 0.001 | | neg |
|  | | Terpene glycosides | | Lansioside A | | 642.44 | | 11.64 | | 1.24 | | 1.14 | | 0.019 | | pos |
|  | |  | | Calenduloside E | | 677.39 | | 7.77 | | 2.83 | | 2.77 | | < 0.001 | | neg |
|  | | Ceramides | | N-[(4E,8E)-1,3-dihydroxyoctadeca-4,8-dien-2-yl]hexadecanamide | | 580.49 | | 11.88 | | 1.15 | | 0.86 | | 0.018 | | neg |
|  | | Eicosanoids | | Carboprost Tromethamine | | 367.25 | | 7.62 | | 1.06 | | 0.87 | | < 0.001 | | neg |
|  | | Ergostane steroids | | Delta 8,14 -Sterol | | 393.35 | | 12.76 | | 1.38 | | 0.88 | | < 0.001 | | pos |
|  | | Estrane steroids | | 4-hydroxyestradiol | | 289.18 | | 5.25 | | 1.15 | | 1.08 | | < 0.001 | | pos |
|  | | Monoradylglycerols | | MG(i-12:0/0:0/0:0) | | 255.20 | | 8.07 | | 1.46 | | 1.17 | | < 0.001 | | neg |
|  | | Quinone and hydroquinone lipids | | 13'-Carboxy-alpha-tocopherol | | 493.39 | | 11.52 | | 1.62 | | 0.71 | | 0.001 | | pos |
|  | | Steroid esters | | Physapubescin | | 553.28 | | 7.95 | | 2.09 | | 1.22 | | < 0.001 | | pos |
|  | | Stigmastanes and derivatives | | 6-Deoxohomodolichosterone | | 463.38 | | 10.59 | | 1.63 | | 1.17 | | 0.003 | | pos |
| Organic acids and derivatives | | Amino acids, peptides, and analogues | | N6-Acetyl-5S-hydroxy-L-lysine | | 243.08 | | 4.68 | | 2.85 | | 0.37 | | < 0.001 | | pos |
|  |  |  | | Cytidine 2',3'-cyclic phosphate | | 350.01 | | 5.50 | | 1.68 | | 0.75 | | 0.014 | | pos |
|  |  |  | | Valyl-Glutamate | | 229.12 | | 1.00 | | 1.63 | | 0.86 | | < 0.001 | | pos |
|  | |  | | Frangulanine | | 483.34 | | 9.62 | | 1.55 | | 0.75 | | 0.010 | | pos |
|  | |  | | Isoleucyl-Glutamate | | 243.13 | | 1.41 | | 1.44 | | 0.90 | | < 0.001 | | pos |
|  | |  | | Tetrahydrodipicolinate | | 204.09 | | 1.57 | | 1.24 | | 0.88 | | < 0.001 | | pos |
|  | |  | | Phenylalanylproline | | 245.13 | | 3.54 | | 1.07 | | 0.93 | | < 0.001 | | pos |
|  | |  | | N-[[3-Hydroxy-2-(2-pentenyl) cyclopentyl] acetyl] isoleucine | | 308.22 | | 7.67 | | 2.39 | | 1.41 | | < 0.001 | | pos |
|  | |  | | 5-Hydroxyindoleacetylglycine | | 213.07 | | 3.25 | | 1.61 | | 1.24 | | < 0.001 | | pos |
|  | |  | |  | |  | |  | |  | |  | |  | |  |
| **Table S6 (continued)** | | | | | | | | | | | | | | | | |
| **HMDB Superclass** | **HMDB Subclass** | | **Metabolite** | | **M/Z** | | **Retention time** | | **VIP** | | **FC(L/S)** | | ***P*-value** | | **Mode** | |
|  | |  | | 2-Hepteneoylglycine | | 230.10 | | 3.07 | | 2.04 | | 0.43 | | < 0.001 | | neg |
|  | |  | | (1R)-Glutathionyl-(2R)-hydroxy-1,2-dihydronaphthalene | | 472.12 | | 5.47 | | 1.76 | | 0.55 | | 0.001 | | neg |
|  | |  | | N-(4-Hydroxycinnamoyl) tyrosine | | 362.08 | | 5.41 | | 1.73 | | 0.74 | | < 0.001 | | neg |
|  | |  | | Captopril-cysteine disulfide | | 371.05 | | 8.00 | | 1.61 | | 0.48 | | < 0.001 | | neg |
|  | |  | | Indolylacryloylglycine | | 225.07 | | 3.35 | | 1.47 | | 0.89 | | < 0.001 | | neg |
|  | |  | | N-Arachidonoyl glycine | | 406.26 | | 8.05 | | 1.25 | | 0.77 | | < 0.001 | | neg |
|  | |  | | 3-Hydroxymugineic acid | | 357.09 | | 5.55 | | 1.24 | | 0.80 | | 0.034 | | neg |
|  | |  | | Arginyl-Gamma-glutamate | | 283.15 | | 7.15 | | 1.16 | | 0.80 | | 0.001 | | neg |
|  | |  | | 4-Hydroxystachydrine | | 158.08 | | 3.22 | | 1.19 | | 1.31 | | 0.039 | | neg |
|  | |  | | Oleoyl glycine | | 384.27 | | 8.85 | | 1.08 | | 1.20 | | 0.003 | | neg |
|  | |  | | N-Acetylproline | | 138.06 | | 2.85 | | 1.08 | | 1.22 | | 0.003 | | neg |
|  | | Hybrid peptides | | Beta-Alanyl-L-lysine | | 476.32 | | 8.31 | | 1.48 | | 0.86 | | < 0.001 | | pos |
|  | |  | | Astin I | | 516.20 | | 2.93 | | 1.17 | | 1.10 | | 0.002 | | pos |
|  | | Medium-chain hydroxy acids and derivatives | | 12-Hydroxydodecanoic acid | | 258.21 | | 5.72 | | 1.34 | | 0.88 | | < 0.001 | | pos |
| Organoheterocyclic compounds | | Gamma butyrolactones | | 3-Hydroxyadipic acid 3,6-lactone | | 143.04 | | 1.62 | | 1.58 | | 0.61 | | 0.001 | | neg |
|  | |  | | Artabsinolide A | | 261.11 | | 6.13 | | 1.32 | | 0.69 | | 0.013 | | neg |
|  | |  | | 5-Nonyltetrahydro-2-oxo-3-furancarboxylic acid | | 237.15 | | 6.26 | | 1.17 | | 1.14 | | < 0.001 | | neg |
|  | | 1-benzopyrans | | Gamma-CEHC | | 263.13 | | 5.26 | | 1.33 | | 0.76 | | 0.001 | | neg |
|  | |  | | 9'-Carboxy-gamma-chromanol | | 421.26 | | 7.53 | | 1.22 | | 1.20 | | 0.000 | | neg |
|  | | Hydropyridines | | 5-(2-Furanyl)-1,2,3,4,5,6-hexahydro-7H-cyclopenta[b]pyridin-7-one | | 248.09 | | 3.96 | | 1.10 | | 0.75 | | 0.015 | | neg |
|  | |  | | 2-Hydroxypyridine | | 140.04 | | 2.51 | | 1.64 | | 1.69 | | < 0.001 | | neg |
|  | | Indolyl carboxylic acids and derivatives | | Indoleacetic acid | | 220.06 | | 3.70 | | 1.79 | | 0.46 | | 0.002 | | neg |
|  | |  | | 5-Hydroxyindoleacetic acid | | 190.05 | | 3.53 | | 1.14 | | 0.77 | | 0.029 | | neg |
|  | | Benzoxazinones | | (R)-2,7-Dihydroxy-2H-1,4-benzoxazin-3(4H)-one | | 380.11 | | 5.58 | | 1.76 | | 0.81 | | < 0.001 | | pos |
|  | | Benzylisoquinolines | | 8-Propanoylneosolaniol | | 473.16 | | 7.24 | | 1.71 | | 0.68 | | 0.018 | | neg |
|  | | Bilirubins | | D-Urobilin | | 609.27 | | 8.19 | | 2.50 | | 2.26 | | 0.002 | | neg |
|  | | Dibenzoxepines | | Doxepin N-oxide glucuronide | | 507.17 | | 7.28 | | 2.28 | | 2.29 | | 0.016 | | neg |
|  | | Indoles | | Indoleacrylic acid | | 188.07 | | 3.41 | | 1.46 | | 0.85 | | 0.000 | | pos |
|  | |  | |  | |  | |  | |  | |  | |  | |  |
| **Table S6 (continued)** | | | | | | | | | | | | | | | | |
| **HMDB Superclass** | **HMDB Subclass** | | **Metabolite** | | **M/Z** | | **Retention time** | | **VIP** | | **FC(L/S)** | | ***P*-value** | | **Mode** | |
|  | | Pterins and derivatives | | 2-Amino-4-oxo-6-(1',2'-dioxoprolyl)-7,8-dihydroxypteridine | | 288.03 | | 1.24 | | 1.16 | | 0.87 | | < 0.001 | | neg |
|  | | Pyranones and derivatives | | Erinapyrone A | | 187.06 | | 2.69 | | 1.27 | | 1.20 | | < 0.001 | | neg |
|  | | Unclassified | | Canescein | | 611.25 | | 8.45 | | 2.75 | | 1.76 | | 0.001 | | pos |
|  | |  | | (+)-2,3-Dihydro-3-methyl-1H-pyrrole | | 125.11 | | 1.14 | | 1.27 | | 1.10 | | 0.022 | | pos |
|  | |  | | 3alpha-Hydroxyoreadone | | 285.17 | | 3.39 | | 1.00 | | 1.05 | | < 0.001 | | pos |
|  | |  | | N-Carbamoyl glucuronide lorcaserin | | 454.17 | | 8.36 | | 1.28 | | 0.78 | | 0.003 | | neg |
|  | |  | | Jasmine ketolactone | | 253.11 | | 5.43 | | 1.40 | | 1.37 | | < 0.001 | | neg |
|  | |  | | 3-Pyridinebutanoic acid | | 210.08 | | 4.75 | | 1.28 | | 1.26 | | < 0.001 | | neg |
| Organic oxygen compounds | | Carbohydrates and carbohydrate conjugates | | Indican | | 296.11 | | 2.97 | | 2.76 | | 0.69 | | < 0.001 | | pos |
|  | |  | | N-Acetylneuraminic acid | | 274.09 | | 2.80 | | 1.90 | | 0.68 | | 0.001 | | pos |
|  | |  | | (2S)-2-Butanol O-[b-D-Apiofuranosyl-(1->6)-b-D-glucopyranoside] | | 410.20 | | 8.36 | | 1.18 | | 0.91 | | < 0.001 | | pos |
|  | |  | | Ethyl glucuronide | | 187.06 | | 3.10 | | 1.01 | | 0.92 | | 0.002 | | pos |
|  | |  | | 6-(5-ethyl-2,3-dihydroxyphenoxy)-3,4,5-trihydroxyoxane-2-carboxylic acid | | 295.08 | | 3.42 | | 2.02 | | 1.52 | | 0.001 | | pos |
|  | |  | | 4-Methoxybenzyl O-(2-sulfoglucoside) | | 345.06 | | 6.38 | | 1.57 | | 1.48 | | 0.022 | | pos |
|  | |  | | Arbutin | | 317.06 | | 9.96 | | 1.42 | | 1.13 | | 0.000 | | pos |
|  | |  | | 3,4,5-trihydroxy-6-[(4-methoxy-1-benzofuran-6-yl) oxy] oxane-2-carboxylic acid | | 363.07 | | 6.81 | | 1.39 | | 1.23 | | 0.048 | | pos |
|  | |  | | Dihydromaleimide beta-D-glucoside | | 260.08 | | 1.40 | | 2.87 | | 0.25 | | < 0.001 | | neg |
|  | |  | | Tyramine glucuronide | | 294.10 | | 2.97 | | 2.32 | | 0.71 | | < 0.001 | | neg |
|  | |  | | Pisatoside | | 260.08 | | 1.63 | | 2.22 | | 0.61 | | < 0.001 | | neg |
|  | |  | | Fluoxetine glucuronide | | 520.14 | | 7.36 | | 2.14 | | 0.44 | | < 0.001 | | neg |
|  | |  | | 3-oxo-3-[(3,4,5,6-tetrahydroxyoxan-2-yl) methoxy] propanoic acid | | 247.05 | | 2.90 | | 1.57 | | 0.76 | | < 0.001 | | neg |
|  | |  | | 6-Sinapoylglucoraphenin | | 640.08 | | 0.83 | | 1.21 | | 0.91 | | < 0.001 | | neg |
|  | | Carbonyl compounds | | 3-Propyl-1,2-cyclopentanedione | | 182.12 | | 4.51 | | 1.66 | | 0.57 | | 0.032 | | pos |
|  | |  | | 1H-Pyrrole-2-carboxaldehyde | | 96.04 | | 0.71 | | 1.29 | | 1.11 | | < 0.001 | | pos |
|  | |  | | (3R,8E)-3-Hydroxy-5,8-megastigmadien-7-one | | 209.15 | | 4.35 | | 1.02 | | 1.06 | | 0.002 | | pos |
|  | |  | | 1-Hydroxyepiacorone | | 289.12 | | 3.60 | | 1.00 | | 0.85 | | 0.007 | | neg |
|  | |  | | 3-Methylcyclopentadecanone | | 283.23 | | 8.91 | | 1.15 | | 1.16 | | 0.001 | | neg |
|  | | Ethers | | Digoxigenin monodigitoxoside | | 565.28 | | 8.94 | | 1.42 | | 1.09 | | < 0.001 | | pos |
|  | |  | |  | |  | |  | |  | |  | |  | |  |
| **Table S6 (continued)** | | | | | | | | | | | | | | | | |
| **HMDB Superclass** | **HMDB Subclass** | | **Metabolite** | | **M/Z** | | **Retention time** | | **VIP** | | **FC(L/S)** | | ***P*-value** | | **Mode** | |
| Phenylpropanoids and polyketides | | Furanocoumarins | | (R)-Heraclenol 2'-(3-methyl-2-butenoate) | | 385.13 | | 7.70 | | 1.06 | | 0.76 | | 0.016 | | neg |
|  | |  | | Edulisin I | | 525.15 | | 8.03 | | 1.43 | | 1.21 | | 0.004 | | neg |
|  | | Cyclic diarylheptanoids | | Myricatomentoside II | | 571.16 | | 8.02 | | 1.56 | | 1.31 | | 0.003 | | neg |
|  | | Flavans | | 2-phenyl-3,4-dihydro-2H-1-benzopyran-3,5,7-triol | | 259.10 | | 3.88 | | 1.48 | | 0.76 | | 0.007 | | pos |
|  | | Flavonoid glycosides | | Delphinidin 3-rutinoside | | 594.16 | | 7.01 | | 1.24 | | 0.83 | | 0.001 | | pos |
|  | | Pyranoflavonoids | | Cycloartocarpin | | 479.17 | | 7.66 | | 1.90 | | 1.49 | | < 0.001 | | neg |
|  | | Unclassified | | 3-(1,2-dihydroxybutyl)-7-hydroxy-1H-isochromen-1-one | | 268.12 | | 4.98 | | 1.30 | | 0.68 | | 0.049 | | pos |
|  | |  | | Coriandrone C | | 264.09 | | 3.02 | | 1.21 | | 0.90 | | < 0.001 | | pos |
|  | |  | | Yuzu lactone | | 197.15 | | 6.77 | | 1.20 | | 0.90 | | < 0.001 | | pos |
|  | |  | | Alpha-methylphenylalanine | | 180.10 | | 3.65 | | 1.15 | | 0.92 | | < 0.001 | | pos |
|  | |  | | 3-(3,4-Dihydroxyphenyl)-2-methylpropionic acid | | 195.07 | | 5.42 | | 1.36 | | 0.87 | | < 0.001 | | neg |
|  | |  | | Norpropoxyphene | | 370.20 | | 7.44 | | 1.90 | | 1.92 | | 0.003 | | neg |
|  | |  | | 6-[(1Z)-2-hydroxy-3-oxobut-1-en-1-yl]-7-methoxy-2H-chromen-2-one | | 305.07 | | 4.32 | | 1.68 | | 1.32 | | 0.002 | | neg |
| Benzenoids | | Lineolic acids and derivatives | | Antibiotic SB 202742 | | 393.24 | | 9.47 | | 1.92 | | 1.49 | | < 0.001 | | pos |
|  | |  | | 3,5-Bis(1,1-dimethylethyl)-4-hydroxy-benzoic acid ethyl ester | | 573.34 | | 8.00 | | 1.67 | | 1.69 | | 0.001 | | neg |
|  | |  | | Salicylic acid | | 137.02 | | 4.65 | | 1.07 | | 1.06 | | < 0.001 | | neg |
|  | | Benzenediols | | (Z, Z)-2-Methyl-5-(8,11,14-pentadecatrienyl)-1,3-benzenediol | | 392.25 | | 7.10 | | 1.17 | | 1.12 | | 0.002 | | pos |
|  | |  | | 3-(3,4-dihydroxyphenyl)-N-[2-(4-hydroxyphenyl) ethyl] propanimidic acid | | 338.08 | | 1.28 | | 1.01 | | 0.89 | | < 0.001 | | neg |
|  | | Indanones | | Pterosin N | | 279.12 | | 3.41 | | 1.03 | | 0.83 | | 0.011 | | neg |
|  | | Unclassified | | 1-Phenylethanol | | 121.07 | | 4.20 | | 1.78 | | 1.38 | | < 0.001 | | neg |
| Organosulfur compounds | | Aryl thioethers | | Phenyl vinyl sulfide | | 181.03 | | 6.43 | | 1.55 | | 0.56 | | 0.015 | | neg |
| Nucleosides, nucleotides, and analogues | | Unclassified | | 1-Methylinosine | | 606.22 | | 10.46 | | 1.11 | | 1.16 | | 0.020 | | pos |
| Alkaloids and derivatives | | Unclassified | | Harmalol | | 181.08 | | 3.35 | | 1.32 | | 0.89 | | < 0.001 | | neg |
|  | |  | |  | |  | |  | |  | |  | |  | |  |
| **Table S6 (continued)** | | | | | | | | | | | | | | | | |
| **HMDB Superclass** | **HMDB Subclass** | | **Metabolite** | | **M/Z** | | **Retention time** | | **VIP** | | **FC(L/S)** | | ***P*-value** | | **Mode** | |
| Others | |  | | 6-hydroxysphingosine | | 316.28 | | 5.81 | | 2.55 | | 0.70 | | < 0.001 | | pos |
|  | |  | | 8-Isoquinoline methanamine (hydrochloride) | | 158.10 | | 3.33 | | 2.38 | | 0.73 | | < 0.001 | | pos |
|  | |  | | Docosahexaenoyl Glycine | | 386.27 | | 9.00 | | 2.20 | | 0.53 | | < 0.001 | | pos |
|  | |  | | NORELEAGNINE | | 173.11 | | 3.22 | | 2.09 | | 0.58 | | 0.002 | | pos |
|  | |  | | Hydroxy-gamma-sanshool | | 290.21 | | 4.42 | | 1.71 | | 0.78 | | 0.014 | | pos |
|  | |  | | Cis-5-dodecenoic acid | | 199.17 | | 6.74 | | 1.57 | | 0.84 | | < 0.001 | | pos |
|  | |  | | Quadrone | | 249.15 | | 6.22 | | 1.51 | | 0.84 | | < 0.001 | | pos |
|  | |  | | (±)-Equol | | 243.10 | | 5.11 | | 1.41 | | 0.89 | | < 0.001 | | pos |
|  | |  | | 2,3,5-Trimethacarb | | 194.12 | | 3.51 | | 1.38 | | 0.90 | | < 0.001 | | pos |
|  | |  | | 9S,10R-Epoxy-6Z-octadecene | | 570.54 | | 12.18 | | 1.29 | | 0.92 | | 0.001 | | pos |
|  | |  | | 12-SAHSA | | 589.52 | | 13.60 | | 1.27 | | 0.93 | | 0.003 | | pos |
|  | |  | | PE (16:1(5Z)/16:1(5Z)) | | 688.49 | | 10.81 | | 1.26 | | 0.91 | | 0.007 | | pos |
|  | |  | | Cer(d14:2(4E,6E)/16:0) | | 480.44 | | 10.40 | | 1.23 | | 0.91 | | 0.004 | | pos |
|  | |  | | Confertifoline | | 235.17 | | 6.64 | | 1.21 | | 0.92 | | < 0.001 | | pos |
|  | |  | | (+)-Prosopinine | | 314.27 | | 4.51 | | 1.12 | | 0.93 | | < 0.001 | | pos |
|  | |  | | 1-Palmitoyl-2-linoleoyl PE | | 716.52 | | 11.28 | | 1.11 | | 0.94 | | 0.008 | | pos |
|  | |  | | Bifemelane (M5) | | 287.13 | | 5.24 | | 1.08 | | 0.83 | | 0.020 | | pos |
|  | |  | | 15(R)-15-methyl Prostaglandin A2 | | 349.24 | | 4.87 | | 3.30 | | 2.11 | | < 0.001 | | pos |
|  | |  | | (3beta,5xi,9xi,18xi)-Olean-12-en-28-oic acid,3-hydroxy- | | 439.36 | | 9.22 | | 3.05 | | 1.43 | | < 0.001 | | pos |
|  | |  | | Fenirofibrate | | 321.09 | | 4.08 | | 2.51 | | 2.60 | | 0.017 | | pos |
|  | |  | | 2-aminohexadecanoic acid | | 272.26 | | 7.49 | | 2.12 | | 1.48 | | < 0.001 | | pos |
|  | |  | | 3,5,7-Trimethyl-2E,4E,6E,8E-decatetraene | | 177.16 | | 8.85 | | 1.79 | | 1.20 | | < 0.001 | | pos |
|  | |  | | 2-oxo-pentadecanoic acid | | 239.20 | | 8.05 | | 1.77 | | 1.19 | | < 0.001 | | pos |
|  | |  | | Amprotropine | | 308.22 | | 4.74 | | 1.70 | | 1.15 | | < 0.001 | | pos |
|  | |  | | 16,16-dimethyl-PGE1 | | 383.28 | | 7.32 | | 1.69 | | 1.20 | | < 0.001 | | pos |
|  | |  | | 1alpha,25-dihydroxy-26,27-ethanovitamin D3 | | 443.35 | | 8.68 | | 1.68 | | 1.13 | | < 0.001 | | pos |
|  | |  | | Lumichrome | | 526.18 | | 4.17 | | 1.67 | | 1.20 | | < 0.001 | | pos |
|  | |  | | Oleoyl dopamine | | 418.33 | | 8.58 | | 1.67 | | 1.19 | | 0.002 | | pos |
|  | |  | | 7alpha,12alpha,24-trihydroxycholest-4-en-3-one | | 433.33 | | 7.78 | | 1.54 | | 1.18 | | < 0.001 | | pos |
|  | |  | | Pfaffic acid | | 441.34 | | 9.13 | | 1.52 | | 1.09 | | < 0.001 | | pos |
|  | |  | | Vitamin D2 (Ergocalciferol) | | 397.35 | | 9.13 | | 1.51 | | 1.11 | | < 0.001 | | pos |
|  | |  | | 4-formyl Indole | | 146.06 | | 2.98 | | 1.48 | | 1.22 | | 0.001 | | pos |
|  | |  | | Malvidin | | 331.08 | | 5.08 | | 1.46 | | 1.18 | | 0.008 | | pos |
|  | |  | |  | |  | |  | |  | |  | |  | |  |
| **Table S6 (continued)** | | | | | | | | | | | | | | | | |
| **HMDB Superclass** | **HMDB Subclass** | | **Metabolite** | | **M/Z** | | **Retention time** | | **VIP** | | **FC(L/S)** | | ***P*-value** | | **Mode** | |
|  | |  | | PG(16:0/0:0)[U] | | 467.28 | | 10.18 | | 1.42 | | 1.08 | | 0.001 | | pos |
|  | |  | | Alpha-Vetivone | | 219.17 | | 10.05 | | 1.40 | | 1.15 | | 0.049 | | pos |
|  | |  | | Alpha-9(10)-EpODE | | 295.23 | | 6.49 | | 1.38 | | 1.08 | | < 0.001 | | pos |
|  | |  | | Nuatigenin | | 431.32 | | 8.03 | | 1.38 | | 1.11 | | < 0.001 | | pos |
|  | |  | | 9-hydroxy-10-oxo-12(Z)-octadecenoic acid | | 335.22 | | 6.68 | | 1.35 | | 1.05 | | < 0.001 | | pos |
|  | |  | | 1-ACETYLPIPERIDINE | | 128.11 | | 3.56 | | 1.35 | | 1.19 | | 0.040 | | pos |
|  | |  | | (±)12,13-DiHOME | | 337.23 | | 6.76 | | 1.34 | | 1.06 | | < 0.001 | | pos |
|  | |  | | Coumestrol | | 269.04 | | 5.34 | | 1.33 | | 1.18 | | 0.001 | | pos |
|  | |  | | Orcinol | | 125.06 | | 2.67 | | 1.28 | | 1.12 | | < 0.001 | | pos |
|  | |  | | Tetranor-12(R)-HETE | | 267.19 | | 5.89 | | 1.25 | | 1.08 | | < 0.001 | | pos |
|  | |  | | R-Palmitoyl-(2-methyl) Ethanolamide | | 314.30 | | 7.48 | | 1.17 | | 1.09 | | 0.008 | | pos |
|  | |  | | Stigmasta-5,7-dien-3beta-ol | | 413.38 | | 10.27 | | 1.17 | | 1.12 | | 0.009 | | pos |
|  | |  | | 9,10-epoxy-13-hydroxy-11-octadecenoic acid | | 313.24 | | 6.49 | | 1.15 | | 1.04 | | < 0.001 | | pos |
|  | |  | | TG (12:0/13:0/20:5(5Z,8Z,11Z,14Z,17Z))[iso6] | | 755.61 | | 11.02 | | 1.06 | | 1.16 | | 0.021 | | pos |
|  | |  | | 7S,8S-DiHOTrE | | 311.22 | | 5.61 | | 1.04 | | 1.04 | | < 0.001 | | pos |
|  | |  | | Glycerophospho-N-Palmitoyl Ethanolamine | | 454.29 | | 9.49 | | 1.01 | | 1.07 | | 0.001 | | pos |
|  | |  | | PE (16:0/0:0) | | 454.29 | | 8.59 | | 1.01 | | 1.05 | | 0.004 | | pos |
|  | |  | | PG (17:1(9Z)/0:0) | | 497.29 | | 10.39 | | 1.01 | | 1.07 | | 0.008 | | pos |
|  | |  | | SIMMONDSIN-2'-FERULATE | | 550.19 | | 8.11 | | 1.11 | | 0.89 | | 0.001 | | neg |
|  | |  | | P-Salicylic acid | | 137.02 | | 4.20 | | 1.17 | | 1.08 | | < 0.001 | | neg |
|  | |  | | MEDICA 16 | | 341.27 | | 8.94 | | 1.17 | | 1.09 | | < 0.001 | | neg |
| Diets: S, steam-flaked corn and corn silage diet; L, sugar beet pulp and alfalfa silage diet; HMDB, human metabolome database; M/Z, mass-to-charge ratio; FC, fold change; VIP, variable importance in the projection; pos, in positive ion mode; neg, in negative ion mode. | | | | | | | | | | | | | | | | |

| **Table S7 Significantly differential metabolites between the glucogenic diet C and S after 48 h *in vitro* fermentation with rumen fluid of dairy cows** | | | | | | | | | | | | | | | | |
| --- | --- | --- | --- | --- | --- | --- | --- | --- | --- | --- | --- | --- | --- | --- | --- | --- |
| **HMDB Superclass** | **HMDB Subclass** | | **Metabolite** | | | **M/Z** | | **Retention time** | | **VIP** | | **FC(C/S)** | | ***P*-value** | **Mode** | |
| Lipids and lipid-like molecules | | Fatty acids and conjugates | | Ethylmalonic acid | 131.04 | | 0.78 | | 1.10 | | 0.96 | | 0.034 | | | neg |
|  | |  | | 11Z-Eicosenoic acid | 355.28 | | 9.19 | | 1.12 | | 1.03 | | 0.007 | | | neg |
|  | |  | | Docosatrienoic acid | 357.28 | | 8.56 | | 2.52 | | 1.26 | | 0.034 | | | pos |
|  | |  | | Elaidic Acid | 283.26 | | 8.50 | | 1.34 | | 1.02 | | 0.005 | | | pos |
|  | |  | | Floionolic acid | 315.25 | | 9.73 | | 1.19 | | 1.02 | | 0.045 | | | pos |
|  | |  | | 5-Hexyl-2-furanhexanoic acid | 289.18 | | 5.85 | | 1.03 | | 1.02 | | 0.026 | | | pos |
|  | | Triterpenoids | | Camellenodiol | 463.34 | | 9.97 | | 2.30 | | 1.16 | | < 0.001 | | | neg |
|  | |  | | Ganoderic acid beta | 545.31 | | 8.51 | | 1.27 | | 1.02 | | 0.017 | | | neg |
|  | |  | | Lucidenic acid E2 | 539.26 | | 9.24 | | 1.33 | | 1.02 | | 0.003 | | | pos |
|  | |  | | Medicoside C | 461.25 | | 7.59 | | 1.13 | | 1.02 | | 0.010 | | | pos |
|  | | Glycerophosphates | | 1-(9Z-tetradecenoyl)-glycero-3-phosphate | 379.19 | | 8.11 | | 2.13 | | 0.84 | | 0.007 | | | neg |
|  | |  | | 1-tetradecanoyl-sn-glycero-3-phosphate | 365.21 | | 8.67 | | 2.45 | | 0.89 | | 0.005 | | | pos |
|  | |  | | LysoPA(P-16:0e/0:0) | 417.24 | | 9.12 | | 2.56 | | 1.19 | | 0.021 | | | pos |
|  | | Monoterpenoids | | (1beta,2beta,5beta) -p-Menth-3-ene-1,2,5-triol | 231.12 | | 4.67 | | 1.04 | | 0.97 | | 0.004 | | | neg |
|  | |  | | Piperitol | 155.14 | | 6.10 | | 3.11 | | 1.54 | | 0.016 | | | pos |
|  | |  | | Alpha-Terpineol propanoate | 211.17 | | 7.24 | | 1.59 | | 1.10 | | 0.037 | | | pos |
|  | | Sesquiterpenoids | | (1(10)E,4a,5E)-1(10),5-Germacradiene-12-acetoxy-4,11-diol | 295.19 | | 7.05 | | 2.40 | | 1.45 | | 0.043 | | | neg |
|  | |  | | 7(14)-Bisabolene-2,3,10,11-tetrol | 255.19 | | 6.43 | | 1.52 | | 1.06 | | 0.018 | | | pos |
|  | |  | | S-Japonin | 378.21 | | 9.79 | | 1.23 | | 0.96 | | 0.049 | | | pos |
|  | | Ergostane steroids | | (24R)-Ergost-4-ene-3,6-dione | 457.33 | | 9.53 | | 1.27 | | 1.06 | | 0.014 | | | neg |
|  | |  | | Delta 8,14 -Sterol | 393.35 | | 12.76 | | 1.16 | | 0.97 | | 0.049 | | | pos |
|  | | Glycerophosphoethanolamines | | PE (14:1(9Z)/16:1(9Z)) | 660.46 | | 10.33 | | 2.56 | | 1.17 | | 0.040 | | | pos |
|  | |  | | LysoPE (0:0/16:0) | 436.28 | | 8.40 | | 1.85 | | 1.09 | | 0.009 | | | pos |
|  | | Diterpenoids | | (13R,14R)-7-Labdene-13,14,15-triol | 357.30 | | 8.36 | | 1.39 | | 1.02 | | 0.001 | | | pos |
|  | | Eicosanoids | | Carboprost Tromethamine | 367.25 | | 7.62 | | 1.57 | | 1.07 | | 0.011 | | | neg |
|  | | Fatty acyl glycosides | | 4-Hydroxyproline galactoside | 258.10 | | 3.23 | | 2.07 | | 1.08 | | 0.002 | | | pos |
|  | | Fatty alcohols | | 13-Heptadecyn-1-ol | 253.25 | | 8.48 | | 1.21 | | 1.03 | | 0.042 | | | pos |
|  | | Fatty amides | | Palmitoyl Serinol | 330.30 | | 8.94 | | 3.04 | | 0.80 | | 0.004 | | | pos |
|  | | Steroid esters | | Physapubescin | 553.28 | | 7.95 | | 1.45 | | 1.04 | | 0.026 | | | pos |
|  | |  | |  |  | |  | |  | |  | |  | | |  |
| **Table S7 (continued)** | | | | | | | | | | | | | | | | |
| **HMDB Superclass** | **HMDB Subclass** | | **Metabolite** | | | **M/Z** | | **Retention time** | | **VIP** | | **FC(C/S)** | | ***P*-value** | **Mode** | |
|  | | Steroid lactones | | Physagulin C | 565.24 | | 8.41 | | 1.41 | | 1.03 | | 0.040 | | | pos |
|  | | Terpene lactones | | Isoalantolactone | 233.15 | | 6.28 | | 1.04 | | 1.02 | | 0.040 | | | pos |
|  | | Unclassified | | 11,13-Dihydrotaraxinic acid glucosyl ester | 407.17 | | 7.10 | | 3.30 | | 0.54 | | 0.029 | | | neg |
| Organic acids and derivatives | | Amino acids, peptides, and analogues | | Captopril-cysteine disulfide | 371.05 | | 8.00 | | 2.38 | | 1.30 | | 0.014 | | | neg |
|  | |  | | 2-Hepteneoylglycine | 230.10 | | 3.07 | | 2.66 | | 1.22 | | < 0.001 | | | neg |
|  | |  | | Tetrahydrodipicolinate | 204.09 | | 1.57 | | 2.29 | | 1.11 | | < 0.001 | | | pos |
|  | |  | | Valyl-Proline | 197.13 | | 3.02 | | 1.90 | | 1.09 | | 0.008 | | | pos |
|  | |  | | Isoleucyl-Glutamate | 243.13 | | 1.41 | | 1.85 | | 1.05 | | 0.004 | | | pos |
|  | |  | | Dynorphin B (10-13) | 459.30 | | 3.72 | | 1.76 | | 0.93 | | 0.028 | | | pos |
|  | |  | | 5-Hydroxyindoleacetylglycine | 213.07 | | 3.25 | | 1.65 | | 1.10 | | 0.033 | | | pos |
|  | |  | | Glutamylleucine | 243.13 | | 0.76 | | 1.44 | | 1.03 | | 0.002 | | | pos |
|  | |  | | Alanyl-Phenylalanine | 219.11 | | 2.68 | | 1.38 | | 1.07 | | 0.039 | | | pos |
|  | |  | | 5-hydroxyhexanoylglycine | 212.09 | | 0.75 | | 1.36 | | 1.03 | | 0.003 | | | pos |
|  | |  | | D-Pipecolic acid | 130.09 | | 0.68 | | 1.36 | | 1.03 | | 0.005 | | | pos |
|  | |  | | Phenylalanylproline | 245.13 | | 3.54 | | 1.21 | | 1.03 | | 0.031 | | | pos |
|  | | Hybrid peptides | | Beta-Alanyl-L-lysine | 476.32 | | 8.31 | | 1.87 | | 1.07 | | 0.003 | | | pos |
|  | |  | | Astin I | 516.20 | | 2.93 | | 1.45 | | 1.05 | | 0.038 | | | pos |
|  | | Carboxylic acid derivatives | | Dihydroceramide | 374.29 | | 8.95 | | 2.76 | | 0.78 | | 0.017 | | | neg |
|  | | Organic carbonic acids | | Moracin L | 342.13 | | 5.06 | | 2.02 | | 1.17 | | 0.037 | | | pos |
|  | | Fumonisins | | Fumonisin B1 | 722.40 | | 5.05 | | 1.28 | | 1.02 | | 0.005 | | | pos |
| Organic oxygen compounds | | Carbohydrates and carbohydrate conjugates | | Dihydromaleimide beta-D-glucoside | 260.08 | | 1.40 | | 1.43 | | 1.06 | | 0.049 | | | neg |
|  | |  | | Pisatoside | 260.08 | | 1.63 | | 1.85 | | 1.08 | | 0.008 | | | neg |
|  | |  | | Tyramine glucuronide | 294.10 | | 2.97 | | 1.82 | | 1.05 | | 0.006 | | | neg |
|  | |  | | N-Acetylneuraminic acid | 274.09 | | 2.80 | | 1.72 | | 1.08 | | 0.047 | | | pos |
|  | |  | | Lotaustralin | 226.11 | | 2.79 | | 1.20 | | 1.02 | | 0.014 | | | pos |
|  | |  | | Indican | 296.11 | | 2.97 | | 1.92 | | 1.05 | | 0.003 | | | pos |
|  | | Ethers | | Digoxigenin monodigitoxoside | 565.28 | | 8.94 | | 1.84 | | 1.05 | | 0.001 | | | pos |
|  | |  | | Heptaethylene glycol | 327.20 | | 7.69 | | 1.09 | | 1.02 | | 0.024 | | | pos |
|  | | Carbonyl compounds | | 1-Hydroxyepiacorone | 289.12 | | 3.60 | | 1.90 | | 1.10 | | 0.008 | | | neg |
| Organoheterocyclic compounds | | 1,3-dioxolanes | | Valeraldehyde propyleneglycol acetal | 125.10 | | 4.29 | | 1.23 | | 1.03 | | 0.050 | | | neg |
|  | | Gamma butyrolactones | | 3-Hydroxyadipic acid 3,6-lactone | 143.04 | | 1.62 | | 1.62 | | 0.89 | | 0.018 | | | neg |
|  | |  | |  |  | |  | |  | |  | |  | | |  |
| **Table S7 (continued)** | | | | | | | | | | | | | | | | |
| **HMDB Superclass** | **HMDB Subclass** | | **Metabolite** | | | **M/Z** | | **Retention time** | | **VIP** | | **FC(C/S)** | | ***P*-value** | **Mode** | |
|  | | Hydroxyindoles | | 11-beta-Hydroxyandrosterone-3-glucuronide | 463.24 | | 8.09 | | 3.93 | | 0.49 | | 0.003 | | | neg |
|  | | Pterins and derivatives | | 2-Amino-4-oxo-6-(1',2'-dioxoprolyl)-7,8-dihydroxypteridine | 288.03 | | 1.24 | | 1.23 | | 0.96 | | 0.018 | | | neg |
|  | | Tryptamines and derivatives | | N-Methyl-1H-indole-3-propanamide | 203.12 | | 4.31 | | 1.25 | | 1.03 | | 0.016 | | | pos |
|  | |  | | Jasmine ketolactone | 253.11 | | 5.43 | | 1.79 | | 1.17 | | 0.027 | | | neg |
|  | |  | | Isoquinoline | 130.06 | | 4.87 | | 1.94 | | 1.07 | | 0.008 | | | pos |
|  | |  | | 3alpha-Hydroxyoreadone | 285.17 | | 3.39 | | 1.63 | | 1.04 | | 0.002 | | | pos |
| Phenylpropanoids and polyketides | | Flavonoid glycosides | | 6''-Malonylcosmosiin | 517.10 | | 4.90 | | 1.21 | | 0.97 | | 0.007 | | | neg |
|  | |  | | 1-Hydroxyibuprofen | 221.12 | | 5.74 | | 1.79 | | 0.89 | | 0.026 | | | neg |
|  | |  | | 3-(3,4-Dihydroxyphenyl)-2-methylpropionic acid | 195.07 | | 5.42 | | 1.63 | | 0.95 | | 0.016 | | | neg |
|  | |  | | (Z)-7-Hexadecen-1,16-olide | 294.24 | | 11.13 | | 1.61 | | 0.94 | | 0.046 | | | pos |
|  | |  | | Neosaxitoxin | 280.12 | | 4.14 | | 2.76 | | 1.19 | | 0.003 | | | pos |
| Benzenoids | | Benzenediols | | 3-(3,4-dihydroxyphenyl)-N-[2-(4-hydroxyphenyl) ethyl] propanimidic acid | 338.08 | | 1.28 | | 1.31 | | 0.95 | | 0.014 | | | neg |
|  | |  | | 3-Phenylpropyl acetate | 179.11 | | 3.66 | | 1.15 | | 0.97 | | 0.024 | | | pos |
|  | |  | | 1,1-Dimethoxy-2-phenylethane | 167.11 | | 3.59 | | 1.19 | | 1.04 | | 0.034 | | | pos |
| Alkaloids and derivatives | |  | | Dextrorphan O-glucuronide | 398.20 | | 8.45 | | 1.37 | | 1.03 | | 0.003 | | | pos |
| Others | |  | | C-2 Ceramide | 342.30 | | 9.05 | | 1.41 | | 1.04 | | 0.008 | | | pos |
|  | |  | | 3-Methyl-1-phenyl-1-butanone | 163.11 | | 5.69 | | 1.41 | | 0.94 | | 0.030 | | | pos |
|  | |  | | Amprotropine | 308.22 | | 4.74 | | 1.84 | | 1.06 | | 0.037 | | | pos |
|  | |  | | L-Homotyrosine | 196.10 | | 1.69 | | 1.70 | | 1.05 | | 0.000 | | | pos |
|  | |  | | 3-Indolepropionic acid | 190.09 | | 4.87 | | 1.65 | | 1.04 | | 0.017 | | | pos |
|  | |  | | Indole-3-carboxaldehyde | 354.15 | | 3.15 | | 1.57 | | 1.04 | | 0.002 | | | pos |
|  | |  | | Pyridoxine (Vitamin B6) | 170.08 | | 0.73 | | 1.46 | | 1.03 | | 0.004 | | | pos |
|  | |  | | Trans-EKODE-(E)-Ib | 311.22 | | 7.41 | | 1.39 | | 1.03 | | 0.012 | | | pos |
|  | |  | | PHENACYLAMINE | 136.07 | | 4.08 | | 1.39 | | 0.96 | | 0.048 | | | pos |
|  | |  | | PE (18:1(9Z)/0:0) | 480.31 | | 8.69 | | 1.36 | | 1.03 | | 0.031 | | | pos |
|  | |  | | Polyoxyethylene (600) mono- ricinoleate | 341.30 | | 8.44 | | 1.27 | | 1.02 | | 0.024 | | | pos |
|  | |  | | 9-hydroxy-12Z-octadecenoic acid | 299.26 | | 8.47 | | 1.10 | | 1.01 | | 0.016 | | | pos |
|  | |  | |  |  | |  | |  | |  | |  | | |  |
| **Table S7 (continued)** | | | | | | | | | | | | | | | | |
| **HMDB Superclass** | **HMDB Subclass** | | **Metabolite** | | | **M/Z** | | **Retention time** | | **VIP** | | **FC(C/S)** | | ***P*-value** | **Mode** | |
|  | |  | | 9,10-Epoxy-18-hydroxystearate | 315.25 | | 8.36 | | 1.03 | | 1.03 | | 0.033 | | | pos |
|  | |  | | 12-OPDA | 293.21 | | 7.02 | | 1.02 | | 1.02 | | 0.020 | | | pos |
| Diets: S, steam-flaked corn and corn silage diet; C, corn and corn silage diet; HMDB, human metabolome database; M/Z, mass-to-charge ratio; FC, fold change; VIP, variable importance in the projection; pos, in positive ion mode; neg, in negative ion mode. | | | | | | | | | | | | | | | | |

| **Table S5 Significantly differential metabolites between the glucogenic diet C and lipogenic diet L after 48 h *in vitro* fermentation with rumen fluid of dairy cows** | | | | | | | | |
| --- | --- | --- | --- | --- | --- | --- | --- | --- |
| **HMDB Superclass** | **HMDB Subclass** | **Metabolite** | **M/Z** | **Retention time** | **VIP** | **FC(L/C)** | ***P*-value** | **Mode** |
| Lipids and lipid-like molecules | Fatty acids and conjugates | Myristoleic acid | 191.18 | 9.14 | 2.77 | 1.65 | < 0.001 | pos |
|  |  | Goshuyic acid | 247.17 | 9.13 | 2.16 | 1.57 | < 0.001 | pos |
|  |  | 2-Octenoic acid | 302.23 | 6.36 | 1.71 | 1.33 | < 0.001 | pos |
|  |  | Stearic acid | 307.26 | 7.14 | 1.46 | 1.14 | < 0.001 | pos |
|  |  | 5-Hexyl-2-furanhexanoic acid | 289.18 | 5.85 | 1.19 | 1.09 | < 0.001 | pos |
|  |  | 13-hydroxyoctadecanoic acid | 283.26 | 9.43 | 1.63 | 0.69 | 0.005 | pos |
|  |  | 10-Hydroxy-2,8-decadiene-4,6-diynoic acid | 194.08 | 3.22 | 1.31 | 0.85 | < 0.001 | pos |
|  |  | Pentadecanoic acid | 275.26 | 8.76 | 1.18 | 0.89 | < 0.001 | pos |
|  |  | Petroselinic acid | 283.26 | 10.95 | 1.18 | 0.93 | < 0.001 | pos |
|  |  | Dioscoretine | 242.17 | 4.93 | 1.17 | 1.12 | 0.003 | pos |
|  |  | Floionolic acid | 315.25 | 9.73 | 1.10 | 0.94 | < 0.001 | pos |
|  |  | 2-Octenedioic acid | 217.07 | 2.24 | 2.12 | 2.78 | < 0.001 | neg |
|  |  | 2-hydroxyhexadecanoic acid | 271.23 | 9.10 | 1.34 | 1.10 | < 0.001 | neg |
|  |  | 5-Tetradecenoic acid | 271.19 | 7.16 | 1.27 | 1.33 | < 0.001 | neg |
|  |  | 2-Hydroxymyristic Acid | 243.20 | 8.45 | 1.14 | 1.10 | < 0.001 | neg |
|  |  | (S)-10,16-Dihydroxyhexadecanoic acid | 287.22 | 7.97 | 1.07 | 1.13 | 0.001 | neg |
|  |  | 3,3-Dimethylglutaric acid | 159.07 | 3.81 | 1.38 | 0.81 | < 0.001 | neg |
|  |  | (9S,10S)-9,10-dihydroxyoctadecanoate | 315.25 | 8.59 | 1.24 | 1.27 | 0.019 | neg |
|  |  | 3b,15b,17a-Trihydroxy-pregnenone | 329.21 | 8.09 | 1.23 | 0.54 | 0.039 | neg |
|  |  | (R)-3-Hydroxy-Octadecanoic acid | 345.26 | 9.04 | 1.09 | 0.61 | 0.034 | neg |
|  | Fatty acid esters | Butyl 2-decenoate | 191.18 | 9.22 | 2.85 | 1.85 | < 0.001 | pos |
|  |  | 4,8 dimethylnonanoyl carnitine | 330.26 | 8.46 | 1.05 | 0.92 | < 0.001 | pos |
|  |  | Dipropyl hexanedioate | 213.15 | 4.52 | 1.03 | 0.95 | < 0.001 | pos |
|  |  | Stearoyllactic acid | 355.28 | 9.88 | 2.31 | 2.57 | < 0.001 | neg |
|  |  | Ethyl (Z, Z)-5,8-tetradecadienoate | 297.21 | 6.53 | 1.22 | 1.17 | < 0.001 | neg |
|  |  | Oleoylcarnitine | 462.30 | 8.73 | 1.06 | 0.86 | < 0.001 | neg |
|  | Fatty acyl glycosides | 1-Octen-3-yl primeveroside | 445.20 | 7.93 | 2.23 | 0.71 | < 0.001 | pos |
|  |  | 4-Hydroxyproline galactoside | 258.10 | 3.23 | 1.86 | 0.79 | < 0.001 | pos |
|  |  | 6S,9R-Dihydroxy-4,7E-megastigmadien-3-one 9-[apiosyl-(1->6)-glucoside] | 563.23 | 8.41 | 1.26 | 1.14 | < 0.001 | neg |
|  | Fatty alcohols | 1-Undecanol | 367.36 | 10.50 | 1.64 | 1.90 | < 0.001 | pos |
|  |  | Avocadene | 304.29 | 6.62 | 1.42 | 1.12 | < 0.001 | pos |
|  |  | Avocadyne | 329.23 | 8.91 | 1.24 | 1.32 | < 0.001 | neg |
|  | Triterpenoids | Tomentosolic acid | 437.34 | 9.48 | 3.02 | 1.55 | < 0.001 | pos |
|  |  |  |  |  |  |  |  |  |
| **Table S5** **(continued)** | | | | | | | | |
| **HMDB Superclass** | **HMDB Subclass** | **Metabolite** | **M/Z** | **Retention time** | **VIP** | **FC(L/C)** | ***P*-value** | **Mode** |
|  |  | Ursolic acid | 457.37 | 8.90 | 3.00 | 1.56 | < 0.001 | pos |
|  |  | Camelledionol | 423.33 | 8.71 | 2.84 | 1.52 | < 0.001 | pos |
|  |  | Oleanolic acid | 457.37 | 9.05 | 2.59 | 1.61 | < 0.001 | pos |
|  |  | Sandosapogenol | 439.36 | 8.90 | 2.55 | 1.31 | < 0.001 | pos |
|  |  | (3beta,17alpha,23S,24S)-17,23-Epoxy-3,24,29-trihydroxy-27-norlanost-8-en-15-one | 457.33 | 7.07 | 2.37 | 1.48 | < 0.001 | pos |
|  |  | Asperagenin | 449.33 | 7.19 | 1.79 | 1.18 | < 0.001 | pos |
|  |  | Gamma-Taraxastane-3,20-diol | 427.39 | 10.37 | 1.34 | 1.10 | 0.001 | pos |
|  |  | Lucidenic acid E2 | 539.26 | 9.24 | 1.33 | 1.07 | < 0.001 | pos |
|  |  | Ganoderenic acid E | 551.27 | 8.85 | 1.01 | 1.04 | < 0.001 | pos |
|  |  | 3beta-Acetoxy-19alpha-hydroxy-12-ursene | 502.42 | 10.48 | 1.71 | 0.57 | 0.019 | pos |
|  |  | Glyuranolide | 530.34 | 10.24 | 1.29 | 1.12 | 0.012 | pos |
|  |  | Melilotoside D | 527.29 | 7.52 | 1.21 | 0.91 | < 0.001 | pos |
|  |  | Medicoside C | 461.25 | 7.59 | 1.04 | 0.94 | < 0.001 | pos |
|  |  | Glabric acid | 531.33 | 6.84 | 2.31 | 1.65 | < 0.001 | neg |
|  |  | Ganoderic acid A | 497.29 | 8.39 | 2.27 | 1.98 | < 0.001 | neg |
|  |  | Ganoderiol I | 501.36 | 9.14 | 2.22 | 1.34 | < 0.001 | neg |
|  |  | Medicagenic acid | 547.33 | 7.39 | 1.59 | 1.35 | < 0.001 | neg |
|  |  | (3beta,19alpha)-3,19,23,24-Tetrahydroxy-12-oleanen-28-oic acid | 539.33 | 6.96 | 1.54 | 1.19 | < 0.001 | neg |
|  | Sesquiterpenoids | Ipomeatetrahydrofuran | 239.20 | 7.87 | 2.27 | 1.42 | < 0.001 | pos |
|  |  | Auberganol | 241.22 | 8.03 | 1.73 | 1.28 | < 0.001 | pos |
|  |  | 4,7-Megastigmadien-9-ol | 411.33 | 8.63 | 1.22 | 1.08 | < 0.001 | pos |
|  |  | Sterebin A | 343.25 | 5.87 | 1.06 | 1.05 | < 0.001 | pos |
|  |  | 7(14)-Bisabolene-2,3,10,11-tetrol | 255.19 | 6.43 | 2.09 | 0.64 | < 0.001 | pos |
|  |  | Curcumol | 269.21 | 7.04 | 1.26 | 0.85 | < 0.001 | pos |
|  |  | 7-Hydroxytrichodermol | 267.16 | 4.49 | 1.03 | 0.94 | < 0.001 | pos |
|  |  | Deoxynivalenol 3-glucoside | 491.21 | 9.33 | 1.02 | 0.90 | < 0.001 | pos |
|  |  | 7(14)-Farnesene-9,12-diol | 287.22 | 7.80 | 1.26 | 1.35 | < 0.001 | neg |
|  | Terpene lactones | 8-Deoxy-11,13-dihydroxygrosheimin | 298.18 | 4.86 | 1.76 | 0.76 | < 0.001 | pos |
|  |  | Isoalantolactone | 233.15 | 6.28 | 1.43 | 0.90 | < 0.001 | pos |
|  |  | Tatridin B | 229.12 | 6.15 | 1.30 | 0.90 | < 0.001 | pos |
|  |  | Dihydrocumambrin A | 307.16 | 7.83 | 2.29 | 3.19 | < 0.001 | neg |
|  |  | 2alpha-Hydroxyalantolactone | 229.12 | 7.92 | 1.73 | 0.67 | < 0.001 | neg |
|  |  | 4,11,13,15-Tetrahydroridentin B | 249.15 | 7.03 | 1.39 | 0.76 | < 0.001 | neg |
|  |  |  |  |  |  |  |  |  |
| **Table S5** **(continued)** | | | | | | | | |
| **HMDB Superclass** | **HMDB Subclass** | **Metabolite** | **M/Z** | **Retention time** | **VIP** | **FC(L/C)** | ***P*-value** | **Mode** |
|  |  | Tavulin | 245.12 | 6.17 | 1.28 | 0.85 | < 0.001 | neg |
|  |  | Crispolide | 261.11 | 4.48 | 1.18 | 0.83 | < 0.001 | neg |
|  | Monoterpenoids | Withangulatin A | 549.25 | 8.69 | 2.48 | 1.32 | < 0.001 | pos |
|  |  | Linalyl propionate | 243.20 | 5.46 | 1.13 | 1.17 | < 0.001 | pos |
|  |  | Piperitone | 170.15 | 5.71 | 1.44 | 1.34 | < 0.001 | pos |
|  |  | Alpha-Terpineol propanoate | 211.17 | 7.24 | 1.01 | 0.86 | < 0.001 | pos |
|  |  | 3-(5,6,6-Trimethylbicyclo [2.2.1] hept-1-yl) cyclohexanol | 281.21 | 7.58 | 1.64 | 1.46 | < 0.001 | neg |
|  |  | Soyasapogenol B 3-O-b-D-glucuronide | 671.35 | 7.95 | 1.28 | 1.24 | < 0.001 | neg |
|  |  | Valechlorin | 457.17 | 8.02 | 1.38 | 1.13 | < 0.001 | neg |
|  | Steroid lactones | Withaperuvin H | 611.29 | 8.17 | 2.73 | 1.55 | < 0.001 | pos |
|  |  | Physagulin F | 567.26 | 8.21 | 1.80 | 1.19 | < 0.001 | pos |
|  |  | Physagulin C | 565.24 | 8.41 | 1.25 | 1.07 | < 0.001 | pos |
|  |  | Physapubenolide | 565.25 | 8.23 | 1.43 | 1.42 | < 0.001 | neg |
|  |  | (5alpha,6beta,14alpha,20R,22R)-5,6,14,20,27-Pentahydroxy-1-oxowith-24-enolide | 505.28 | 5.66 | 1.35 | 1.20 | < 0.001 | neg |
|  | Diterpenoids | Phytocassane B | 317.21 | 6.59 | 1.29 | 1.14 | < 0.001 | pos |
|  |  | Austroinulin | 355.28 | 6.68 | 1.13 | 1.06 | < 0.001 | pos |
|  |  | Beta-Tocopheryl quinone | 433.37 | 10.23 | 1.96 | 1.90 | < 0.001 | pos |
|  |  | (13R,14R)-7-Labdene-13,14,15-triol | 357.30 | 8.36 | 1.01 | 0.95 | < 0.001 | pos |
|  | Lineolic acids and derivatives | Punicic acid | 243.21 | 7.35 | 1.51 | 0.85 | < 0.001 | pos |
|  |  | 13S-hydroxyoctadecadienoic acid | 329.27 | 9.87 | 1.31 | 0.87 | < 0.001 | pos |
|  |  | 2-Hydroxylinolenic acid | 317.21 | 7.35 | 1.22 | 0.88 | < 0.001 | pos |
|  |  | Cibaric acid | 369.19 | 6.18 | 1.27 | 1.22 | < 0.001 | neg |
|  | Diradylglycerols | DG(16:0/16:0/0:0) | 591.50 | 10.92 | 1.34 | 1.23 | < 0.001 | pos |
|  |  | DG(15:0/18:0/0:0) | 627.52 | 11.53 | 1.31 | 0.77 | < 0.001 | neg |
|  |  | DG(20:1(11Z)/22:5(4Z,7Z,10Z,13Z,16Z)/0:0) | 677.55 | 9.98 | 1.17 | 0.82 | < 0.001 | neg |
|  | Terpene glycosides | Melilotin | 549.30 | 7.50 | 1.33 | 0.87 | < 0.001 | pos |
|  |  | Calenduloside E | 677.39 | 7.77 | 2.64 | 2.72 | < 0.001 | neg |
|  |  | L-Citronellol glucoside | 317.20 | 5.63 | 1.00 | 1.15 | < 0.001 | neg |
|  | Glycerophosphocholines | PC(16:0/18:2(9Z,12Z)) | 802.56 | 11.16 | 1.24 | 0.84 | < 0.001 | neg |
|  |  | LysoPC(16:0) | 540.33 | 8.36 | 1.16 | 0.86 | < 0.001 | neg |
|  | Glycerophosphoethanolamines | PE(14:1(9Z)/16:1(9Z)) | 660.46 | 10.33 | 2.29 | 0.64 | < 0.001 | pos |
|  |  | LysoPE(0:0/16:0) | 436.28 | 8.40 | 1.54 | 0.80 | < 0.001 | pos |
|  | Hydroxysteroids | Tetrahydrodeoxycorticosterone | 379.22 | 8.98 | 1.58 | 1.19 | < 0.001 | pos |
|  |  |  |  |  |  |  |  |  |
| **Table S5** **(continued)** | | | | | | | | |
| **HMDB Superclass** | **HMDB Subclass** | **Metabolite** | **M/Z** | **Retention time** | **VIP** | **FC(L/C)** | ***P*-value** | **Mode** |
|  |  | Prednisone | 379.15 | 8.92 | 1.13 | 0.85 | < 0.001 | neg |
|  | Pregnane steroids | 17a-Hydroxypregnenolone | 333.24 | 5.75 | 2.83 | 1.77 | < 0.001 | pos |
|  |  | Halobetasol Propionate | 521.13 | 7.58 | 1.97 | 1.86 | < 0.001 | neg |
|  | Steroidal glycosides | Corchoroside B | 541.28 | 9.01 | 1.48 | 1.12 | < 0.001 | pos |
|  |  | Torvoside D | 747.39 | 7.73 | 2.25 | 1.86 | < 0.001 | neg |
|  | Bile acids, alcohols and derivatives | 5beta-Cholestane-3alpha,7alpha,24,26-tetrol | 401.34 | 7.36 | 1.16 | 0.92 | < 0.001 | pos |
|  | Glycerophosphates | 1-(9Z-tetradecenoyl)-glycero-3-phosphate | 379.19 | 8.11 | 1.31 | 0.66 | < 0.001 | neg |
|  | Cholestane steroids | 5a-Cholest-8-en-3b-ol | 409.35 | 10.61 | 1.01 | 0.89 | < 0.001 | pos |
|  | Fatty amides | Palmitoyl Serinol | 330.30 | 8.94 | 1.89 | 1.36 | < 0.001 | pos |
|  | Monoradylglycerols | MG(i-12:0/0:0/0:0) | 255.20 | 8.07 | 1.40 | 1.18 | < 0.001 | neg |
|  | Quinone and hydroquinone lipids | 13'-Carboxy-alpha-tocopherol | 493.39 | 11.52 | 1.74 | 0.69 | < 0.001 | pos |
|  | Steroid esters | Physapubescin | 553.28 | 7.95 | 1.76 | 1.17 | < 0.001 | pos |
|  | Stigmastanes and derivatives | 6-Deoxohomodolichosterone | 463.38 | 10.59 | 1.11 | 1.12 | < 0.001 | pos |
|  | Eicosanoids | Carboprost Tromethamine | 367.25 | 7.62 | 1.27 | 0.81 | < 0.001 | neg |
|  | Ergostane steroids | Delta 8,14 -Sterol | 393.35 | 12.76 | 1.08 | 0.91 | < 0.001 | pos |
| Organic acids and derivatives | Amino acids, peptides, and analogues | N6-Acetyl-5S-hydroxy-L-lysine | 243.08 | 4.68 | 2.31 | 0.42 | < 0.001 | pos |
|  |  | N-[[3-Hydroxy-2-(2-pentenyl) cyclopentyl]acetyl]isoleucine | 308.22 | 7.67 | 1.93 | 1.27 | < 0.001 | pos |
|  |  | Prolyl-Tyrosine | 323.10 | 5.66 | 1.48 | 1.27 | 0.007 | pos |
|  |  | 5-Hydroxyindoleacetylglycine | 213.07 | 3.25 | 1.04 | 1.13 | 0.004 | pos |
|  |  | Cytidine 2',3'-cyclic phosphate | 350.01 | 5.50 | 2.13 | 0.68 | 0.001 | pos |
|  |  | Valyl-Glutamate | 229.12 | 1.00 | 1.75 | 0.83 | < 0.001 | pos |
|  |  | Isoleucyl-Glutamate | 243.13 | 1.41 | 1.72 | 0.85 | < 0.001 | pos |
|  |  | Tetrahydrodipicolinate | 204.09 | 1.57 | 1.66 | 0.80 | < 0.001 | pos |
|  |  | Phenylalanylproline | 245.13 | 3.54 | 1.29 | 0.91 | < 0.001 | pos |
|  |  | Glutamylleucine | 243.13 | 0.76 | 1.02 | 0.95 | < 0.001 | pos |
|  |  | 4-Hydroxystachydrine | 158.08 | 3.22 | 1.60 | 1.61 | 0.001 | neg |
|  |  | N-Acetylproline | 138.06 | 2.85 | 1.34 | 1.46 | 0.001 | neg |
|  |  | N-Arachidonoyl glycine | 406.26 | 8.05 | 1.24 | 0.71 | 0.002 | neg |
|  |  | 2-Hepteneoylglycine | 230.10 | 3.07 | 2.25 | 0.35 | < 0.001 | neg |
|  |  | Captopril-cysteine disulfide | 371.05 | 8.00 | 1.96 | 0.37 | < 0.001 | neg |
|  |  |  |  |  |  |  |  |  |
|  |  |  |  |  |  |  |  |  |
| **Table S5** **(continued)** | | | | | | | | |
| **HMDB Superclass** | **HMDB Subclass** | **Metabolite** | **M/Z** | **Retention time** | **VIP** | **FC(L/C)** | ***P*-value** | **Mode** |
|  |  | (1R)-Glutathionyl-(2R)-hydroxy-1,2-dihydronaphthalene | 472.12 | 5.47 | 1.78 | 0.52 | < 0.001 | neg |
|  |  | N-(4-Hydroxycinnamoyl) tyrosine | 362.08 | 5.41 | 1.54 | 0.75 | < 0.001 | neg |
|  |  | Indolylacryloylglycine | 225.07 | 3.35 | 1.41 | 0.89 | < 0.001 | neg |
|  |  | Gamma-Glutamyl-S-methylcysteine sulfoxide | 279.07 | 4.76 | 1.20 | 0.82 | 0.043 | neg |
|  |  | 3-Hydroxymugineic acid | 357.09 | 5.55 | 1.16 | 0.81 | 0.027 | neg |
|  | Hybrid peptides | Beta-Alanyl-L-lysine | 476.32 | 8.31 | 1.77 | 0.80 | < 0.001 | pos |
|  | Carboxylic acid derivatives | Dihydroceramide | 374.29 | 8.95 | 1.53 | 1.40 | < 0.001 | neg |
|  | Medium-chain hydroxy acids and derivatives | 12-Hydroxydodecanoic acid | 258.21 | 5.72 | 1.47 | 0.85 | < 0.001 | pos |
|  | Organic carbonic acids | Moracin L | 342.13 | 5.06 | 1.39 | 0.78 | < 0.001 | pos |
| Organic oxygen compounds | Carbohydrates and carbohydrate conjugates | 3,4,5-trihydroxy-6-[(4-methoxy-1-benzofuran-6-yl) oxy]oxane-2-carboxylic acid | 363.07 | 6.81 | 1.89 | 1.35 | < 0.001 | pos |
|  |  | 4-Methoxybenzyl O-(2-sulfoglucoside) | 345.06 | 6.38 | 1.87 | 1.71 | 0.002 | pos |
|  |  | Arbutin | 317.06 | 9.96 | 1.48 | 1.19 | 0.001 | pos |
|  |  | Indican | 296.11 | 2.97 | 2.84 | 0.66 | < 0.001 | pos |
|  |  | N-Acetylneuraminic acid | 274.09 | 2.80 | 2.10 | 0.63 | < 0.001 | pos |
|  |  | 6-(5-ethyl-2,3-dihydroxyphenoxy)-3,4,5-trihydroxyoxane-2-carboxylic acid | 295.08 | 3.42 | 1.85 | 1.47 | 0.001 | pos |
|  |  | (2S)-2-Butanol O-[b-D-Apiofuranosyl-(1->6)-b-D-glucopyranoside] | 410.20 | 8.36 | 1.22 | 0.90 | < 0.001 | pos |
|  |  | Lotaustralin | 226.11 | 2.79 | 1.17 | 0.94 | < 0.001 | pos |
|  |  | Dihydromaleimide beta-D-glucoside | 260.08 | 1.40 | 2.79 | 0.24 | < 0.001 | neg |
|  |  | Tyramine glucuronide | 294.10 | 2.97 | 2.35 | 0.68 | < 0.001 | neg |
|  |  | Pisatoside | 260.08 | 1.63 | 2.26 | 0.57 | < 0.001 | neg |
|  |  | 3-oxo-3-[(3,4,5,6-tetrahydroxyoxan-2-yl) methoxy] propanoic acid | 247.05 | 2.90 | 1.57 | 0.74 | < 0.001 | neg |
|  |  | Fluoxetine glucuronide | 520.14 | 7.36 | 1.55 | 0.52 | 0.003 | neg |
|  |  | 6-Sinapoylglucoraphenin | 640.08 | 0.83 | 1.08 | 0.91 | < 0.001 | neg |
|  | Carbonyl compounds | 3-Propyl-1,2-cyclopentanedione | 182.12 | 4.51 | 1.99 | 0.48 | 0.006 | pos |
|  |  | 4-Heptanone | 115.11 | 3.45 | 1.01 | 1.11 | 0.003 | pos |
|  |  | 3-Methylcyclopentadecanone | 283.23 | 8.91 | 1.14 | 1.20 | 0.001 | neg |
|  |  | 2-Nonanone | 187.13 | 4.81 | 1.07 | 1.17 | < 0.001 | neg |
|  |  | 1-Hydroxyepiacorone | 289.12 | 3.60 | 1.33 | 0.77 | < 0.001 | neg |
|  |  |  |  |  |  |  |  |  |
|  |  |  |  |  |  |  |  |  |
| **Table S5** **(continued)** | | | | | | | | |
| **HMDB Superclass** | **HMDB Subclass** | **Metabolite** | **M/Z** | **Retention time** | **VIP** | **FC(L/C)** | ***P*-value** | **Mode** |
| Organoheterocyclic compounds | 1-benzopyrans | Theaflagallin | 365.06 | 7.11 | 2.15 | 1.45 | 0.010 | pos |
|  |  |  |  |  |  |  |  |  |
|  |  | 9'-Carboxy-gamma-chromanol | 421.26 | 7.53 | 1.19 | 1.20 | < 0.001 | neg |
|  |  | Gamma-CEHC | 263.13 | 5.26 | 1.30 | 0.75 | 0.001 | neg |
|  | Hydropyridines | (E)-5-(3,4,5,6-Tetrahydro-3-pyridylidenemethyl)-2-furanmethanol | 209.13 | 2.85 | 1.09 | 0.88 | 0.015 | pos |
|  |  | 2-Hydroxypyridine | 140.04 | 2.51 | 1.46 | 1.58 | < 0.001 | neg |
|  |  | 5-(2-Furanyl)-1,2,3,4,5,6-hexahydro-7H-cyclopenta[b]pyridin-7-one | 248.09 | 3.96 | 1.24 | 0.72 | 0.001 | neg |
|  | Gamma butyrolactones | 5-Nonyltetrahydro-2-oxo-3-furancarboxylic acid | 237.15 | 6.26 | 1.07 | 1.16 | 0.002 | neg |
|  |  | Artabsinolide A | 261.11 | 6.13 | 1.16 | 0.73 | 0.012 | neg |
|  |  | 3-Hydroxyadipic acid 3,6-lactone | 143.04 | 1.62 | 1.15 | 0.68 | 0.009 | neg |
|  | Indolyl carboxylic acids and derivatives | L-Tryptophan | 426.22 | 2.88 | 1.43 | 1.19 | 0.017 | pos |
|  |  | Indoleacetic acid | 220.06 | 3.70 | 1.16 | 0.56 | 0.029 | neg |
|  |  | 5-Hydroxyindoleacetic acid | 190.05 | 3.53 | 1.39 | 0.70 | 0.005 | neg |
|  | Dibenzoxepines | Doxepin N-oxide glucuronide | 507.17 | 7.28 | 2.11 | 2.19 | 0.011 | neg |
|  | Benzoxazinones | (R)-2,7-Dihydroxy-2H-1,4-benzoxazin-3(4H)-one | 380.11 | 5.58 | 1.62 | 0.81 | < 0.001 | pos |
|  | Benzylisoquinolines | 8-Propanoylneosolaniol | 473.16 | 7.24 | 1.92 | 0.64 | 0.001 | neg |
|  | Bilirubins | D-Urobilin | 609.27 | 8.19 | 2.16 | 1.79 | < 0.001 | neg |
|  | Hydroxyindoles | 11-beta-Hydroxyandrosterone-3-glucuronide | 463.24 | 8.09 | 1.57 | 2.22 | 0.041 | neg |
|  | Indoles | Indoleacrylic acid | 188.07 | 3.41 | 1.62 | 0.81 | < 0.001 | pos |
|  | Methylpyridines | 2-Ethyl-5-methylpyridine | 139.12 | 1.87 | 1.51 | 1.41 | 0.020 | pos |
|  | Pyranones and derivatives | Erinapyrone A | 187.06 | 2.69 | 1.17 | 1.21 | < 0.001 | neg |
|  | Quinolones and derivatives | Quinoline-4,8-diol | 206.05 | 2.91 | 1.15 | 0.85 | 0.007 | neg |
|  | Unclassified | Canescein | 611.25 | 8.45 | 2.77 | 1.84 | < 0.001 | pos |
|  | Unclassified | (+)-2,3-Dihydro-3-methyl-1H-pyrrole | 125.11 | 1.14 | 1.43 | 1.16 | 0.018 | pos |
|  | Unclassified | (S)-N-Methylsalsolinol | 158.10 | 3.24 | 1.42 | 0.80 | 0.001 | pos |
|  | Unclassified | Isoquinoline | 130.06 | 4.87 | 1.21 | 0.90 | < 0.001 | pos |
|  | Unclassified | 3-Pyridinebutanoic acid | 210.08 | 4.75 | 1.17 | 1.26 | < 0.001 | neg |
|  | Unclassified | N-Carbamoyl glucuronide lorcaserin | 454.17 | 8.36 | 1.42 | 0.74 | < 0.001 | neg |
|  |  |  |  |  |  |  |  |  |
|  |  |  |  |  |  |  |  |  |
| **Table S5** **(continued)** | | | | | | | | |
| **HMDB Superclass** | **HMDB Subclass** | **Metabolite** | **M/Z** | **Retention time** | **VIP** | **FC(L/C)** | ***P*-value** | **Mode** |
| Phenylpropanoids and polyketides | Furanocoumarins | Edulisin I | 525.15 | 8.03 | 1.27 | 1.21 | 0.006 | neg |
|  |  | (R)-Heraclenol 2'-(3-methyl-2-butenoate) | 385.13 | 7.70 | 1.11 | 0.75 | 0.008 | neg |
|  | Hydroxycinnamic acids and derivatives | Sinapinic acid-O-glucuronide isomer | 365.09 | 4.79 | 1.23 | 1.22 | 0.034 | pos |
|  |  | N-(p-Hydroxyphenyl) ethyl p-hydroxycinnamide | 318.09 | 4.63 | 1.45 | 1.91 | 0.025 | neg |
|  | Anthocyanidins | 2-{2-[(6-carboxy-3,4,5-trihydroxyoxan-2-yl) oxy] phenyl}-1lambda⁴-chromen-1-ylium | 434.08 | 5.84 | 1.90 | 1.79 | 0.025 | neg |
|  | Coumarin glycosides | Aesculin | 363.07 | 7.11 | 1.94 | 1.32 | 0.012 | pos |
|  | Cyclic diarylheptanoids | Myricatomentoside II | 571.16 | 8.02 | 1.37 | 1.31 | 0.010 | neg |
|  | Flavans | 2-phenyl-3,4-dihydro-2H-1-benzopyran-3,5,7-triol | 259.10 | 3.88 | 1.03 | 0.83 | 0.044 | pos |
|  | Flavonoid glycosides | Delphinidin 3-rutinoside | 594.16 | 7.01 | 1.06 | 0.85 | 0.008 | pos |
|  | Furanoisoflavonoids | Kanzonol F | 443.18 | 7.09 | 1.52 | 1.43 | 0.040 | pos |
|  | Pyranocoumarins | Trans-O-Methylgrandmarin | 327.09 | 3.20 | 1.06 | 0.91 | 0.001 | neg |
|  | Pyranoflavonoids | Cycloartocarpin | 479.17 | 7.66 | 1.56 | 1.41 | 0.002 | neg |
|  | Stilbene glycosides | 3,4,5-trihydroxy-6-(2-hydroxy-1,2-diphenylethoxy) oxane-2-carboxylic acid | 408.17 | 4.48 | 1.29 | 0.89 | < 0.001 | pos |
|  | Unclassified | 3-(1,2-dihydroxybutyl)-7-hydroxy-1H-isochromen-1-one | 268.12 | 4.98 | 1.84 | 0.58 | 0.001 | pos |
|  |  | Neosaxitoxin | 280.12 | 4.14 | 1.60 | 0.79 | < 0.001 | pos |
|  |  | Yuzu lactone | 197.15 | 6.77 | 1.29 | 0.88 | < 0.001 | pos |
|  |  | Alpha-methylphenylalanine | 180.10 | 3.65 | 1.23 | 0.91 | < 0.001 | pos |
|  |  | Coriandrone C | 264.09 | 3.02 | 1.19 | 0.90 | < 0.001 | pos |
|  |  | Norpropoxyphene | 370.20 | 7.44 | 2.07 | 2.55 | < 0.001 | neg |
|  |  | 6-[(1Z)-2-hydroxy-3-oxobut-1-en-1-yl]-7-methoxy-2H-chromen-2-one | 305.07 | 4.32 | 1.24 | 1.22 | 0.011 | neg |
| Benzenoids | Benzoic acids and derivatives | Antibiotic SB 202742 | 393.24 | 9.47 | 1.97 | 1.62 | < 0.001 | pos |
|  |  | 3,5-Bis(1,1-dimethylethyl)-4-hydroxy-benzoic acid ethyl ester | 573.34 | 8.00 | 1.59 | 1.66 | 0.001 | neg |
|  |  | Salicylic acid | 137.02 | 4.65 | 1.04 | 1.07 | < 0.001 | neg |
|  | Aniline and substituted anilines | OR-1855 | 204.11 | 2.94 | 1.51 | 0.64 | 0.013 | pos |
|  | Anisoles | 1-(4-methoxyphenyl)-4-methylpentan-3-ol | 461.29 | 8.43 | 1.12 | 0.76 | 0.004 | neg |
|  | Benzenediols | (Z, Z)-2-Methyl-5-(8,11,14-pentadecatrienyl)-1,3-benzenediol | 392.25 | 7.10 | 1.09 | 1.14 | 0.023 | pos |
|  |  |  |  |  |  |  |  |  |
| **Table S5** **(continued)** | | | | | | | | |
| **HMDB Superclass** | **HMDB Subclass** | **Metabolite** | **M/Z** | **Retention time** | **VIP** | **FC(L/C)** | ***P*-value** | **Mode** |
|  |  | Pyrocatechol | 109.03 | 3.14 | 2.08 | 0.54 | 0.004 | neg |
|  | Indanones | Pterosin N | 279.12 | 3.41 | 1.09 | 0.81 | 0.004 | neg |
|  | Methoxybenzenes | Homoveratric acid | 195.07 | 3.72 | 1.06 | 1.26 | 0.007 | neg |
|  | Unclassified | 2-Phenoxyethanol | 180.10 | 3.16 | 1.19 | 0.93 | < 0.001 | pos |
|  |  | 1-Phenylethanol | 121.07 | 4.20 | 1.74 | 1.43 | < 0.001 | neg |
|  |  | 5-Phenyl-1,3-oxazinane-2,4-dione | 226.03 | 4.40 | 1.30 | 1.11 | 0.003 | neg |
| Alkaloids and derivatives | Unclassified | Harmalol | 181.08 | 3.35 | 1.24 | 0.89 | < 0.001 | neg |
| Organosulfur compounds | Aryl thioethers | Phenyl vinyl sulfide | 181.03 | 6.43 | 1.91 | 0.46 | 0.001 | neg |
| Hydrocarbon derivatives | Tropolones | Beta-Thujaplicin | 209.08 | 3.69 | 1.04 | 0.77 | 0.043 | neg |
| Others |  | 15(R)-15-methyl Prostaglandin A2 | 349.24 | 4.87 | 3.16 | 2.17 | < 0.001 | pos |
|  |  | (3beta,5xi,9xi,18xi) -Olean-12-en-28-oic acid,3-hydroxy- | 439.36 | 9.22 | 2.90 | 1.43 | < 0.001 | pos |
|  |  | 2-aminohexadecanoic acid | 272.26 | 7.49 | 1.92 | 1.42 | < 0.001 | pos |
|  |  | 2-oxo-pentadecanoic acid | 239.20 | 8.05 | 1.78 | 1.21 | < 0.001 | pos |
|  |  | Oleoyl dopamine | 418.33 | 8.58 | 1.71 | 1.19 | < 0.001 | pos |
|  |  | 4-formyl Indole | 146.06 | 2.98 | 1.68 | 1.32 | 0.001 | pos |
|  |  | 7alpha,12alpha,24-trihydroxycholest-4-en-3-one | 433.33 | 7.78 | 1.60 | 1.21 | < 0.001 | pos |
|  |  | 16,16-dimethyl-PGE1 | 383.28 | 7.32 | 1.59 | 1.19 | < 0.001 | pos |
|  |  | 3,5,7-Trimethyl-2E,4E,6E,8E-decatetraene | 177.16 | 8.85 | 1.58 | 1.17 | < 0.001 | pos |
|  |  | Hydroxy-gamma-sanshool | 290.21 | 4.42 | 1.55 | 0.78 | 0.034 | pos |
|  |  | 1alpha,25-dihydroxy-26,27-ethanovitamin D3 | 443.35 | 8.68 | 1.49 | 1.11 | < 0.001 | pos |
|  |  | PG (16:0/0:0) [U] | 467.28 | 10.18 | 1.46 | 1.09 | 0.001 | pos |
|  |  | 1,8-Diazacyclotetradecane-2,9-dione | 227.17 | 2.88 | 1.43 | 1.18 | 0.022 | pos |
|  |  | Nuatigenin | 431.32 | 8.03 | 1.35 | 1.12 | < 0.001 | pos |
|  |  | Pfaffic acid | 441.34 | 9.13 | 1.33 | 1.08 | 0.001 | pos |
|  |  | Vitamin D2 (Ergocalciferol) | 397.35 | 9.13 | 1.32 | 1.10 | 0.001 | pos |
|  |  | 9-hydroxy-10-oxo-12(Z)-octadecenoic acid | 335.22 | 6.68 | 1.26 | 1.05 | < 0.001 | pos |
|  |  | Coumestrol | 269.04 | 5.34 | 1.25 | 1.18 | 0.002 | pos |
|  |  | Orcinol | 125.06 | 2.67 | 1.25 | 1.16 | 0.005 | pos |
|  |  | Alpha-9(10)-EpODE | 295.23 | 6.49 | 1.22 | 1.06 | < 0.001 | pos |
|  |  | (±)12,13-DiHOME | 337.23 | 6.76 | 1.20 | 1.05 | < 0.001 | pos |
|  |  |  |  |  |  |  |  |  |
| **Table S5** **(continued)** | | | | | | | | |
| **HMDB Superclass** | **HMDB Subclass** | **Metabolite** | **M/Z** | **Retention time** | **VIP** | **FC(L/C)** | ***P*-value** | **Mode** |
|  |  | Amprotropine | 308.22 | 4.74 | 1.20 | 1.08 | 0.001 | pos |
|  |  | Tetranor-12(R)-HETE | 267.19 | 5.89 | 1.11 | 1.07 | < 0.001 | pos |
|  |  | 9,10-epoxy-13-hydroxy-11-octadecenoic acid | 313.24 | 6.49 | 1.04 | 1.04 | < 0.001 | pos |
|  |  | TG (12:0/13:0/20:5(5Z,8Z,11Z,14Z,17Z))[iso6] | 755.61 | 11.02 | 1.03 | 1.15 | 0.009 | pos |
|  |  | 13Z-Docosenamide | 338.34 | 10.15 | 1.00 | 1.04 | 0.035 | pos |
|  |  | 6-hydroxysphingosine | 316.28 | 5.81 | 2.52 | 0.68 | < 0.001 | pos |
|  |  | NORELEAGNINE | 173.11 | 3.22 | 2.50 | 0.50 | < 0.001 | pos |
|  |  | 8-Isoquinoline methanamine (hydrochloride) | 158.10 | 3.33 | 2.42 | 0.71 | < 0.001 | pos |
|  |  | Docosahexaenoyl Glycine | 386.27 | 9.00 | 2.06 | 0.53 | < 0.001 | pos |
|  |  | 3-Methyloxyindole | 148.08 | 4.43 | 1.62 | 0.85 | 0.009 | pos |
|  |  | 1-ACETYLPIPERIDINE | 128.11 | 3.56 | 1.60 | 1.28 | 0.027 | pos |
|  |  | Quadrone | 249.15 | 6.22 | 1.58 | 0.81 | < 0.001 | pos |
|  |  | Cis-5-dodecenoic acid | 199.17 | 6.74 | 1.48 | 0.84 | < 0.001 | pos |
|  |  | (±)-Equol | 243.10 | 5.11 | 1.44 | 0.88 | < 0.001 | pos |
|  |  | Malvidin | 331.08 | 5.08 | 1.42 | 1.18 | 0.008 | pos |
|  |  | 2,3,5-Trimethacarb | 194.12 | 3.51 | 1.41 | 0.89 | < 0.001 | pos |
|  |  | R-Palmitoyl-(2-methyl) Ethanolamide | 314.30 | 7.48 | 1.37 | 1.13 | 0.001 | pos |
|  |  | Lumichrome | 526.18 | 4.17 | 1.34 | 1.15 | 0.001 | pos |
|  |  | PE (16:1(5Z)/16:1(5Z)) | 688.49 | 10.81 | 1.27 | 0.91 | 0.001 | pos |
|  |  | Confertifoline | 235.17 | 6.64 | 1.23 | 0.91 | < 0.001 | pos |
|  |  | Farnesyl acetone | 263.24 | 11.53 | 1.16 | 0.91 | 0.002 | pos |
|  |  | 9S,10R-Epoxy-6Z-octadecene | 570.54 | 12.18 | 1.14 | 0.92 | 0.001 | pos |
|  |  | PG (17:1(9Z)/0:0) | 497.29 | 10.39 | 1.10 | 1.10 | 0.018 | pos |
|  |  | 9,10-Epoxy-18-hydroxystearate | 315.25 | 8.36 | 1.10 | 0.93 | < 0.001 | pos |
|  |  | PE (16:0/0:0) | 454.29 | 8.59 | 1.07 | 1.06 | 0.007 | pos |
|  |  | (+)-Prosopinine | 314.27 | 4.51 | 1.06 | 0.93 | < 0.001 | pos |
|  |  | C-2 Ceramide | 342.30 | 9.05 | 1.03 | 0.93 | < 0.001 | pos |
|  |  | Bifemelane (M4) | 269.12 | 5.13 | 1.00 | 0.96 | < 0.001 | pos |
|  |  | P-Salicylic acid | 137.02 | 4.20 | 1.14 | 1.09 | < 0.001 | neg |
|  |  | MEDICA 16 | 341.27 | 8.94 | 1.02 | 1.08 | < 0.001 | neg |
|  |  | Leu-Trp-OH | 424.15 | 6.84 | 1.43 | 1.82 | 0.010 | neg |
|  |  | SIMMONDSIN-2'-FERULATE | 550.19 | 8.11 | 1.18 | 0.86 | < 0.001 | neg |
| Diets: C, corn and corn silage diet; L, sugar beet pulp and alfalfa silage diet; HMDB, human metabolome database; M/Z, mass-to-charge ratio; FC, fold change; VIP, variable importance in the projection; pos, in positive ion mode; neg, in negative ion mode. | | | | | | | | |

| **Table S6 Significantly differential metabolites between the glucogenic diet S and lipogenic diet L after 48 h *in vitro* fermentation with rumen fluid of dairy cows** | | | | | | | | | | | | | | | | |
| --- | --- | --- | --- | --- | --- | --- | --- | --- | --- | --- | --- | --- | --- | --- | --- | --- |
| **HMDB Superclass** | **HMDB Subclass** | | **Metabolite** | | **M/Z** | | **Retention time** | | **VIP** | | **FC(L/S)** | | ***P*-value** | | **Mode** | |
| Lipids and lipid-like molecules | | Fatty acids and conjugates | | 13-hydroxyoctadecanoic acid | | 283.26 | | 9.43 | | 1.62 | | 0.71 | | 0.005 | | pos |
|  |  |  | | Petroselinic acid | | 283.26 | | 10.95 | | 1.13 | | 0.94 | | < 0.001 | | pos |
|  |  |  | | Myristoleic acid | | 191.18 | | 9.14 | | 2.89 | | 1.69 | | < 0.001 | | pos |
|  | |  | | Goshuyic acid | | 247.17 | | 9.13 | | 2.36 | | 1.68 | | < 0.001 | | pos |
|  | |  | | 12,15-Epoxy-13,14-dimethyleicosa-12,14,16-trienoic acid | | 349.27 | | 8.08 | | 1.79 | | 1.58 | | 0.003 | | pos |
|  | |  | | Stearic acid | | 307.26 | | 7.14 | | 1.50 | | 1.13 | | < 0.001 | | pos |
|  | |  | | 2-Octenoic acid | | 302.23 | | 6.36 | | 1.47 | | 1.26 | | 0.002 | | pos |
|  | |  | | 5-Hexyl-2-furanhexanoic acid | | 289.18 | | 5.85 | | 1.41 | | 1.12 | | 0.000 | | pos |
|  | |  | | Dioscoretine | | 242.17 | | 4.93 | | 1.35 | | 1.14 | | 0.002 | | pos |
|  | |  | | Docosatrienoic acid | | 357.28 | | 8.56 | | 1.24 | | 1.26 | | 0.014 | | pos |
|  | |  | | 3,3-Dimethylglutaric acid | | 159.07 | | 3.81 | | 1.39 | | 0.84 | | < 0.001 | | neg |
|  | |  | | (R)-3-Hydroxy-Octadecanoic acid | | 345.26 | | 9.04 | | 1.20 | | 0.65 | | 0.003 | | neg |
|  | |  | | 6-Ketomyristic acid | | 241.18 | | 7.68 | | 1.09 | | 0.90 | | < 0.001 | | neg |
|  | |  | | 2-Octenedioic acid | | 217.07 | | 2.24 | | 2.29 | | 3.12 | | < 0.001 | | neg |
|  | |  | | 2-hydroxyhexadecanoic acid | | 271.23 | | 9.10 | | 1.52 | | 1.11 | | < 0.001 | | neg |
|  | |  | | Nonate | | 233.10 | | 2.88 | | 1.23 | | 1.19 | | < 0.001 | | neg |
|  | |  | | 5-Tetradecenoic acid | | 271.19 | | 7.16 | | 1.18 | | 1.24 | | < 0.001 | | neg |
|  | |  | | 2-Hydroxymyristic Acid | | 243.20 | | 8.45 | | 1.11 | | 1.09 | | 0.001 | | neg |
|  | |  | | 7Z,10Z-Hexadecadienoic acid | | 297.21 | | 6.94 | | 1.08 | | 1.15 | | < 0.001 | | neg |
|  | |  | | (S)-10,16-Dihydroxyhexadecanoic acid | | 287.22 | | 7.97 | | 1.07 | | 1.11 | | 0.001 | | neg |
|  | | Fatty acid esters | | 4,8 dimethylnonanoyl carnitine | | 330.26 | | 8.46 | | 1.10 | | 0.92 | | < 0.001 | | pos |
|  | |  | | Butyl 2-decenoate | | 191.18 | | 9.22 | | 3.03 | | 1.94 | | < 0.001 | | pos |
|  | |  | | Propyl 2,4-decadienoate | | 211.17 | | 3.86 | | 1.01 | | 1.05 | | < 0.001 | | pos |
|  | |  | | Oleoylcarnitine | | 462.30 | | 8.73 | | 1.02 | | 0.89 | | 0.019 | | neg |
|  | |  | | Stearoyllactic acid | | 355.28 | | 9.88 | | 2.27 | | 2.39 | | < 0.001 | | neg |
|  | |  | | Ethyl (Z, Z)-5,8-tetradecadienoate | | 297.21 | | 6.53 | | 1.19 | | 1.14 | | < 0.001 | | neg |
|  | | Triterpenoids | | Tomentosolic acid | | 437.34 | | 9.48 | | 3.22 | | 1.60 | | < 0.001 | | pos |
|  | |  | | Ursolic acid | | 457.37 | | 8.90 | | 3.10 | | 1.54 | | < 0.001 | | pos |
|  | |  | | Camelledionol | | 423.33 | | 8.71 | | 2.98 | | 1.52 | | < 0.001 | | pos |
|  | |  | | Oleanolic acid | | 457.37 | | 9.05 | | 2.86 | | 1.66 | | < 0.001 | | pos |
|  | |  | | Sandosapogenol | | 439.36 | | 8.90 | | 2.72 | | 1.33 | | < 0.001 | | pos |
|  | |  | | (3beta,17alpha,23S,24S)-17,23-Epoxy-3,24,29-trihydroxy-27-norlanost-8-en-15-one | | 457.33 | | 7.07 | | 2.51 | | 1.50 | | < 0.001 | | pos |
|  | |  | |  | |  | |  | |  | |  | |  | |  |
| **Table S6 (continued)** | | | | | | | | | | | | | | | | |
| **HMDB Superclass** | **HMDB Subclass** | | **Metabolite** | | **M/Z** | | **Retention time** | | **VIP** | | **FC(L/S)** | | ***P*-value** | | **Mode** | |
|  | |  | | Asperagenin | | 449.33 | | 7.19 | | 1.83 | | 1.17 | | < 0.001 | | pos |
|  | |  | | Lucidenic acid E2 | | 539.26 | | 9.24 | | 1.57 | | 1.10 | | < 0.001 | | pos |
|  | |  | | Gamma-Taraxastane-3,20-diol | | 427.39 | | 10.37 | | 1.43 | | 1.11 | | 0.008 | | pos |
|  | |  | | Glyuranolide | | 530.34 | | 10.24 | | 1.32 | | 1.11 | | 0.005 | | pos |
|  | |  | | Ganoderic acid F | | 593.28 | | 9.06 | | 1.23 | | 1.06 | | 0.002 | | pos |
|  | |  | | Ganoderenic acid E | | 551.27 | | 8.85 | | 1.20 | | 1.05 | | < 0.001 | | pos |
|  | |  | | Glabric acid | | 531.33 | | 6.84 | | 2.61 | | 1.77 | | < 0.001 | | neg |
|  | |  | | Ganoderic acid A | | 497.29 | | 8.39 | | 2.58 | | 2.06 | | < 0.001 | | neg |
|  | |  | | Ganoderiol I | | 501.36 | | 9.14 | | 2.43 | | 1.37 | | < 0.001 | | neg |
|  | |  | | (3beta,19alpha)-3,19,23,24-Tetrahydroxy-12-oleanen-28-oic acid | | 539.33 | | 6.96 | | 1.69 | | 1.21 | | < 0.001 | | neg |
|  | |  | | Medicagenic acid | | 547.33 | | 7.39 | | 1.48 | | 1.32 | | 0.004 | | neg |
|  | |  | | Camellenodiol | | 463.34 | | 9.97 | | 1.16 | | 1.20 | | 0.001 | | neg |
|  | | Sesquiterpenoids | | 7(14)-Bisabolene-2,3,10,11-tetrol | | 255.19 | | 6.43 | | 1.99 | | 0.68 | | < 0.001 | | pos |
|  | |  | | Deoxynivalenol 3-glucoside | | 491.21 | | 9.33 | | 1.09 | | 0.90 | | 0.018 | | pos |
|  | |  | | S-Japonin | | 378.21 | | 9.79 | | 1.01 | | 0.91 | | 0.006 | | pos |
|  | |  | | Ipomeatetrahydrofuran | | 239.20 | | 7.87 | | 2.30 | | 1.42 | | < 0.001 | | pos |
|  | |  | | Auberganol | | 241.22 | | 8.03 | | 1.79 | | 1.26 | | < 0.001 | | pos |
|  | |  | | 4,7-Megastigmadien-9-ol | | 411.33 | | 8.63 | | 1.38 | | 1.08 | | < 0.001 | | pos |
|  | |  | | Sterebin A | | 343.25 | | 5.87 | | 1.20 | | 1.06 | | < 0.001 | | pos |
|  | |  | | (1(10)E,4a,5E)-1(10),5-Germacradiene-12-acetoxy-4,11-diol | | 295.19 | | 7.05 | | 1.77 | | 1.80 | | 0.001 | | neg |
|  | |  | | 7(14)-Farnesene-9,12-diol | | 287.22 | | 7.80 | | 1.32 | | 1.32 | | < 0.001 | | neg |
|  | |  | | Guaidiol | | 283.19 | | 6.38 | | 1.03 | | 1.13 | | 0.001 | | neg |
|  | | Terpene lactones | | Isoalantolactone | | 233.15 | | 6.28 | | 1.35 | | 0.92 | | 0.000 | | pos |
|  | |  | | 8-Deoxy-11,13-dihydroxygrosheimin | | 298.18 | | 4.86 | | 1.35 | | 0.84 | | 0.010 | | pos |
|  | |  | | Tatridin B | | 229.12 | | 6.15 | | 1.32 | | 0.90 | | < 0.001 | | pos |
|  | |  | | 2alpha-Hydroxyalantolactone | | 229.12 | | 7.92 | | 1.81 | | 0.68 | | < 0.001 | | neg |
|  | |  | | 4,11,13,15-Tetrahydroridentin B | | 249.15 | | 7.03 | | 1.52 | | 0.76 | | < 0.001 | | neg |
|  | |  | | Tavulin | | 245.12 | | 6.17 | | 1.33 | | 0.86 | | < 0.001 | | neg |
|  | |  | | Crispolide | | 261.11 | | 4.48 | | 1.24 | | 0.84 | | < 0.001 | | neg |
|  | |  | | Dihydrocumambrin A | | 307.16 | | 7.83 | | 2.94 | | 5.71 | | 0.001 | | neg |
|  | | Monoterpenoids | | Withangulatin A | | 549.25 | | 8.69 | | 2.64 | | 1.33 | | < 0.001 | | pos |
|  | |  | | Piperitone | | 170.15 | | 5.71 | | 1.70 | | 1.34 | | < 0.001 | | pos |
|  | |  | | Linalyl propionate | | 243.20 | | 5.46 | | 1.28 | | 1.25 | | 0.012 | | pos |
|  | |  | |  | |  | |  | |  | |  | |  | |  |
| **Table S6 (continued)** | | | | | | | | | | | | | | | | |
| **HMDB Superclass** | **HMDB Subclass** | | **Metabolite** | | **M/Z** | | **Retention time** | | **VIP** | | **FC(L/S)** | | ***P*-value** | | **Mode** | |
|  | |  | | 3-(5,6,6-Trimethylbicyclo [2.2.1] hept-1-yl)cyclohexanol | | 281.21 | | 7.58 | | 1.71 | | 1.43 | | < 0.001 | | neg |
|  | |  | | Valechlorin | | 457.17 | | 8.02 | | 1.63 | | 1.15 | | 0.002 | | neg |
|  | |  | | Soyasapogenol B 3-O-b-D-glucuronide | | 671.35 | | 7.95 | | 1.11 | | 1.14 | | 0.001 | | neg |
|  | | Steroid lactones | | Withaperuvin H | | 611.29 | | 8.17 | | 2.64 | | 1.45 | | 0.001 | | pos |
|  | |  | | Physagulin F | | 567.26 | | 8.21 | | 1.92 | | 1.19 | | < 0.001 | | pos |
|  | |  | | Physagulin C | | 565.24 | | 8.41 | | 1.54 | | 1.11 | | < 0.001 | | pos |
|  | |  | | Physapubenolide | | 565.25 | | 8.23 | | 1.53 | | 1.44 | | 0.001 | | neg |
|  | | Steroid lactones | | (5alpha,6beta,14alpha,20R,22R)-5,6,14,20,27-Pentahydroxy-1-oxowith-24-enolide | | 505.28 | | 5.66 | | 1.43 | | 1.20 | | < 0.001 | | neg |
|  | | Fatty alcohols | | 1-Undecanol | | 367.36 | | 10.50 | | 1.23 | | 1.47 | | 0.036 | | pos |
|  | |  | | Avocadene | | 304.29 | | 6.62 | | 1.02 | | 1.06 | | 0.012 | | pos |
|  | |  | | Avocadyne | | 329.23 | | 8.91 | | 1.14 | | 1.22 | | 0.002 | | neg |
|  | |  | | Oleyl alcohol | | 313.27 | | 9.57 | | 1.13 | | 1.10 | | < 0.001 | | neg |
|  | | Glycerophosphoethanolamines | | PE (14:1(9Z)/16:1(9Z)) | | 660.46 | | 10.33 | | 1.62 | | 0.75 | | 0.008 | | pos |
|  | |  | | LysoPE (0:0/16:0) | | 436.28 | | 8.40 | | 1.12 | | 0.87 | | 0.026 | | pos |
|  | |  | | PE (15:0/22:1(13Z)) | | 804.58 | | 11.72 | | 1.28 | | 0.81 | | 0.016 | | neg |
|  | |  | | 1-Heptadecanoylglycerophosphoethanolamine | | 512.30 | | 7.63 | | 1.09 | | 1.15 | | 0.007 | | neg |
|  | | Lineolic acids and derivatives | | Punicic acid | | 243.21 | | 7.35 | | 1.51 | | 0.85 | | < 0.001 | | pos |
|  | |  | | 13S-hydroxyoctadecadienoic acid | | 329.27 | | 9.87 | | 1.24 | | 0.89 | | < 0.001 | | pos |
|  | |  | | 2-Hydroxylinolenic acid | | 317.21 | | 7.35 | | 1.15 | | 0.90 | | < 0.001 | | pos |
|  | |  | | Cibaric acid | | 369.19 | | 6.18 | | 1.29 | | 1.20 | | < 0.001 | | neg |
|  | | Diradylglycerols | | DG (16:0/16:0/0:0) | | 591.50 | | 10.92 | | 1.20 | | 1.15 | | 0.048 | | pos |
|  | |  | | DG (20:1(11Z)/22:5(4Z,7Z,10Z,13Z,16Z)/0:0) | | 677.55 | | 9.98 | | 1.31 | | 0.78 | | 0.040 | | neg |
|  | |  | | DG (15:0/18:0/0:0) | | 627.52 | | 11.53 | | 1.21 | | 0.81 | | 0.006 | | neg |
|  | | Diterpenoids | | Beta-Tocopheryl quinone | | 433.37 | | 10.23 | | 1.60 | | 1.47 | | 0.043 | | pos |
|  | |  | | Austroinulin | | 355.28 | | 6.68 | | 1.33 | | 1.07 | | < 0.001 | | pos |
|  | |  | | Phytocassane B | | 317.21 | | 6.59 | | 1.31 | | 1.14 | | < 0.001 | | pos |
|  | | Fatty acyl glycosides | | 1-Octen-3-yl primeveroside | | 445.20 | | 7.93 | | 1.75 | | 0.80 | | 0.015 | | pos |
|  | |  | | 4-Hydroxyproline galactoside | | 258.10 | | 3.23 | | 1.51 | | 0.86 | | < 0.001 | | pos |
|  | |  | | 6S,9R-Dihydroxy-4,7E-megastigmadien-3-one 9-[apiosyl-(1->6)-glucoside] | | 563.23 | | 8.41 | | 1.40 | | 1.16 | | < 0.001 | | neg |
|  | | Bile acids, alcohols and derivatives | | 5beta-Cholestane-3alpha,7alpha,24,26-tetrol | | 401.34 | | 7.36 | | 1.14 | | 0.92 | | 0.008 | | pos |
|  | |  | | 27-Nor-5b-cholestane-3a,7a,12a,24,25-pentol | | 480.37 | | 9.34 | | 1.14 | | 0.90 | | 0.012 | | pos |
|  | |  | |  | |  | |  | |  | |  | |  | |  |
| **Table S6 (continued)** | | | | | | | | | | | | | | | | |
| **HMDB Superclass** | **HMDB Subclass** | | **Metabolite** | | **M/Z** | | **Retention time** | | **VIP** | | **FC(L/S)** | | ***P*-value** | | **Mode** | |
|  | | Cholestane steroids | | 5a-Cholest-8-en-3b-ol | | 409.35 | | 10.61 | | 1.27 | | 0.86 | | 0.003 | | pos |
|  | |  | | 5alpha-Cholestanone | | 811.68 | | 11.16 | | 1.22 | | 1.20 | | 0.014 | | pos |
|  | | Glycerophosphates | | LysoPA(P-16:0e/0:0) | | 417.24 | | 9.12 | | 1.55 | | 1.25 | | 0.001 | | pos |
|  | |  | | 1-(9Z-tetradecenoyl)-glycero-3-phosphate | | 379.19 | | 8.11 | | 1.80 | | 0.56 | | < 0.001 | | neg |
|  | | Glycerophosphocholines | | PC (16:0/18:2(9Z,12Z)) | | 802.56 | | 11.16 | | 1.25 | | 0.86 | | 0.008 | | neg |
|  | |  | | LysoPC (16:0) | | 540.33 | | 8.36 | | 1.06 | | 0.88 | | 0.011 | | neg |
|  | | Hydroxysteroids | | Tetrahydrodeoxycorticosterone | | 379.22 | | 8.98 | | 1.47 | | 1.15 | | < 0.001 | | pos |
|  | |  | | Prednisone | | 379.15 | | 8.92 | | 1.36 | | 0.83 | | 0.001 | | neg |
|  | | Pregnane steroids | | 17a-Hydroxypregnenolone | | 333.24 | | 5.75 | | 3.02 | | 1.80 | | < 0.001 | | pos |
|  | |  | | Halobetasol Propionate | | 521.13 | | 7.58 | | 2.13 | | 1.72 | | 0.001 | | neg |
|  | | Steroidal glycosides | | Corchoroside B | | 541.28 | | 9.01 | | 1.64 | | 1.14 | | < 0.001 | | pos |
|  | |  | | Torvoside D | | 747.39 | | 7.73 | | 2.24 | | 1.67 | | < 0.001 | | neg |
|  | | Terpene glycosides | | Lansioside A | | 642.44 | | 11.64 | | 1.24 | | 1.14 | | 0.019 | | pos |
|  | |  | | Calenduloside E | | 677.39 | | 7.77 | | 2.83 | | 2.77 | | < 0.001 | | neg |
|  | | Ceramides | | N-[(4E,8E)-1,3-dihydroxyoctadeca-4,8-dien-2-yl]hexadecanamide | | 580.49 | | 11.88 | | 1.15 | | 0.86 | | 0.018 | | neg |
|  | | Eicosanoids | | Carboprost Tromethamine | | 367.25 | | 7.62 | | 1.06 | | 0.87 | | < 0.001 | | neg |
|  | | Ergostane steroids | | Delta 8,14 -Sterol | | 393.35 | | 12.76 | | 1.38 | | 0.88 | | < 0.001 | | pos |
|  | | Estrane steroids | | 4-hydroxyestradiol | | 289.18 | | 5.25 | | 1.15 | | 1.08 | | < 0.001 | | pos |
|  | | Monoradylglycerols | | MG(i-12:0/0:0/0:0) | | 255.20 | | 8.07 | | 1.46 | | 1.17 | | < 0.001 | | neg |
|  | | Quinone and hydroquinone lipids | | 13'-Carboxy-alpha-tocopherol | | 493.39 | | 11.52 | | 1.62 | | 0.71 | | 0.001 | | pos |
|  | | Steroid esters | | Physapubescin | | 553.28 | | 7.95 | | 2.09 | | 1.22 | | < 0.001 | | pos |
|  | | Stigmastanes and derivatives | | 6-Deoxohomodolichosterone | | 463.38 | | 10.59 | | 1.63 | | 1.17 | | 0.003 | | pos |
| Organic acids and derivatives | | Amino acids, peptides, and analogues | | N6-Acetyl-5S-hydroxy-L-lysine | | 243.08 | | 4.68 | | 2.85 | | 0.37 | | < 0.001 | | pos |
|  |  |  | | Cytidine 2',3'-cyclic phosphate | | 350.01 | | 5.50 | | 1.68 | | 0.75 | | 0.014 | | pos |
|  |  |  | | Valyl-Glutamate | | 229.12 | | 1.00 | | 1.63 | | 0.86 | | < 0.001 | | pos |
|  | |  | | Frangulanine | | 483.34 | | 9.62 | | 1.55 | | 0.75 | | 0.010 | | pos |
|  | |  | | Isoleucyl-Glutamate | | 243.13 | | 1.41 | | 1.44 | | 0.90 | | < 0.001 | | pos |
|  | |  | | Tetrahydrodipicolinate | | 204.09 | | 1.57 | | 1.24 | | 0.88 | | < 0.001 | | pos |
|  | |  | | Phenylalanylproline | | 245.13 | | 3.54 | | 1.07 | | 0.93 | | < 0.001 | | pos |
|  | |  | | N-[[3-Hydroxy-2-(2-pentenyl) cyclopentyl] acetyl] isoleucine | | 308.22 | | 7.67 | | 2.39 | | 1.41 | | < 0.001 | | pos |
|  | |  | | 5-Hydroxyindoleacetylglycine | | 213.07 | | 3.25 | | 1.61 | | 1.24 | | < 0.001 | | pos |
|  | |  | |  | |  | |  | |  | |  | |  | |  |
| **Table S6 (continued)** | | | | | | | | | | | | | | | | |
| **HMDB Superclass** | **HMDB Subclass** | | **Metabolite** | | **M/Z** | | **Retention time** | | **VIP** | | **FC(L/S)** | | ***P*-value** | | **Mode** | |
|  | |  | | 2-Hepteneoylglycine | | 230.10 | | 3.07 | | 2.04 | | 0.43 | | < 0.001 | | neg |
|  | |  | | (1R)-Glutathionyl-(2R)-hydroxy-1,2-dihydronaphthalene | | 472.12 | | 5.47 | | 1.76 | | 0.55 | | 0.001 | | neg |
|  | |  | | N-(4-Hydroxycinnamoyl) tyrosine | | 362.08 | | 5.41 | | 1.73 | | 0.74 | | < 0.001 | | neg |
|  | |  | | Captopril-cysteine disulfide | | 371.05 | | 8.00 | | 1.61 | | 0.48 | | < 0.001 | | neg |
|  | |  | | Indolylacryloylglycine | | 225.07 | | 3.35 | | 1.47 | | 0.89 | | < 0.001 | | neg |
|  | |  | | N-Arachidonoyl glycine | | 406.26 | | 8.05 | | 1.25 | | 0.77 | | < 0.001 | | neg |
|  | |  | | 3-Hydroxymugineic acid | | 357.09 | | 5.55 | | 1.24 | | 0.80 | | 0.034 | | neg |
|  | |  | | Arginyl-Gamma-glutamate | | 283.15 | | 7.15 | | 1.16 | | 0.80 | | 0.001 | | neg |
|  | |  | | 4-Hydroxystachydrine | | 158.08 | | 3.22 | | 1.19 | | 1.31 | | 0.039 | | neg |
|  | |  | | Oleoyl glycine | | 384.27 | | 8.85 | | 1.08 | | 1.20 | | 0.003 | | neg |
|  | |  | | N-Acetylproline | | 138.06 | | 2.85 | | 1.08 | | 1.22 | | 0.003 | | neg |
|  | | Hybrid peptides | | Beta-Alanyl-L-lysine | | 476.32 | | 8.31 | | 1.48 | | 0.86 | | < 0.001 | | pos |
|  | |  | | Astin I | | 516.20 | | 2.93 | | 1.17 | | 1.10 | | 0.002 | | pos |
|  | | Medium-chain hydroxy acids and derivatives | | 12-Hydroxydodecanoic acid | | 258.21 | | 5.72 | | 1.34 | | 0.88 | | < 0.001 | | pos |
| Organoheterocyclic compounds | | Gamma butyrolactones | | 3-Hydroxyadipic acid 3,6-lactone | | 143.04 | | 1.62 | | 1.58 | | 0.61 | | 0.001 | | neg |
|  | |  | | Artabsinolide A | | 261.11 | | 6.13 | | 1.32 | | 0.69 | | 0.013 | | neg |
|  | |  | | 5-Nonyltetrahydro-2-oxo-3-furancarboxylic acid | | 237.15 | | 6.26 | | 1.17 | | 1.14 | | < 0.001 | | neg |
|  | | 1-benzopyrans | | Gamma-CEHC | | 263.13 | | 5.26 | | 1.33 | | 0.76 | | 0.001 | | neg |
|  | |  | | 9'-Carboxy-gamma-chromanol | | 421.26 | | 7.53 | | 1.22 | | 1.20 | | 0.000 | | neg |
|  | | Hydropyridines | | 5-(2-Furanyl)-1,2,3,4,5,6-hexahydro-7H-cyclopenta[b]pyridin-7-one | | 248.09 | | 3.96 | | 1.10 | | 0.75 | | 0.015 | | neg |
|  | |  | | 2-Hydroxypyridine | | 140.04 | | 2.51 | | 1.64 | | 1.69 | | < 0.001 | | neg |
|  | | Indolyl carboxylic acids and derivatives | | Indoleacetic acid | | 220.06 | | 3.70 | | 1.79 | | 0.46 | | 0.002 | | neg |
|  | |  | | 5-Hydroxyindoleacetic acid | | 190.05 | | 3.53 | | 1.14 | | 0.77 | | 0.029 | | neg |
|  | | Benzoxazinones | | (R)-2,7-Dihydroxy-2H-1,4-benzoxazin-3(4H)-one | | 380.11 | | 5.58 | | 1.76 | | 0.81 | | < 0.001 | | pos |
|  | | Benzylisoquinolines | | 8-Propanoylneosolaniol | | 473.16 | | 7.24 | | 1.71 | | 0.68 | | 0.018 | | neg |
|  | | Bilirubins | | D-Urobilin | | 609.27 | | 8.19 | | 2.50 | | 2.26 | | 0.002 | | neg |
|  | | Dibenzoxepines | | Doxepin N-oxide glucuronide | | 507.17 | | 7.28 | | 2.28 | | 2.29 | | 0.016 | | neg |
|  | | Indoles | | Indoleacrylic acid | | 188.07 | | 3.41 | | 1.46 | | 0.85 | | 0.000 | | pos |
|  | |  | |  | |  | |  | |  | |  | |  | |  |
| **Table S6 (continued)** | | | | | | | | | | | | | | | | |
| **HMDB Superclass** | **HMDB Subclass** | | **Metabolite** | | **M/Z** | | **Retention time** | | **VIP** | | **FC(L/S)** | | ***P*-value** | | **Mode** | |
|  | | Pterins and derivatives | | 2-Amino-4-oxo-6-(1',2'-dioxoprolyl)-7,8-dihydroxypteridine | | 288.03 | | 1.24 | | 1.16 | | 0.87 | | < 0.001 | | neg |
|  | | Pyranones and derivatives | | Erinapyrone A | | 187.06 | | 2.69 | | 1.27 | | 1.20 | | < 0.001 | | neg |
|  | | Unclassified | | Canescein | | 611.25 | | 8.45 | | 2.75 | | 1.76 | | 0.001 | | pos |
|  | |  | | (+)-2,3-Dihydro-3-methyl-1H-pyrrole | | 125.11 | | 1.14 | | 1.27 | | 1.10 | | 0.022 | | pos |
|  | |  | | 3alpha-Hydroxyoreadone | | 285.17 | | 3.39 | | 1.00 | | 1.05 | | < 0.001 | | pos |
|  | |  | | N-Carbamoyl glucuronide lorcaserin | | 454.17 | | 8.36 | | 1.28 | | 0.78 | | 0.003 | | neg |
|  | |  | | Jasmine ketolactone | | 253.11 | | 5.43 | | 1.40 | | 1.37 | | < 0.001 | | neg |
|  | |  | | 3-Pyridinebutanoic acid | | 210.08 | | 4.75 | | 1.28 | | 1.26 | | < 0.001 | | neg |
| Organic oxygen compounds | | Carbohydrates and carbohydrate conjugates | | Indican | | 296.11 | | 2.97 | | 2.76 | | 0.69 | | < 0.001 | | pos |
|  | |  | | N-Acetylneuraminic acid | | 274.09 | | 2.80 | | 1.90 | | 0.68 | | 0.001 | | pos |
|  | |  | | (2S)-2-Butanol O-[b-D-Apiofuranosyl-(1->6)-b-D-glucopyranoside] | | 410.20 | | 8.36 | | 1.18 | | 0.91 | | < 0.001 | | pos |
|  | |  | | Ethyl glucuronide | | 187.06 | | 3.10 | | 1.01 | | 0.92 | | 0.002 | | pos |
|  | |  | | 6-(5-ethyl-2,3-dihydroxyphenoxy)-3,4,5-trihydroxyoxane-2-carboxylic acid | | 295.08 | | 3.42 | | 2.02 | | 1.52 | | 0.001 | | pos |
|  | |  | | 4-Methoxybenzyl O-(2-sulfoglucoside) | | 345.06 | | 6.38 | | 1.57 | | 1.48 | | 0.022 | | pos |
|  | |  | | Arbutin | | 317.06 | | 9.96 | | 1.42 | | 1.13 | | 0.000 | | pos |
|  | |  | | 3,4,5-trihydroxy-6-[(4-methoxy-1-benzofuran-6-yl) oxy] oxane-2-carboxylic acid | | 363.07 | | 6.81 | | 1.39 | | 1.23 | | 0.048 | | pos |
|  | |  | | Dihydromaleimide beta-D-glucoside | | 260.08 | | 1.40 | | 2.87 | | 0.25 | | < 0.001 | | neg |
|  | |  | | Tyramine glucuronide | | 294.10 | | 2.97 | | 2.32 | | 0.71 | | < 0.001 | | neg |
|  | |  | | Pisatoside | | 260.08 | | 1.63 | | 2.22 | | 0.61 | | < 0.001 | | neg |
|  | |  | | Fluoxetine glucuronide | | 520.14 | | 7.36 | | 2.14 | | 0.44 | | < 0.001 | | neg |
|  | |  | | 3-oxo-3-[(3,4,5,6-tetrahydroxyoxan-2-yl) methoxy] propanoic acid | | 247.05 | | 2.90 | | 1.57 | | 0.76 | | < 0.001 | | neg |
|  | |  | | 6-Sinapoylglucoraphenin | | 640.08 | | 0.83 | | 1.21 | | 0.91 | | < 0.001 | | neg |
|  | | Carbonyl compounds | | 3-Propyl-1,2-cyclopentanedione | | 182.12 | | 4.51 | | 1.66 | | 0.57 | | 0.032 | | pos |
|  | |  | | 1H-Pyrrole-2-carboxaldehyde | | 96.04 | | 0.71 | | 1.29 | | 1.11 | | < 0.001 | | pos |
|  | |  | | (3R,8E)-3-Hydroxy-5,8-megastigmadien-7-one | | 209.15 | | 4.35 | | 1.02 | | 1.06 | | 0.002 | | pos |
|  | |  | | 1-Hydroxyepiacorone | | 289.12 | | 3.60 | | 1.00 | | 0.85 | | 0.007 | | neg |
|  | |  | | 3-Methylcyclopentadecanone | | 283.23 | | 8.91 | | 1.15 | | 1.16 | | 0.001 | | neg |
|  | | Ethers | | Digoxigenin monodigitoxoside | | 565.28 | | 8.94 | | 1.42 | | 1.09 | | < 0.001 | | pos |
|  | |  | |  | |  | |  | |  | |  | |  | |  |
| **Table S6 (continued)** | | | | | | | | | | | | | | | | |
| **HMDB Superclass** | **HMDB Subclass** | | **Metabolite** | | **M/Z** | | **Retention time** | | **VIP** | | **FC(L/S)** | | ***P*-value** | | **Mode** | |
| Phenylpropanoids and polyketides | | Furanocoumarins | | (R)-Heraclenol 2'-(3-methyl-2-butenoate) | | 385.13 | | 7.70 | | 1.06 | | 0.76 | | 0.016 | | neg |
|  | |  | | Edulisin I | | 525.15 | | 8.03 | | 1.43 | | 1.21 | | 0.004 | | neg |
|  | | Cyclic diarylheptanoids | | Myricatomentoside II | | 571.16 | | 8.02 | | 1.56 | | 1.31 | | 0.003 | | neg |
|  | | Flavans | | 2-phenyl-3,4-dihydro-2H-1-benzopyran-3,5,7-triol | | 259.10 | | 3.88 | | 1.48 | | 0.76 | | 0.007 | | pos |
|  | | Flavonoid glycosides | | Delphinidin 3-rutinoside | | 594.16 | | 7.01 | | 1.24 | | 0.83 | | 0.001 | | pos |
|  | | Pyranoflavonoids | | Cycloartocarpin | | 479.17 | | 7.66 | | 1.90 | | 1.49 | | < 0.001 | | neg |
|  | | Unclassified | | 3-(1,2-dihydroxybutyl)-7-hydroxy-1H-isochromen-1-one | | 268.12 | | 4.98 | | 1.30 | | 0.68 | | 0.049 | | pos |
|  | |  | | Coriandrone C | | 264.09 | | 3.02 | | 1.21 | | 0.90 | | < 0.001 | | pos |
|  | |  | | Yuzu lactone | | 197.15 | | 6.77 | | 1.20 | | 0.90 | | < 0.001 | | pos |
|  | |  | | Alpha-methylphenylalanine | | 180.10 | | 3.65 | | 1.15 | | 0.92 | | < 0.001 | | pos |
|  | |  | | 3-(3,4-Dihydroxyphenyl)-2-methylpropionic acid | | 195.07 | | 5.42 | | 1.36 | | 0.87 | | < 0.001 | | neg |
|  | |  | | Norpropoxyphene | | 370.20 | | 7.44 | | 1.90 | | 1.92 | | 0.003 | | neg |
|  | |  | | 6-[(1Z)-2-hydroxy-3-oxobut-1-en-1-yl]-7-methoxy-2H-chromen-2-one | | 305.07 | | 4.32 | | 1.68 | | 1.32 | | 0.002 | | neg |
| Benzenoids | | Lineolic acids and derivatives | | Antibiotic SB 202742 | | 393.24 | | 9.47 | | 1.92 | | 1.49 | | < 0.001 | | pos |
|  | |  | | 3,5-Bis(1,1-dimethylethyl)-4-hydroxy-benzoic acid ethyl ester | | 573.34 | | 8.00 | | 1.67 | | 1.69 | | 0.001 | | neg |
|  | |  | | Salicylic acid | | 137.02 | | 4.65 | | 1.07 | | 1.06 | | < 0.001 | | neg |
|  | | Benzenediols | | (Z, Z)-2-Methyl-5-(8,11,14-pentadecatrienyl)-1,3-benzenediol | | 392.25 | | 7.10 | | 1.17 | | 1.12 | | 0.002 | | pos |
|  | |  | | 3-(3,4-dihydroxyphenyl)-N-[2-(4-hydroxyphenyl) ethyl] propanimidic acid | | 338.08 | | 1.28 | | 1.01 | | 0.89 | | < 0.001 | | neg |
|  | | Indanones | | Pterosin N | | 279.12 | | 3.41 | | 1.03 | | 0.83 | | 0.011 | | neg |
|  | | Unclassified | | 1-Phenylethanol | | 121.07 | | 4.20 | | 1.78 | | 1.38 | | < 0.001 | | neg |
| Organosulfur compounds | | Aryl thioethers | | Phenyl vinyl sulfide | | 181.03 | | 6.43 | | 1.55 | | 0.56 | | 0.015 | | neg |
| Nucleosides, nucleotides, and analogues | | Unclassified | | 1-Methylinosine | | 606.22 | | 10.46 | | 1.11 | | 1.16 | | 0.020 | | pos |
| Alkaloids and derivatives | | Unclassified | | Harmalol | | 181.08 | | 3.35 | | 1.32 | | 0.89 | | < 0.001 | | neg |
|  | |  | |  | |  | |  | |  | |  | |  | |  |
| **Table S6 (continued)** | | | | | | | | | | | | | | | | |
| **HMDB Superclass** | **HMDB Subclass** | | **Metabolite** | | **M/Z** | | **Retention time** | | **VIP** | | **FC(L/S)** | | ***P*-value** | | **Mode** | |
| Others | |  | | 6-hydroxysphingosine | | 316.28 | | 5.81 | | 2.55 | | 0.70 | | < 0.001 | | pos |
|  | |  | | 8-Isoquinoline methanamine (hydrochloride) | | 158.10 | | 3.33 | | 2.38 | | 0.73 | | < 0.001 | | pos |
|  | |  | | Docosahexaenoyl Glycine | | 386.27 | | 9.00 | | 2.20 | | 0.53 | | < 0.001 | | pos |
|  | |  | | NORELEAGNINE | | 173.11 | | 3.22 | | 2.09 | | 0.58 | | 0.002 | | pos |
|  | |  | | Hydroxy-gamma-sanshool | | 290.21 | | 4.42 | | 1.71 | | 0.78 | | 0.014 | | pos |
|  | |  | | Cis-5-dodecenoic acid | | 199.17 | | 6.74 | | 1.57 | | 0.84 | | < 0.001 | | pos |
|  | |  | | Quadrone | | 249.15 | | 6.22 | | 1.51 | | 0.84 | | < 0.001 | | pos |
|  | |  | | (±)-Equol | | 243.10 | | 5.11 | | 1.41 | | 0.89 | | < 0.001 | | pos |
|  | |  | | 2,3,5-Trimethacarb | | 194.12 | | 3.51 | | 1.38 | | 0.90 | | < 0.001 | | pos |
|  | |  | | 9S,10R-Epoxy-6Z-octadecene | | 570.54 | | 12.18 | | 1.29 | | 0.92 | | 0.001 | | pos |
|  | |  | | 12-SAHSA | | 589.52 | | 13.60 | | 1.27 | | 0.93 | | 0.003 | | pos |
|  | |  | | PE (16:1(5Z)/16:1(5Z)) | | 688.49 | | 10.81 | | 1.26 | | 0.91 | | 0.007 | | pos |
|  | |  | | Cer(d14:2(4E,6E)/16:0) | | 480.44 | | 10.40 | | 1.23 | | 0.91 | | 0.004 | | pos |
|  | |  | | Confertifoline | | 235.17 | | 6.64 | | 1.21 | | 0.92 | | < 0.001 | | pos |
|  | |  | | (+)-Prosopinine | | 314.27 | | 4.51 | | 1.12 | | 0.93 | | < 0.001 | | pos |
|  | |  | | 1-Palmitoyl-2-linoleoyl PE | | 716.52 | | 11.28 | | 1.11 | | 0.94 | | 0.008 | | pos |
|  | |  | | Bifemelane (M5) | | 287.13 | | 5.24 | | 1.08 | | 0.83 | | 0.020 | | pos |
|  | |  | | 15(R)-15-methyl Prostaglandin A2 | | 349.24 | | 4.87 | | 3.30 | | 2.11 | | < 0.001 | | pos |
|  | |  | | (3beta,5xi,9xi,18xi)-Olean-12-en-28-oic acid,3-hydroxy- | | 439.36 | | 9.22 | | 3.05 | | 1.43 | | < 0.001 | | pos |
|  | |  | | Fenirofibrate | | 321.09 | | 4.08 | | 2.51 | | 2.60 | | 0.017 | | pos |
|  | |  | | 2-aminohexadecanoic acid | | 272.26 | | 7.49 | | 2.12 | | 1.48 | | < 0.001 | | pos |
|  | |  | | 3,5,7-Trimethyl-2E,4E,6E,8E-decatetraene | | 177.16 | | 8.85 | | 1.79 | | 1.20 | | < 0.001 | | pos |
|  | |  | | 2-oxo-pentadecanoic acid | | 239.20 | | 8.05 | | 1.77 | | 1.19 | | < 0.001 | | pos |
|  | |  | | Amprotropine | | 308.22 | | 4.74 | | 1.70 | | 1.15 | | < 0.001 | | pos |
|  | |  | | 16,16-dimethyl-PGE1 | | 383.28 | | 7.32 | | 1.69 | | 1.20 | | < 0.001 | | pos |
|  | |  | | 1alpha,25-dihydroxy-26,27-ethanovitamin D3 | | 443.35 | | 8.68 | | 1.68 | | 1.13 | | < 0.001 | | pos |
|  | |  | | Lumichrome | | 526.18 | | 4.17 | | 1.67 | | 1.20 | | < 0.001 | | pos |
|  | |  | | Oleoyl dopamine | | 418.33 | | 8.58 | | 1.67 | | 1.19 | | 0.002 | | pos |
|  | |  | | 7alpha,12alpha,24-trihydroxycholest-4-en-3-one | | 433.33 | | 7.78 | | 1.54 | | 1.18 | | < 0.001 | | pos |
|  | |  | | Pfaffic acid | | 441.34 | | 9.13 | | 1.52 | | 1.09 | | < 0.001 | | pos |
|  | |  | | Vitamin D2 (Ergocalciferol) | | 397.35 | | 9.13 | | 1.51 | | 1.11 | | < 0.001 | | pos |
|  | |  | | 4-formyl Indole | | 146.06 | | 2.98 | | 1.48 | | 1.22 | | 0.001 | | pos |
|  | |  | | Malvidin | | 331.08 | | 5.08 | | 1.46 | | 1.18 | | 0.008 | | pos |
|  | |  | |  | |  | |  | |  | |  | |  | |  |
| **Table S6 (continued)** | | | | | | | | | | | | | | | | |
| **HMDB Superclass** | **HMDB Subclass** | | **Metabolite** | | **M/Z** | | **Retention time** | | **VIP** | | **FC(L/S)** | | ***P*-value** | | **Mode** | |
|  | |  | | PG(16:0/0:0)[U] | | 467.28 | | 10.18 | | 1.42 | | 1.08 | | 0.001 | | pos |
|  | |  | | Alpha-Vetivone | | 219.17 | | 10.05 | | 1.40 | | 1.15 | | 0.049 | | pos |
|  | |  | | Alpha-9(10)-EpODE | | 295.23 | | 6.49 | | 1.38 | | 1.08 | | < 0.001 | | pos |
|  | |  | | Nuatigenin | | 431.32 | | 8.03 | | 1.38 | | 1.11 | | < 0.001 | | pos |
|  | |  | | 9-hydroxy-10-oxo-12(Z)-octadecenoic acid | | 335.22 | | 6.68 | | 1.35 | | 1.05 | | < 0.001 | | pos |
|  | |  | | 1-ACETYLPIPERIDINE | | 128.11 | | 3.56 | | 1.35 | | 1.19 | | 0.040 | | pos |
|  | |  | | (±)12,13-DiHOME | | 337.23 | | 6.76 | | 1.34 | | 1.06 | | < 0.001 | | pos |
|  | |  | | Coumestrol | | 269.04 | | 5.34 | | 1.33 | | 1.18 | | 0.001 | | pos |
|  | |  | | Orcinol | | 125.06 | | 2.67 | | 1.28 | | 1.12 | | < 0.001 | | pos |
|  | |  | | Tetranor-12(R)-HETE | | 267.19 | | 5.89 | | 1.25 | | 1.08 | | < 0.001 | | pos |
|  | |  | | R-Palmitoyl-(2-methyl) Ethanolamide | | 314.30 | | 7.48 | | 1.17 | | 1.09 | | 0.008 | | pos |
|  | |  | | Stigmasta-5,7-dien-3beta-ol | | 413.38 | | 10.27 | | 1.17 | | 1.12 | | 0.009 | | pos |
|  | |  | | 9,10-epoxy-13-hydroxy-11-octadecenoic acid | | 313.24 | | 6.49 | | 1.15 | | 1.04 | | < 0.001 | | pos |
|  | |  | | TG (12:0/13:0/20:5(5Z,8Z,11Z,14Z,17Z))[iso6] | | 755.61 | | 11.02 | | 1.06 | | 1.16 | | 0.021 | | pos |
|  | |  | | 7S,8S-DiHOTrE | | 311.22 | | 5.61 | | 1.04 | | 1.04 | | < 0.001 | | pos |
|  | |  | | Glycerophospho-N-Palmitoyl Ethanolamine | | 454.29 | | 9.49 | | 1.01 | | 1.07 | | 0.001 | | pos |
|  | |  | | PE (16:0/0:0) | | 454.29 | | 8.59 | | 1.01 | | 1.05 | | 0.004 | | pos |
|  | |  | | PG (17:1(9Z)/0:0) | | 497.29 | | 10.39 | | 1.01 | | 1.07 | | 0.008 | | pos |
|  | |  | | SIMMONDSIN-2'-FERULATE | | 550.19 | | 8.11 | | 1.11 | | 0.89 | | 0.001 | | neg |
|  | |  | | P-Salicylic acid | | 137.02 | | 4.20 | | 1.17 | | 1.08 | | < 0.001 | | neg |
|  | |  | | MEDICA 16 | | 341.27 | | 8.94 | | 1.17 | | 1.09 | | < 0.001 | | neg |
| Diets: S, steam-flaked corn and corn silage diet; L, sugar beet pulp and alfalfa silage diet; HMDB, human metabolome database; M/Z, mass-to-charge ratio; FC, fold change; VIP, variable importance in the projection; pos, in positive ion mode; neg, in negative ion mode. | | | | | | | | | | | | | | | | |

| **Table S7 Significantly differential metabolites between the glucogenic diet S and C after 48 h *in vitro* fermentation with rumen fluid of dairy cows** | | | | | | | | | | | | | | | | |
| --- | --- | --- | --- | --- | --- | --- | --- | --- | --- | --- | --- | --- | --- | --- | --- | --- |
| **HMDB Superclass** | **HMDB Subclass** | | **Metabolite** | | | **M/Z** | | **Retention time** | | **VIP** | | **FC(C/S)** | | ***P*-value** | **Mode** | |
| Lipids and lipid-like molecules | | Fatty acids and conjugates | | Ethylmalonic acid | 131.04 | | 0.78 | | 1.10 | | 0.96 | | 0.034 | | | neg |
|  | |  | | 11Z-Eicosenoic acid | 355.28 | | 9.19 | | 1.12 | | 1.03 | | 0.007 | | | neg |
|  | |  | | Docosatrienoic acid | 357.28 | | 8.56 | | 2.52 | | 1.26 | | 0.034 | | | pos |
|  | |  | | Elaidic Acid | 283.26 | | 8.50 | | 1.34 | | 1.02 | | 0.005 | | | pos |
|  | |  | | Floionolic acid | 315.25 | | 9.73 | | 1.19 | | 1.02 | | 0.045 | | | pos |
|  | |  | | 5-Hexyl-2-furanhexanoic acid | 289.18 | | 5.85 | | 1.03 | | 1.02 | | 0.026 | | | pos |
|  | | Triterpenoids | | Camellenodiol | 463.34 | | 9.97 | | 2.30 | | 1.16 | | < 0.001 | | | neg |
|  | |  | | Ganoderic acid beta | 545.31 | | 8.51 | | 1.27 | | 1.02 | | 0.017 | | | neg |
|  | |  | | Lucidenic acid E2 | 539.26 | | 9.24 | | 1.33 | | 1.02 | | 0.003 | | | pos |
|  | |  | | Medicoside C | 461.25 | | 7.59 | | 1.13 | | 1.02 | | 0.010 | | | pos |
|  | | Glycerophosphates | | 1-(9Z-tetradecenoyl)-glycero-3-phosphate | 379.19 | | 8.11 | | 2.13 | | 0.84 | | 0.007 | | | neg |
|  | |  | | 1-tetradecanoyl-sn-glycero-3-phosphate | 365.21 | | 8.67 | | 2.45 | | 0.89 | | 0.005 | | | pos |
|  | |  | | LysoPA(P-16:0e/0:0) | 417.24 | | 9.12 | | 2.56 | | 1.19 | | 0.021 | | | pos |
|  | | Monoterpenoids | | (1beta,2beta,5beta) -p-Menth-3-ene-1,2,5-triol | 231.12 | | 4.67 | | 1.04 | | 0.97 | | 0.004 | | | neg |
|  | |  | | Piperitol | 155.14 | | 6.10 | | 3.11 | | 1.54 | | 0.016 | | | pos |
|  | |  | | Alpha-Terpineol propanoate | 211.17 | | 7.24 | | 1.59 | | 1.10 | | 0.037 | | | pos |
|  | | Sesquiterpenoids | | (1(10)E,4a,5E)-1(10),5-Germacradiene-12-acetoxy-4,11-diol | 295.19 | | 7.05 | | 2.40 | | 1.45 | | 0.043 | | | neg |
|  | |  | | 7(14)-Bisabolene-2,3,10,11-tetrol | 255.19 | | 6.43 | | 1.52 | | 1.06 | | 0.018 | | | pos |
|  | |  | | S-Japonin | 378.21 | | 9.79 | | 1.23 | | 0.96 | | 0.049 | | | pos |
|  | | Ergostane steroids | | (24R)-Ergost-4-ene-3,6-dione | 457.33 | | 9.53 | | 1.27 | | 1.06 | | 0.014 | | | neg |
|  | |  | | Delta 8,14 -Sterol | 393.35 | | 12.76 | | 1.16 | | 0.97 | | 0.049 | | | pos |
|  | | Glycerophosphoethanolamines | | PE (14:1(9Z)/16:1(9Z)) | 660.46 | | 10.33 | | 2.56 | | 1.17 | | 0.040 | | | pos |
|  | |  | | LysoPE (0:0/16:0) | 436.28 | | 8.40 | | 1.85 | | 1.09 | | 0.009 | | | pos |
|  | | Diterpenoids | | (13R,14R)-7-Labdene-13,14,15-triol | 357.30 | | 8.36 | | 1.39 | | 1.02 | | 0.001 | | | pos |
|  | | Eicosanoids | | Carboprost Tromethamine | 367.25 | | 7.62 | | 1.57 | | 1.07 | | 0.011 | | | neg |
|  | | Fatty acyl glycosides | | 4-Hydroxyproline galactoside | 258.10 | | 3.23 | | 2.07 | | 1.08 | | 0.002 | | | pos |
|  | | Fatty alcohols | | 13-Heptadecyn-1-ol | 253.25 | | 8.48 | | 1.21 | | 1.03 | | 0.042 | | | pos |
|  | | Fatty amides | | Palmitoyl Serinol | 330.30 | | 8.94 | | 3.04 | | 0.80 | | 0.004 | | | pos |
|  | | Steroid esters | | Physapubescin | 553.28 | | 7.95 | | 1.45 | | 1.04 | | 0.026 | | | pos |
|  | |  | |  |  | |  | |  | |  | |  | | |  |
| **Table S7 (continued)** | | | | | | | | | | | | | | | | |
| **HMDB Superclass** | **HMDB Subclass** | | **Metabolite** | | | **M/Z** | | **Retention time** | | **VIP** | | **FC(C/S)** | | ***P*-value** | **Mode** | |
|  | | Steroid lactones | | Physagulin C | 565.24 | | 8.41 | | 1.41 | | 1.03 | | 0.040 | | | pos |
|  | | Terpene lactones | | Isoalantolactone | 233.15 | | 6.28 | | 1.04 | | 1.02 | | 0.040 | | | pos |
|  | | Unclassified | | 11,13-Dihydrotaraxinic acid glucosyl ester | 407.17 | | 7.10 | | 3.30 | | 0.54 | | 0.029 | | | neg |
| Organic acids and derivatives | | Amino acids, peptides, and analogues | | Captopril-cysteine disulfide | 371.05 | | 8.00 | | 2.38 | | 1.30 | | 0.014 | | | neg |
|  | |  | | 2-Hepteneoylglycine | 230.10 | | 3.07 | | 2.66 | | 1.22 | | < 0.001 | | | neg |
|  | |  | | Tetrahydrodipicolinate | 204.09 | | 1.57 | | 2.29 | | 1.11 | | < 0.001 | | | pos |
|  | |  | | Valyl-Proline | 197.13 | | 3.02 | | 1.90 | | 1.09 | | 0.008 | | | pos |
|  | |  | | Isoleucyl-Glutamate | 243.13 | | 1.41 | | 1.85 | | 1.05 | | 0.004 | | | pos |
|  | |  | | Dynorphin B (10-13) | 459.30 | | 3.72 | | 1.76 | | 0.93 | | 0.028 | | | pos |
|  | |  | | 5-Hydroxyindoleacetylglycine | 213.07 | | 3.25 | | 1.65 | | 1.10 | | 0.033 | | | pos |
|  | |  | | Glutamylleucine | 243.13 | | 0.76 | | 1.44 | | 1.03 | | 0.002 | | | pos |
|  | |  | | Alanyl-Phenylalanine | 219.11 | | 2.68 | | 1.38 | | 1.07 | | 0.039 | | | pos |
|  | |  | | 5-hydroxyhexanoylglycine | 212.09 | | 0.75 | | 1.36 | | 1.03 | | 0.003 | | | pos |
|  | |  | | D-Pipecolic acid | 130.09 | | 0.68 | | 1.36 | | 1.03 | | 0.005 | | | pos |
|  | |  | | Phenylalanylproline | 245.13 | | 3.54 | | 1.21 | | 1.03 | | 0.031 | | | pos |
|  | | Hybrid peptides | | Beta-Alanyl-L-lysine | 476.32 | | 8.31 | | 1.87 | | 1.07 | | 0.003 | | | pos |
|  | |  | | Astin I | 516.20 | | 2.93 | | 1.45 | | 1.05 | | 0.038 | | | pos |
|  | | Carboxylic acid derivatives | | Dihydroceramide | 374.29 | | 8.95 | | 2.76 | | 0.78 | | 0.017 | | | neg |
|  | | Organic carbonic acids | | Moracin L | 342.13 | | 5.06 | | 2.02 | | 1.17 | | 0.037 | | | pos |
|  | | Fumonisins | | Fumonisin B1 | 722.40 | | 5.05 | | 1.28 | | 1.02 | | 0.005 | | | pos |
| Organic oxygen compounds | | Carbohydrates and carbohydrate conjugates | | Dihydromaleimide beta-D-glucoside | 260.08 | | 1.40 | | 1.43 | | 1.06 | | 0.049 | | | neg |
|  | |  | | Pisatoside | 260.08 | | 1.63 | | 1.85 | | 1.08 | | 0.008 | | | neg |
|  | |  | | Tyramine glucuronide | 294.10 | | 2.97 | | 1.82 | | 1.05 | | 0.006 | | | neg |
|  | |  | | N-Acetylneuraminic acid | 274.09 | | 2.80 | | 1.72 | | 1.08 | | 0.047 | | | pos |
|  | |  | | Lotaustralin | 226.11 | | 2.79 | | 1.20 | | 1.02 | | 0.014 | | | pos |
|  | |  | | Indican | 296.11 | | 2.97 | | 1.92 | | 1.05 | | 0.003 | | | pos |
|  | | Ethers | | Digoxigenin monodigitoxoside | 565.28 | | 8.94 | | 1.84 | | 1.05 | | 0.001 | | | pos |
|  | |  | | Heptaethylene glycol | 327.20 | | 7.69 | | 1.09 | | 1.02 | | 0.024 | | | pos |
|  | | Carbonyl compounds | | 1-Hydroxyepiacorone | 289.12 | | 3.60 | | 1.90 | | 1.10 | | 0.008 | | | neg |
| Organoheterocyclic compounds | | 1,3-dioxolanes | | Valeraldehyde propyleneglycol acetal | 125.10 | | 4.29 | | 1.23 | | 1.03 | | 0.050 | | | neg |
|  | | Gamma butyrolactones | | 3-Hydroxyadipic acid 3,6-lactone | 143.04 | | 1.62 | | 1.62 | | 0.89 | | 0.018 | | | neg |
|  | |  | |  |  | |  | |  | |  | |  | | |  |
| **Table S7 (continued)** | | | | | | | | | | | | | | | | |
| **HMDB Superclass** | **HMDB Subclass** | | **Metabolite** | | | **M/Z** | | **Retention time** | | **VIP** | | **FC(C/S)** | | ***P*-value** | **Mode** | |
|  | | Hydroxyindoles | | 11-beta-Hydroxyandrosterone-3-glucuronide | 463.24 | | 8.09 | | 3.93 | | 0.49 | | 0.003 | | | neg |
|  | | Pterins and derivatives | | 2-Amino-4-oxo-6-(1',2'-dioxoprolyl)-7,8-dihydroxypteridine | 288.03 | | 1.24 | | 1.23 | | 0.96 | | 0.018 | | | neg |
|  | | Tryptamines and derivatives | | N-Methyl-1H-indole-3-propanamide | 203.12 | | 4.31 | | 1.25 | | 1.03 | | 0.016 | | | pos |
|  | |  | | Jasmine ketolactone | 253.11 | | 5.43 | | 1.79 | | 1.17 | | 0.027 | | | neg |
|  | |  | | Isoquinoline | 130.06 | | 4.87 | | 1.94 | | 1.07 | | 0.008 | | | pos |
|  | |  | | 3alpha-Hydroxyoreadone | 285.17 | | 3.39 | | 1.63 | | 1.04 | | 0.002 | | | pos |
| Phenylpropanoids and polyketides | | Flavonoid glycosides | | 6''-Malonylcosmosiin | 517.10 | | 4.90 | | 1.21 | | 0.97 | | 0.007 | | | neg |
|  | |  | | 1-Hydroxyibuprofen | 221.12 | | 5.74 | | 1.79 | | 0.89 | | 0.026 | | | neg |
|  | |  | | 3-(3,4-Dihydroxyphenyl)-2-methylpropionic acid | 195.07 | | 5.42 | | 1.63 | | 0.95 | | 0.016 | | | neg |
|  | |  | | (Z)-7-Hexadecen-1,16-olide | 294.24 | | 11.13 | | 1.61 | | 0.94 | | 0.046 | | | pos |
|  | |  | | Neosaxitoxin | 280.12 | | 4.14 | | 2.76 | | 1.19 | | 0.003 | | | pos |
| Benzenoids | | Benzenediols | | 3-(3,4-dihydroxyphenyl)-N-[2-(4-hydroxyphenyl) ethyl] propanimidic acid | 338.08 | | 1.28 | | 1.31 | | 0.95 | | 0.014 | | | neg |
|  | |  | | 3-Phenylpropyl acetate | 179.11 | | 3.66 | | 1.15 | | 0.97 | | 0.024 | | | pos |
|  | |  | | 1,1-Dimethoxy-2-phenylethane | 167.11 | | 3.59 | | 1.19 | | 1.04 | | 0.034 | | | pos |
| Alkaloids and derivatives | |  | | Dextrorphan O-glucuronide | 398.20 | | 8.45 | | 1.37 | | 1.03 | | 0.003 | | | pos |
| Others | |  | | C-2 Ceramide | 342.30 | | 9.05 | | 1.41 | | 1.04 | | 0.008 | | | pos |
|  | |  | | 3-Methyl-1-phenyl-1-butanone | 163.11 | | 5.69 | | 1.41 | | 0.94 | | 0.030 | | | pos |
|  | |  | | Amprotropine | 308.22 | | 4.74 | | 1.84 | | 1.06 | | 0.037 | | | pos |
|  | |  | | L-Homotyrosine | 196.10 | | 1.69 | | 1.70 | | 1.05 | | 0.000 | | | pos |
|  | |  | | 3-Indolepropionic acid | 190.09 | | 4.87 | | 1.65 | | 1.04 | | 0.017 | | | pos |
|  | |  | | Indole-3-carboxaldehyde | 354.15 | | 3.15 | | 1.57 | | 1.04 | | 0.002 | | | pos |
|  | |  | | Pyridoxine (Vitamin B6) | 170.08 | | 0.73 | | 1.46 | | 1.03 | | 0.004 | | | pos |
|  | |  | | Trans-EKODE-(E)-Ib | 311.22 | | 7.41 | | 1.39 | | 1.03 | | 0.012 | | | pos |
|  | |  | | PHENACYLAMINE | 136.07 | | 4.08 | | 1.39 | | 0.96 | | 0.048 | | | pos |
|  | |  | | PE (18:1(9Z)/0:0) | 480.31 | | 8.69 | | 1.36 | | 1.03 | | 0.031 | | | pos |
|  | |  | | Polyoxyethylene (600) mono- ricinoleate | 341.30 | | 8.44 | | 1.27 | | 1.02 | | 0.024 | | | pos |
|  | |  | | 9-hydroxy-12Z-octadecenoic acid | 299.26 | | 8.47 | | 1.10 | | 1.01 | | 0.016 | | | pos |
|  | |  | |  |  | |  | |  | |  | |  | | |  |
| **Table S7 (continued)** | | | | | | | | | | | | | | | | |
| **HMDB Superclass** | **HMDB Subclass** | | **Metabolite** | | | **M/Z** | | **Retention time** | | **VIP** | | **FC(C/S)** | | ***P*-value** | **Mode** | |
|  | |  | | 9,10-Epoxy-18-hydroxystearate | 315.25 | | 8.36 | | 1.03 | | 1.03 | | 0.033 | | | pos |
|  | |  | | 12-OPDA | 293.21 | | 7.02 | | 1.02 | | 1.02 | | 0.020 | | | pos |
| Diets: S, steam-flaked corn and corn silage diet; C, corn and corn silage diet; HMDB, human metabolome database; M/Z, mass-to-charge ratio; FC, fold change; VIP, variable importance in the projection; pos, in positive ion mode; neg, in negative ion mode. | | | | | | | | | | | | | | | | |
